# Exploration of pyrazine-embedded antiaromatic polycyclic hydrocarbons generated by solution and on-surface azomethine ylide homocoupling

**DOI:** 10.1038/s41467-017-01934-1

**Published:** 2017-12-05

**Authors:** Xiao-Ye Wang, Marcus Richter, Yuanqin He, Jonas Björk, Alexander Riss, Raju Rajesh, Manuela Garnica, Felix Hennersdorf, Jan J. Weigand, Akimitsu Narita, Reinhard Berger, Xinliang Feng, Willi Auwärter, Johannes V. Barth, Carlos-Andres Palma, Klaus Müllen

**Affiliations:** 1 Max Planck Institute for Polymer Research, Ackermannweg 10, 55128 Mainz, Germany; 2 Department for Molecular Functional Materials, Center for Advancing Electronics Dresden (cfaed), Dresden University of Technology, Mommsenstr. 4, 01062 Dresden, Germany; 3 Institute for Advanced Study, Technical University of Munich, Lichtenbergstr. 2a, 85748 Garching, Germany; 4 Physik-Department E20, Technical University of Munich, James-Franck-Str. 1, 85748 Garching, Germany; 5 Department of Physics, Chemistry and Biology, IFM, Linköping University, 58183 Linköping, Sweden; 6 Chair of Inorganic Molecular Chemistry, Dresden University of Technology, Mommsenstr. 4, 01062 Dresden, Germany

## Abstract

Nanographenes, namely polycyclic aromatic hydrocarbons (PAHs) with nanoscale dimensions (>1 nm), are atomically precise cutouts from graphene. They represent prime models to enhance the scope of chemical and physical properties of graphene through structural modulation and functionalization. Defined nitrogen doping in nanographenes is particularly attractive due to its potential for increasing the number of π-electrons, with the possibility of introducing localized antiaromatic ring elements. Herein we present azomethine ylide homocoupling as a strategy to afford internally nitrogen-doped, non-planar PAH in solution and planar nanographene on surfaces, with central pyrazine rings. Localized antiaromaticity of the central ring is indicated by optical absorption spectroscopy in conjunction with theoretical calculations. Our strategy opens up methods for chemically tailoring graphene and nanographenes, modified by antiaromatic dopants.

## Abstract (web summary)

Polyaromatic hydrocarbons can be precisely manipulated to yield ever more complex and discrete graphene analogs, such as nanographenes. Here, the authors use azomethine ylide homocoupling to insert an antiaromatic pyrazine ring into the core of a nanographene, and characterize the molecule’s unique electronic character.

## Introduction

**Fig. 1:**
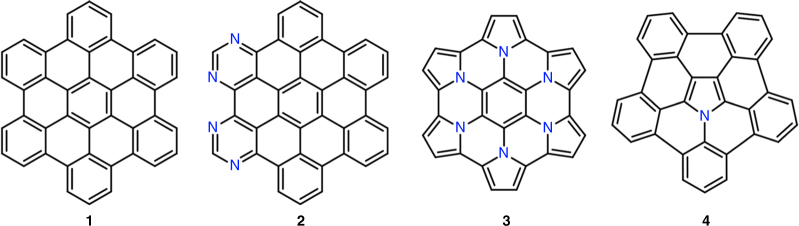
Chemical structures of pristine and N-doped nanographenes. Hexa-*peri*-hexabenzocoronene (HBC) **1**, periphery-doped tetraaza-HBC **2**, azacoronene derivatives **3**, and azacorannulene **4**

Together with the advent of graphene science, an important chapter in organic chemistry has opened exploring the synthesis of extended polycyclic aromatic hydrocarbon (PAH) systems. Large PAHs, as represented by hexa-*peri*-hexabenzocoronene (HBC, **1**) with π-conjugated structures extending over 1 nm, can be regarded as structurally defined, nanoscale cutouts of graphene, designated nanographene molecules^1–3^. Such systems can serve as platforms both for investigating the physical and chemical properties of graphene upon heteroatom-doping, as well as for the development of semiconducting materials (see Fig. 1). Doped nanographenes are similarly relevant for the study of aromaticity and antiaromaticity, which are fundamental concepts of organic chemistry^4,5^. As proposed by Hückel^6^ and later by Frost and Musulin^7^, as well as Breslow^8^, π-conjugated cycles with [4n + 2] π-electrons show aromatic character with strong stabilization while cycles with [4n] π-electrons are antiaromatic and destabilized. Despite a few theoretical suggestions on local antiaromatic “defects”, or “dopants”, in graphene^9,10^ and rapidly increasing variety of nanographene molecules, well-defined nanographene molecules with a pronounced antiaromatic ring, i.e., a local antiaromatic element, remain challenging targets to be explored.

Nitrogen (N)-doped nanographenes have attracted considerable attention due to promising electronic and magnetic properties^11^ offering potential applications in metal-free catalysis^12,13^ and sensing^14,15^. However, the existing synthetic protocols do not allow the precise control of the doping level and heteroatom positioning. The chemical nature of nitrogen dopants (i.e., pyridinic, pyrrolic, and “graphitic” N atoms) remains largely undefined^16^, restricting the fine-tuning of the resulting properties as well as reliable structure-property relationship elucidation. In this regard, structurally well-defined N-doped nanographene molecules offer an opportunity to accurately investigate N-doping effects. Notwithstanding, N-doping of defined nanographenes has been predominantly limited to pyridinic^17–19^ (e.g., compound **2** ^20^ in Fig. 1) and pyrrolic (e.g., compounds **3** ^21^ and **4** ^22,23^) N atoms, which only resulted in replacing benzene rings with other N-containing 6π aromatic rings, such as pyrimidines and pyrroles.

To develop nanographenes with strong antiaromatic ring dopants, we consider the possibility of incorporating a pyrazine structure inside the aromatic core, which employs two “graphitic” N atoms in the framework, thus providing additional π-electrons when compared to a benzene ring. 1,4-Disubstituted pyrazine entails intriguing chemistry, expressing 8π antiaromatic (neutral), 7π non-aromatic (cationic), or 6π aromatic (dicationic) properties, depending on its oxidation state. Thus, a pyrazine ring embedded in a carbonaceous hexagonal structure serves as a localized antiaromatic ring dopant, influencing ring and perimeter currents^24,25^ with the potential to give rise to new types of 2D materials with unconventional semiconducting properties.

Here, we report in situ solution synthesis and spectroscopy of the partially fused, pyrazine-incorporated hexabenzoperylene (HBP) derivative **6a** as well as on-surface synthesis of the diaza-HBC **7c** with a completely embedded pyrazine core. N-doped HBP **6a** features an 8π antiaromatic core structure, as documented by ^1^H NMR and UV–vis absorption spectroscopy together with density functional theory (DFT) calculations. Notably, the solution chemistry of polycyclic aromatic azomethine ylides (PAMYs)^26^ can be successfully adapted on the Ag(111) surface, achieving the synthesis of **7c** through surface-assisted homocoupling of the dibenzo-9*a*-azaphenalene (DBAP) salt **5c** and concomitant cyclodehydrogenation. High-resolution scanning tunneling microscopy (STM) and frequency-modulated atomic force microscopy (FM-AFM)^27^ unambiguously elucidate the chemical structure of diaza-HBC **7c**. Moreover, computational modeling using DFT indicates that **7c** conserves its 8π character on the Ag(111) surface. Nucleus-independent chemical shift (NICS)^28^ calculations on **6a** and **7c** reveal high positive values at the center and 1 Å above the center of the central pyrazine rings (NICS(0) and NICS(1) values, respectively), indicative of antiaromaticity. These results demonstrate the antiaromatic nature of pyrazine-doped nanographene molecules and provide an on-surface synthesis route to N-doping nanographene materials.

## Results

### Solution synthesis and characterizations

**Fig. 2:**
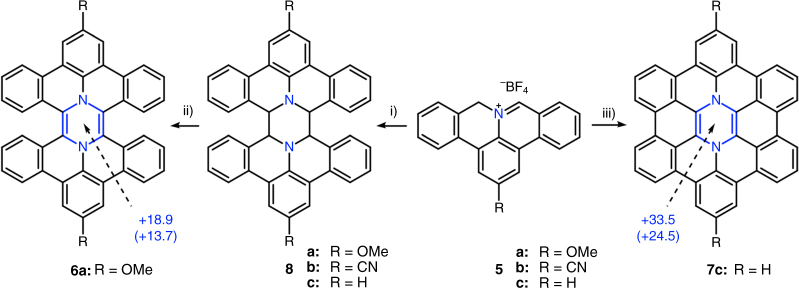
Synthesis of diaza-HBP **6a** in solution and internally N-doped diaza-HBC **7c** upon interfacial confinement. Reagents and conditions: (i) tributylamine, DMSO, 190 °C; (ii) DDQ, C_2_D_2_Cl_4_, 100 °C; (iii) in vacuo on the Ag(111) surface, 270 °C. The calculated nucleus-independent chemical shifts (NICS) values are depicted in blue (unit: ppm), indicating the antiaromatic nature of the pyrazine ring (in parenthesis the NICS(1) value, see text and Supplementary Fig. 1)

**Fig. 3:**
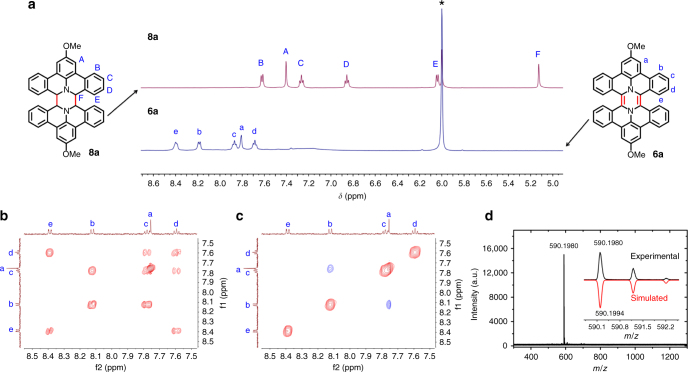
Structural characterizations of the in situ generated diaza-HBP **6a**. **a** ^1^H NMR spectra of **8a** and **6a** together with their structural assignments (*C_2_HDCl_4_). Compared to precursor **8a**, the signals of **6a** were down-field shifted, corroborating the extension of the π-conjugation. **b** Aromatic region of the ^1^H–^1^H COSY spectrum of **6a** in C_2_D_2_Cl_4_. **c** Aromatic region of the ^1^H–^1^H NOESY spectrum of **6a** in C_2_D_2_Cl_4_. **d** High-resolution MALDI-TOF MS spectrum of **6a**, with an inset showing its experimental (black line) and simulated isotopic distributions (red line)

**Fig. 4:**
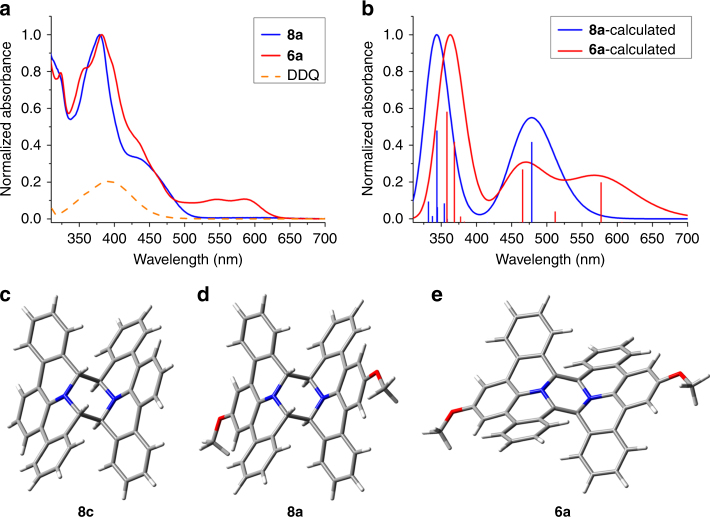
Photophysical investigations. **a** Experimental and **b** TDDFT-simulated absorption spectra of **6a** and its precursor **8a**. The simulated spectra of **6a** showed the appearance of a new band at 577 nm, in accordance with the experimental observation. **c** X-ray single-crystal structure of **8c**. **d** DFT-optimized structure of **8a**. **e** DFT-optimized structure of **6a**

The synthesis of pyrazine-embedded HBP **6a** (Fig. 2) was carried out based on the chemistry of DBAP, which has been employed to construct N-doped PAHs such as dibenzo[*d*,*k*]ullazines and 6*b* ^2^-azapentabenzo[*bc*,*ef*,*hi*,*kl*,*no*]corannulenes^22,26,29,30^. DBAP salts **5a-c** with different substituents were first synthesized according to our previous method^26^. They were then dimerized to form precursors **8a–c** (Fig. 2) by treatment with a large excess of tributylamine in dry dimethyl sulfoxide (DMSO) under argon at 190 °C. Oxidation of **8a** with excess 2,3-dichloro-5,6-dicyano-1,4-benzoquinone (DDQ) in dry C_2_D_2_Cl_4_ afforded diaza-HBP **6a**, as characterized by ^1^H NMR and mass spectroscopy (MS) analyses (Fig. 3). Diaza-HBP **6a** turned out to be extremely unstable probably because of the antiaromatic pyrazine core, and thus the oxidation step was performed in situ in a sealed NMR tube under exclusion of air. Oxidation of other precursors **8b** and **8c** were also attempted with the same method, but failed to provide corresponding diaza-HBP derivatives according to ^1^H NMR analysis, suggesting that the electron-donating methoxy groups of **8a** played an important role in the reaction^31^. As shown in Fig. 3a, the disappearance of the characteristic signal from H_F_ (5.13 ppm) of **8a** indicated complete conversion of the starting material. All the signals in the aromatic region of **6a** were down-field shifted compared to those of **8a**, in agreement with the extension of the π-conjugation upon dehydrogenation. Notably, proton H_E_ of **8a** is above the neighboring benzene plane according to the optimized geometry by DFT (Fig. 4) and thus is strongly shielded. In contrast, after dehydrogenation of **8a** via DDQ, the proton H_e_ of **6a** is shifted away from the neighboring benzene into its deshielding region, in accordance with a down-field shift of H_e_ in the ^1^H NMR spectrum. Moreover, ^1^H–^1^H correlation spectroscopy (COSY) and ^1^H-^1^H nuclear Overhauser effect spectroscopy (NOESY) analyses (Fig. 3b, c) allowed unambiguous assignments of the proton signals of **6a**. The structure was further characterized by ^1^H–^13^C heteronuclear single-quantum correlation (HSQC) and ^1^H–^13^C heteronuclear multiple-bond correlation (HMBC) spectroscopies. The chemical identity of **6a** was confirmed by high-resolution matrix-assisted laser desorption/ionization time-of-flight (MALDI-TOF) MS, which showed a matching isotopic pattern starting at *m*/*z* = 590.1980, in agreement with the theoretical value of 590.1994 (Fig. 3d).

To investigate the photophysical properties of **6a**, we prepared diaza-HBP **6a** in situ by carrying out the oxidation reaction in a sealed quartz cell and recorded the absorption and photoluminescence spectra. As shown in Fig. 4a, dimer precursor **8a** exhibited an absorption maximum at *λ*_max_ = 390 nm, and an absorption onset at ≈500 nm. After dehydrogenation, the absorption maximum barely changed, but a new band emerged in the range of 500 to 630 nm, suggesting an extended π-conjugation. To further understand the photophysical properties, DFT calculations were performed on both **8a** and **6a**. In order to determine the exact molecular geometry of **8a**, growth of single crystals of **8a–c** was pursued and a single crystal of **8c** suitable for X-ray analysis was successfully obtained, revealing an *anti*-folded structure (Fig. 4c). Thus, the *anti*-folded geometry was employed as input geometry for the DFT optimization of similar precursors (Fig. 4d). DFT studies on dehydrogenated **6a** indicate that the *anti*-folded conformation (Fig. 4e) is energetically preferred compared to the twisted one. Time-dependent DFT (TDDFT) calculations of **6a** and **8a** indicated that the emerging absorption band of **6a** can be attributed to the HOMO→LUMO + 2 transition (Fig. 4b and Supplementary Tables 1–3). The HOMO→LUMO transition of **6a** is symmetry forbidden, and **6a** is non-emissive, in agreement with previous reports on antiaromatic systems^32,33^.

### On-surface synthesis of 7c

**Fig. 5:**
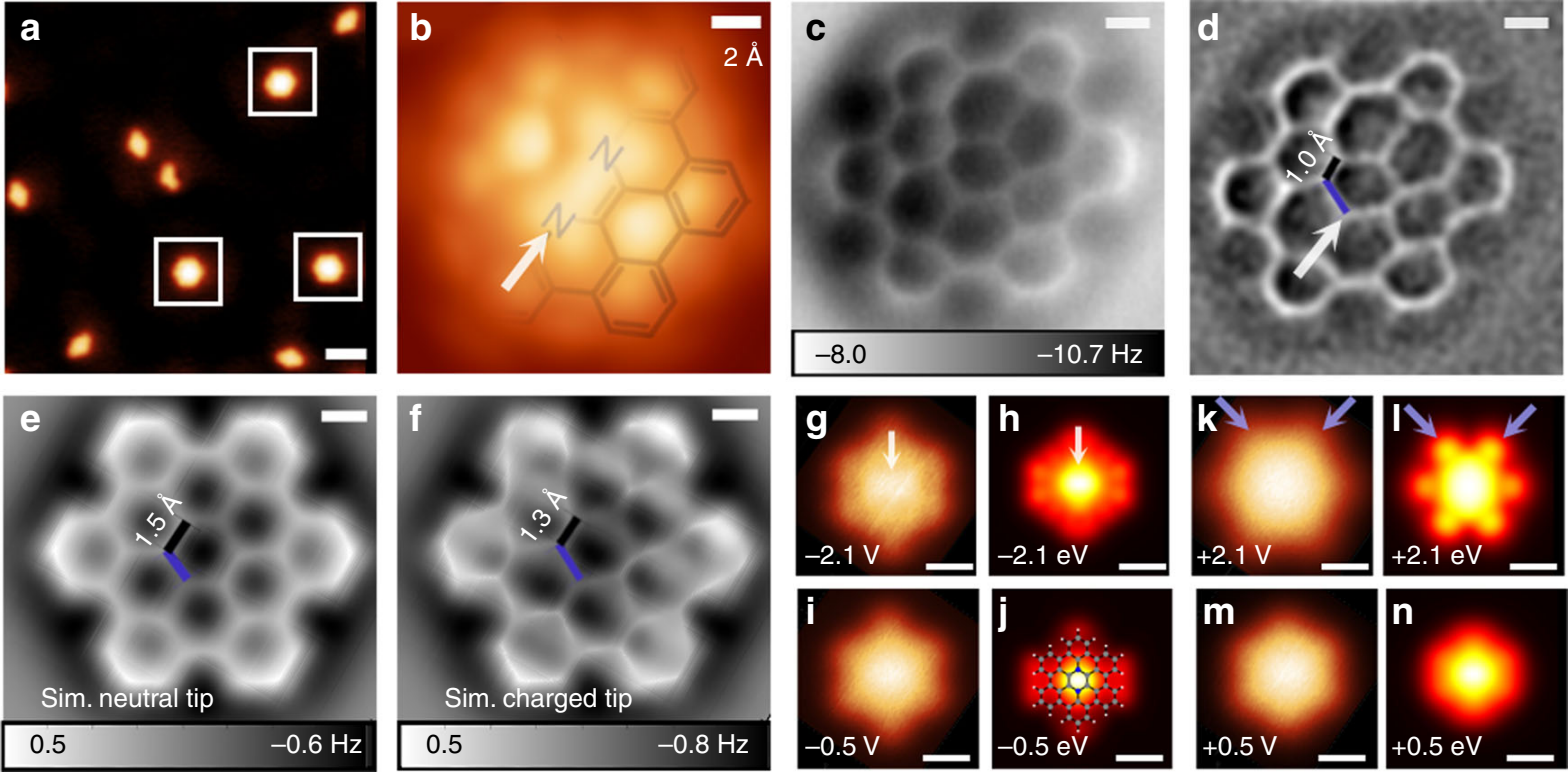
On-surface homocoupling of precursor **5c** yields fully planar and neutral diaza-HBC **7c** on Ag(111). **a** Scanning tunneling microscopy (STM) overview of reaction product and unreacted species on Ag(111). *V*_s_ = 30 mV, *I*_t_ = 10 pA. **b** Constant-height STM data of diaza-HBC with partially superposed molecular model. *V*_s_ = 5 mV. **c** Frequency-modulated atomic force microscopy (FM-AFM) and **d** Laplace-filtered FM-AFM data reveal details in the molecular structure. Apparent C = C distance 1.0 Å (black), and N–C 1.8 Å (blue). **e** FM-AFM simulations of **7c** employing **a** neutral tip. Apparent C = C distance 1.5 Å (black), N–C 1.5 Å (blue). **f** FM-AFM simulations employing a negatively charged probe tip. Apparent C = C distance 1.3 Å (black), N-C 1.9 Å (blue). For simulation parameters see Methods. **g**–**n** Constant-height STM data (**g**, **i**, **k**, **m**) at different biases and corresponding (**h**, **j**, **l**, **n**) DFT simulations. White arrows point to N atoms oriented along the diaza-HBC symmetry axis. Scale bars (**a**) 20 Å, (**b**–**f**) 2 Å, (**g**–**n**) 5 Å

Despite generation of diaza-HBP **6a** through solution synthesis, further characterization of **6a** was restricted by its low stability. In addition, attempts to oxidize intermediate product **8a–c** directly to diaza-HBC derivatives resulted in insoluble solids, which could not be characterized. This led us to adapt the solution chemistry of PAMYs to on-surface synthesis^34–37^, which offers today a new approach to organic chemistry. We thus deposited **5c** on Ag(111) by molecular beam evaporation (Methods section and Supplementary Fig. 2). Upon annealing to 270 °C, small quantities of diaza-HBC **7c** were detected as displayed in Fig. 5a. Unlike solution synthesis (Fig. 2), no intermediate product **8c** was identified after annealing the deposited **5c** in several ultra-high vacuum (UHV) preparations on Ag(111), Ag(100), Cu(111), and boron nitride (BN) on Cu(111), suggesting that homocoupling and dehydrogenation occur synergistically at the employed reaction temperatures. Notably, **7c** was detected on BN/Cu(111) templates^38^ at similar annealing temperatures in trace-quantities (Supplementary Fig. 3), indicating that the on-surface coupling strategy proceeds in a metal-free fashion, further motivating studies on reaction mechanisms and on-surface synthesis on insulators.

High-resolution, constant height STM (Fig. 5b) and FM-AFM (Fig. 5c, d), using CO-functionalized tips, confirmed **7c** on Ag(111). In FM-AFM data on Ag(111), the central pyrazine ring of **7c** (Fig. 5d) appears distorted. Observation of a *D*_2*h*_ symmetry points toward the nitrogen atoms sitting at the positions indicated by white arrows. Note that the predicted DFT C=C bond length of the central pyrazine ring is 1.40 Å vs. 1.42 Å for the N–C bond, and the molecule remains planar (z ± 0.05 Å) for calculated adsorption sites on Ag(111) (Supplementary Fig. 4). Bond lengths in AFM data typically undergo distorsions^27,39–41^ especially under the influence of electrostatic interactions with the CO tip^42–46^. This causes a distorted appearance of the central pyrazine ring. Quantitative evidence of these combined effects is summarized in the AFM frequency shift simulation including a DFT-derived electrostatic potential (Fig. 5e, f, see Methods section). Note how the apparent C=C bond length in the simulated image (1.3 Å) appears shorter than the apparent N–C bond length (1.9 Å, Fig. 5f), in good agreement with the experimental data (1.0 and 1.8 Å, respectively). When a neutral tip is employed for the AFM simulation the central pyrazine ring is fully symmetric, with apparent N–C and C=C lengths of 1.5 Å (Fig. 5e), confirming that the distortions in the FM-AFM data originate mostly from electrostatic effects.

Combined scanning probe data and theoretical investigations indicate that **7c** remains neutral (conserving its pyrazine 8π state) on the surface, which is a requirement for antiaromaticity. Bader charge analysis was employed to assess molecule-substrate charge transfer. As shown by NICS calculations on isolated molecules, a neutral species of **7c** implies an 8π pyrazine unit, which is antiaromatic in the isolated state. Upon oxidation, a positively charged “7π” radical cation state of the diaza-HBC **7c** displays significantly reduced antiaromaticity and a “6π” dication species exhibits an aromatic character (Supplementary Fig. 1). On Ag(111), theoretical charge analysis finds only marginal electron transfer to the substrate (the molecule is charged positively by less than + 0.14*e* for all calculated molecular configurations on Ag(111), Supplementary Fig. 4). Moreover, when trying to enforce molecular charging by removing electrons from the calculated system, the charge at the molecule was only increased to about + 0.8*e*, when the whole 29 Å × 25 Å × 22 Å unit cell was charged by + 10*e*. These theoretical analyses indicate that **7c** remains in the neutral “8π” state on Ag(111). The good correlation of the STM experimental data with Tersoff-Hamann simulations supports the validity of the theoretical conclusions (Fig. 5g, n and see Supplementary Fig. 5 for extended comparisons). It should be noted how at negative energies (voltages), the symmetry of the tunneling image follows the diaza-HBC **7c** hexagonal frame (marked by a white arrow, Fig. 5g, h). At high positive biases (between 0.9 and 2.1 eV, see Supplementary Fig. 5) the symmetry of the tunneling data is rotated (blue arrows, Fig. 5k, l) with respect to the diaza-HBC hexagonal frame, that is, tunneling is enhanced between the bay regions in the periphery of diaza-HBC. Notably, between −0.5 and 0.5 eV (Fig. 5i, j, m, n), both experiment and simulation follow the hexagonal symmetry of **7c** on Ag(111).

**Fig. 6:**
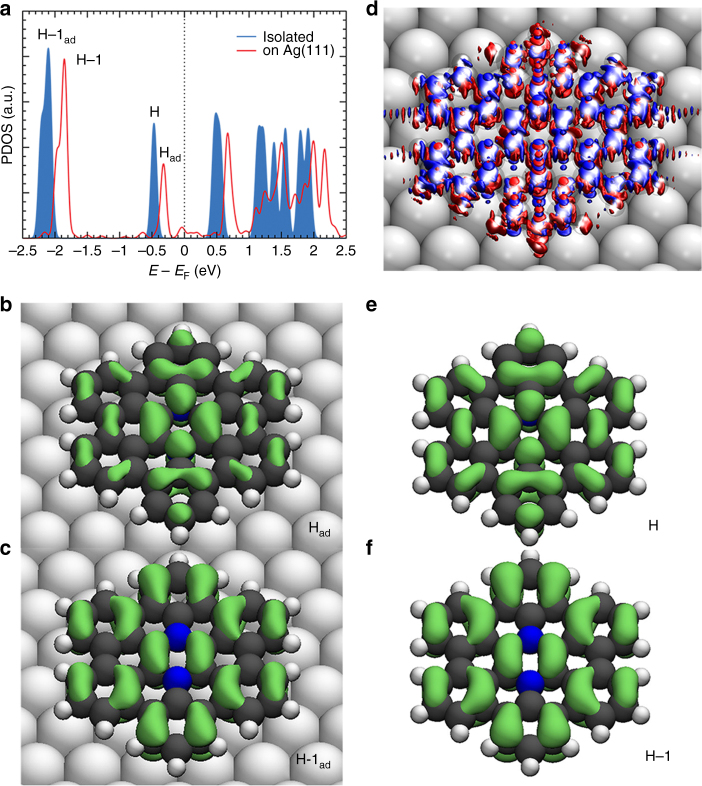
DFT electronic structure comparison of the isolated and Ag(111)-supported diaza-HBC **7c**. **a** Projected *p*_z_ contributions to the density of states of isolated **7c** (blue) and on Ag(111). The Fermi energy (*E*_F_) of the isolated molecule has been arbitrarily defined for comparison. **b**, **c** Contribution of the highest occupied molecular orbital **H**_**ad**_ (at −0.32 eV in **a**) and (**H-1**)_**ad**_ to **7c** on Ag(111). **d** Electron density difference between **7c** on Ag(111) and the isolated molecule plus isolated substrate. Red regions represent electron density depletion, whilst blue electron density gain. Atoms are rendered translucent. **e**, **f** Highest occupied molecular orbital **H** and **H-1** of isolated **7c**. These results suggest that antiaromatic character is preserved on Ag(111)

Having identified a neutral state of **7c** on Ag(111), comparison between **7c** on Ag(111) and the isolated antiaromatic species provides evidence on the preserved antiaromatic character of **7c** on Ag(111). Figure 6 depicts the site-projected density of states (PDOS) as well as plots for the individual Kohn-Sham orbitals. Only marginal hybridization resulting in mid-gap states is observed when the isolated molecule interacts with Ag(111) (Fig. 6a, red line). The mid-gap states do not appear to significantly influence the antiaromatic character of the adsorbed molecule: the HOMO (H) and HOMO-1 (H-1) signatures of isolated **7c** are reflected undisturbed at the surface (compare Fig. 4b, c, e, f). Instead, the electronic density difference plot (Fig. 6d) shows that the mid-gap states originate from homogeneous orbital contributions, including the role of rings which are considered to be aromatic (Supplementary Fig. 1). Note that scanning tunneling spectroscopy follows the major theoretical LDOS features including a pronounced broadening (Supplementary Fig. 6).

## Discussion

In summary, we have demonstrated the chemical synthesis of a partially fused PAH in solution and the on-surface synthesis of a fully planarized nanographene molecule with antiaromatic pyrazine dopants. Diaza-HBP **6a** has been achieved in solution and investigated by NMR, high-resolution MALDI-TOF MS and UV-vis absorption spectroscopy, while the fully cyclized diaza-HBC **7c** has been successfully fabricated on Ag(111) and characterized by STM and FM-AFM. We suggest that pyrazine rings are potential candidates to be included in 2D materials to spawn novel functional properties induced by breaking the conjugated lattice via antiaromatic ring dopants. Importantly, the chemistry of on-surface azomethine ylide homocoupling reported here opens up a new route to novel nanographene materials, especially graphene nanoribbons, on a large variety of substrates.

## Methods

### Procedure for the in situ preparation of **6a**

Inside an argon-filled glovebox, precursor **8a** (2.0 mg, 1.0 eq.), DDQ (15 mg, 20 eq.) and C_2_D_2_Cl_4_ (0.6 mL) were added sequentially into a sealable NMR tube. The mixture was heated at 100 °C for 1 min and cooled down to room temperature, and then analyzed by NMR spectroscopy. Synthetic procedures and characterization data of **8a–c** and **6a** are provided in the **Supplementary Methods** and Supplementary Figs. 7–45.

### STM and FM-AFM measurements

Precursor **5c** was sublimated between 200 °C and 220 °C at 10^−9^ mbar. Data was acquired in situ using a CreaTec STM/AFM (5.5 K) under ultra-high vacuum (10^−10^ mbar). AFM data were taken in constant height mode at 0.0 V bias, using a qPlus sensor (resonance frequency ≈22 kHz, oscillation amplitude: 70 pm to 100 pm). The tungsten tip was prepared by focused-ion beam processing and in situ tip forming. For subsequent tip functionalization, carbon monoxide (CO) was dosed on the cold sample and transferred to the tip. The images were processed using the Gwyddion software package^47^.

### Computational details

DFT Calculations in Fig. 4 were performed using the Gaussian 09 software package. The geometries were optimized at the B3LYP/6-311 G(d,p) level. The excitation energies were calculated by the TDDFT method at the B3LYP/6-311 G(d,p) level. NICS were calculated using the gauge invariant atomic orbital (GIAO) approach at the GIAO-B3LYP/6-311 + G(2d,p) level. NICS(1) values were averaged by two positions (above and below the plane). For Figs. 5 and 6, periodic DFT calculations were performed with the VASP code^48^, projector augmented wave potentials^49^ and plane waves expanded to a kinetic energy cutoff of 400 eV. Exchange-correlation effects were treated by the van der Waals density functional^50^ in the version denoted as rev-vdWDF2^51^. The Ag(111) surface was represented by four layered slab separated by approx. 15 Å of vacuum region. Furthermore, a *p*(10×10) super cell was used together with a 2×2 *k*-point sampling. All structures were geometrically optimized until the residual forces on all atoms were smaller than 0.01 eV/Å, except for the bottom two layers of the slab, which were frozen in the bulk distance. An exhaustive conformational search identified the preferred adsorption site (Site b, Supplementary Fig. 4). The site’s adsorption height was further optimized for a gas-phase molecule and found to be 3.22 Å between the molecular and metallic planes (Site c, Supplementary Fig. 4). This site was employed to simulate the STM and AFM images. STM images were simulated with the Tersoff-Hamann approximation^52^ using the implementation by Lorente and Persson^53^. AFM simulations were performed with a modification of the particle probe model code^42^ by Hapala et al. The DFT-derived Hartree potential was employed to prepare an electrostatic grid which together with the molecular Lennard-Jones potential acted as the force-field for simulating the frequency shift of a probe C = O particle as detailed in ref. ^46^. The simulations involved oscillating a probe particle with an amplitude of 5 Å at a height of 7.7 Å above the substrate. The terminal oxygen was given a negative charge of - 0.005*e*. The probe’s stiffness *x,y,z* components were set to 3 N m^−1^, 3 N m^−1^ and 50 N m^−1^, respectively.

### Data availability

X-Ray crystallographic data can be obtained free of charge from the Cambridge Crystallographic Data Centre (CCDC) with the CCDC number 1529624 via its website (https://www.ccdc.cam.ac.uk/structures/). All other data are available within the article and its Supplementary Information or from the corresponding authors upon reasonable request.

## Electronic supplementary material

Supplementary Information
