# Supplementary material for: Exploration of pyrazine-embedded antiaromatic polycyclic hydrocarbons generated by solution and on-surface azomethine ylide homocoupling
Source: Nat Commun. 2017 Dec 5;8:1948. doi: 10.1038/s41467-017-01934-1 (PMC5717246; doi:10.1038/s41467-017-01934-1)
Supplement: Supplementary file 1 — Supplementary Information [file 41467_2017_1934_MOESM1_ESM.pdf]

Supplementary Methods

General Methods. Unless otherwise noted, the commercially available reagents and anhydrous solvents were used without further purification. For microwave assisted reactions a CEM Discover-SP w/activent 909155 was used. Column chromatography was done with silica gel (particle size 0.063-0.200mm from Macherey-Nagel) and silica-gel-coated aluminum sheets with fluorescence indicator from Macherey-Nagel were used for thin layer chromatography. The ^{1}H and ^{13}C NMR spectra were recorded on a Bruker AVANCE 300, Bruker AVANCE 500, Bruker AVANCE 600 or Bruker AVANCE 700 spectrometer. Chemical shifts were reported in ppm. Coupling constants (*J*) were reported in Hertz (Hz). ^{1}H NMR chemical shifts were referenced to C_2HDCl_4 (6.00 ppm) or DMSO-d_5 (2.50 ppm). ^{13}C NMR chemical shifts were referenced to $C_2D_2Cl_4$ (73.78 ppm) or DMSO-d_6 (39.52 ppm). In some cases, the attached proton test (APT) technique of ^{13}C NMR (J-modulated spin-echo for ^{13}C-nuclei coupled to ^{1}H-nuclei) was used to distinguish the carbon atoms with even or odd number of attached protons. The quaternary (C) and methylene (CH_2) signals were in the upper phase and the methine (CH) and methyl (CH_3) signals were in the opposite phase. Two dimensional (2D) NMR spectroscopy, including ^{1}H-^{1}H correlation spectroscopy (COSY), ^{1}H-^{1}H nuclear Overhauser enhancement spectroscopy (NOESY), ^{1}H-^{13}C heteronuclear single-quantum correlation (HSQC) spectroscopy, and ^{1}H-^{13}C heteronuclear multiple-bond correlation (HMBC) spectroscopy were measured on a Bruker AVANCE 500, Bruker AVANCE 600, or Bruker AVANCE 700 spectrometer. High-resolution mass spectrometry (HRMS) was performed on a Q-Tof Ultima 3 (micromass/Waters) by electrospray ionization (ESI), or on a Bruker Reflex II-TOF spectrometer using a 337 nm nitrogen laser by matrix-assisted laser desorption/ionization (MALDI) with/without trans-2-[3-(4-*tert*-Butylphenyl)-2-methyl-2-propenylidene]malononitrile (DCTB) as the matrix. UV-vis absorption spectra were recorded on a Perkin-Elmer Lambda 900 spectrophotometer and photoluminescence spectra were recorded on a J&MTIDAS spectrofluorometer.

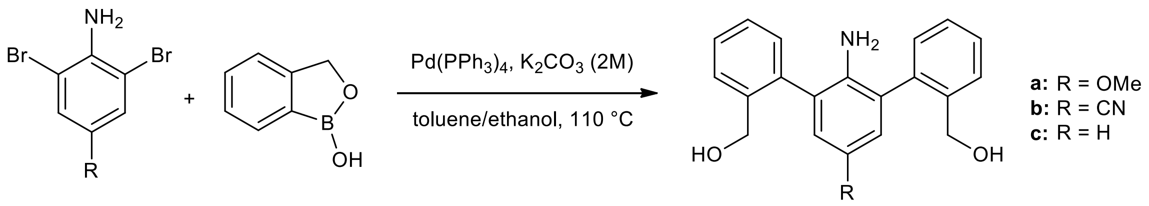

9 **10** **11**

General procedure for the synthesis of 11. A solution of **9** (13.0 mmol, 1.00 eq.) and 1-hydroxy-3*H*-2,1-benzoxaborole (5.22 g, 39.0 mmol, 3.00 eq.) in a mixture of toluene (320 mL), ethanol (60 mL) and 2.0 M K_2CO_3 aqueous solution (120 mL) was bubbled with argon for 30 min. After tetrakis(triphenylphosphine)palladium (1.35 g, 1.17 mmol, 3 mol%) was added, the mixture was refluxed in an oil bath at 110 °C overnight. The reaction mixture was allowed to cool down to room

temperature and the organic layer was separated. The aqueous phase was extracted with diethyl ether (100 mL, three times) and the combined organic layers were washed with brine and dried over $MgSO_4$. The solvent was removed under reduced pressure and the residue was purified by column chromatography over silica gel (eluent: hexane/ethyl acetate = 3 : 2) to afford the title compounds **11a-c** as pale grey solids. The analytical data of **11a** were reported previously.[1]

11b (Yield: 81%). ^{1}H NMR (300 MHz, DMSO-d_6, 298 K, ppm) δ 7.63 (d, J = 7.8 Hz, 2H), 7.45 (t, J = 7.5 Hz, 2H), 7.41 – 7.29 (m, 4H), 7.19 (d, J = 7.5 Hz, 2H), 5.19 – 5.05 (m, 2H), 4.50 (br, 2H), 4.40 – 4.22 (m, 4H). ^{13}C NMR (75 MHz, DMSO-d_6, 298 K, ppm) δ 146.59, 140.85, 140.69, 134.73, 134.55, 133.04, 129.76, 129.67, 128.28, 127.64, 127.35, 127.32, 125.45, 120.04, 97.32, 97.22, 60.41, 60.23. Hydrogen bonds between amino and hydroxyl substituents hinder the free rotation of the phenyl substituents, resulting in isomers with complex ^{1}H and ^{13}C NMR spectra.[1] HRMS (ESI) m/z: Calcd for $C_{21}H_{18}N_2O_2Na$: 353.1266; found: 353.1259 $[M + Na]^+$.

11c (Yield: 80%).^{1}H NMR (300 MHz, DMSO-d_6, 298 K, ppm) δ 7.61 (d, J = 7.6 Hz, 2H), 7.46 – 7.27 (m, 4H), 7.21 – 7.11 (m, 2H), 6.91 (d, J = 7.4 Hz, 2H), 6.81 – 6.69 (m, 1H), 5.17 – 5.02 (m, 2H), 4.47 – 4.22 (m, 4H), 3.69 – 3.56 (m, 2H). ^{13}C NMR (150 MHz, DMSO-d_6, 298 K, ppm) δ 141.49, 141.44, 140.91, 140.66, 136.74, 136.56, 129.71, 129.54, 129.10, 129.05, 127.54, 127.52, 127.10, 126.95, 126.93, 126.71, 125.55, 125.53, 116.83, 116.68, 60.48, 60.28. Hydrogen bonds between amino and hydroxyl substituents hinder the free rotation of the phenyl substituents, resulting in isomers with complex ^{1}H and ^{13}C NMR spectra.[1] HRMS (ESI) m/z: Calcd for $C_{20}H_{20}NO_2$: 306.1494; found: 306.1491 $[M + H]^+$.

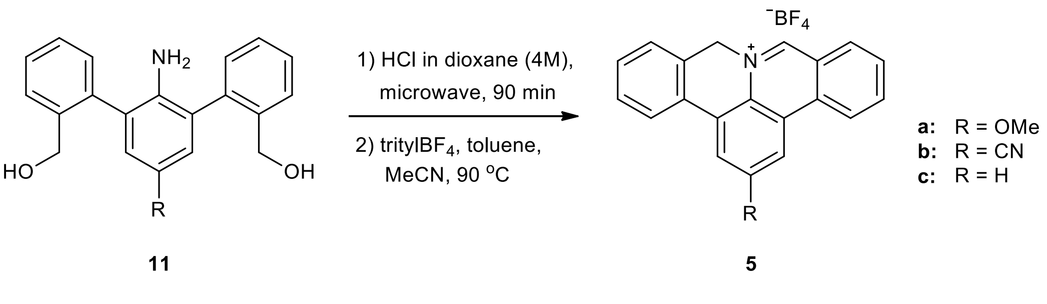

General procedure for the synthesis of 5. To a microwave tube was added compound **11** (2.76 mmol, 1.00 eq) and an anhydrous hydrogen chloride solution (4.0 M in dioxane, 10 mL). The tube was then capped and placed in a microwave reactor. A dynamic mode was chosen (300 W, power max: on, activated cooling, pre-stirring: 10 s, temperature: 130 °C) for 90 min. After cooling to room temperature, the solvent was removed under reduced pressure. The crude product was dissolved in toluene (anhydrous, 20 mL) and heated to 90 °C under argon. Then a solution of triphenylcarbenium tetrafluoroborate (1.00 g, 3.04 mmol, 1.10 eq.) in anhydrous acetonitrile (10 mL) was added dropwise. The mixture was stirred for 30 min and the solvents were removed under reduced pressure. The residue was dissolved in a minimum amount of dichloromethane (~20 mL) and precipitated in hexane (250 mL). The crude product was washed with a mixture of hexane and DCM

(100 mL, 9:1) and precipitated again to obtain the title compounds **5a-c** as yellow solids. The analytical data of **5a** were reported previously.[1] Compound **5b** was further purified by precipitation from DMSO/THF in a freezer at −20 °C.

5b (Yield: 78%). ^{1}H NMR (300 MHz, DMSO-d_6, 298 K, ppm) δ 10.40 (s, 1H), 9.70 (s, 1H), 9.24 (d, J = 8.4 Hz, 1H), 9.18 (s, 1H), 8.70 (d, J = 8.0 Hz, 1H), 8.50 (t, J = 7.6 Hz, 1H), 8.41 (t, J = 4.5 Hz, 1H), 8.22 (t, J = 7.6 Hz, 1H), 7.71 – 7.57 (m, 3H), 6.26 (s, 2H). ^{13}C NMR (75 MHz, DMSO-d_6, 298 K, ppm) δ 156.96, 138.91, 133.48, 132.98, 131.60, 130.89, 129.42, 129.26, 128.48, 128.12, 127.17, 126.61, 126.44, 125.73, 124.68, 124.25, 123.92, 117.72, 113.52, 57.14. HRMS (ESI) *m/z*: Calcd for $C_{21}H_{13}N_2$: 293.1079; found 293.1078 $[M - BF_4]^+$.

5c (Yield: 92%). ^{1}H NMR (300 MHz, DMSO-d_6, 298 K, ppm) δ 10.25 (s, 1H), 9.15 (d, J = 8.5 Hz, 1H), 9.10 (d, J = 8.3 Hz, 1H), 8.78 (d, J = 7.7 Hz, 1H), 8.64 (d, J = 7.7 Hz, 1H), 8.43 (ddd, J = 8.3, 7.0, 1.3 Hz, 1H), 8.35 – 8.25 (m, 1H), 8.22 – 8.09 (m, 2H), 7.68 – 7.52 (m, 3H), 6.25 (s, 2H). ^{13}C NMR (150 MHz, DMSO-d_6, 298 K, ppm) δ 154.24, 138.02, 134.03, 132.48, 130.72, 130.62, 129.94, 129.71, 129.24, 128.19, 126.90, 126.47, 126.43, 126.12, 125.52, 124.18, 124.00, 123.87, 123.44, 57.06. HRMS (ESI) *m/z*: Calcd for $C_{20}H_{14}N$: 268.1126; found 268.1118 $[M - BF_4]^+$.

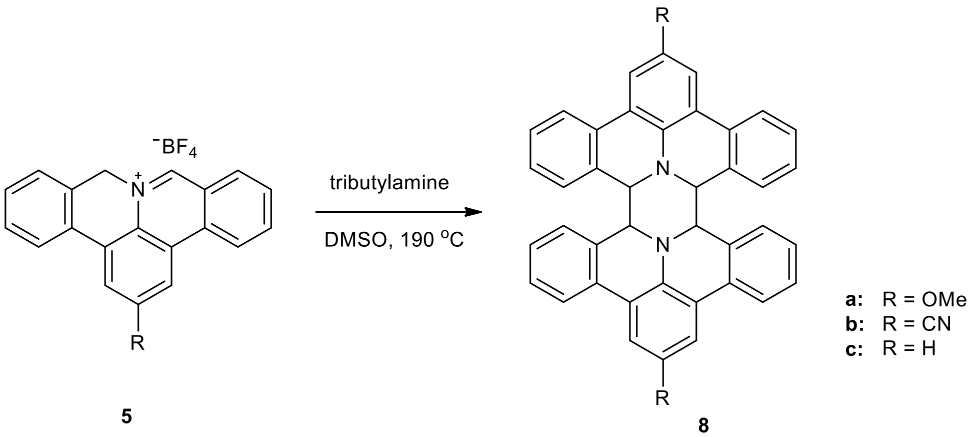

5 **8**

General procedure for synthesis of 8. In a dry and inert 25-mL Schlenk tube compound **5** (0.560 mmol, 1.00 eq.) was dissolved in dimethyl sulfoxide (anhydrous, Ar bubbled, 3.0 mL) and heated to 190 °C. Tributylamine (anhydrous, Ar bubbled, 872 mg, 3.36 mmol, 6.00 eq.) was added in one shot and the reaction mixture was stirred for additional five minutes. After cooling to room temperature, ethanol (15 mL) was injected and the Schlenk tube was cooled in a refrigerator overnight. The precipitate was filtered and washed with ethanol to afford the title compounds **8a-c** as yellow solids.

8a (Yield: 51%). ^{1}H NMR (600 MHz, $C_2D_2Cl_4$, 298 K, ppm) δ 7.62 (d, J = 7.4 Hz, 4H), 7.40 (s, 4H), 7.26 (td, J = 7.6, 1.3 Hz, 4H), 6.86 (td, J = 7.4, 1.1 Hz, 4H), 6.04 (dd, J = 7.6, 1.3 Hz, 4H), 5.12 (s, 4H), 4.00 (s, 6H). ^{13}C NMR (150 MHz, $C_2D_2Cl_4$, 298 K, ppm) δ 152.94, 134.76, 130.32, 129.56, 128.58, 128.12, 126.77, 123.63, 121.81, 109.14, 60.41, 55.86. HRMS (ESI) *m/z*: Calcd for $C_{42}H_{29}N_2O_2$: 593.2229; found 593.2226 $[M - H]^+$.

8b (Yield: 39%). ^{1}H NMR (500 MHz, $C_2D_2Cl_4$, 413 K, ppm) δ 8.06 (s, 4H), 7.72 (d, J = 7.9 Hz, 4H), 7.39 (t, J = 7.6 Hz, 4H), 7.00 (t, J = 7.5 Hz, 4H), 6.12 (d, J = 7.6 Hz, 4H), 5.18 (s, 4H). ^{13}C NMR (125 MHz, $C_2D_2Cl_4$, 413 K, ppm) δ 128.90, 128.86, 128.75, 127.99, 127.65, 126.52, 124.21, 122.76, 121.91, 63.28. HRMS (ESI) *m/z*: Calcd for $C_{42}H_{23}N_4$: 583.1923; found 583.1898 $[M - H]^{+}$.

8c (Yield: 84%). ^{1}H NMR (700 MHz, $C_2D_2Cl_4$, 323 K, ppm) δ 7.82 (d, J = 7.4 Hz, 4H), 7.66 (d, J = 7.7 Hz, 4H), 7.27 (t, J = 7.4 Hz, 4H), 7.03 (t, J = 7.4 Hz, 2H), 6.85 (t, J = 7.3 Hz, 4H), 6.04 (d, J = 7.3 Hz, 4H), 5.16 (s, 4H). ^{13}C NMR (175 MHz, $C_2D_2Cl_4$, 323 K, ppm) δ 140.31, 130.47, 128.88, 128.59, 128.15, 126.52, 123.41, 122.41, 121.73, 118.99, 61.17. HRMS (ESI) *m/z*: Calcd for $C_{40}H_{25}N_2$: 533.2018; found 533.2006 $[M - H]^{+}$.

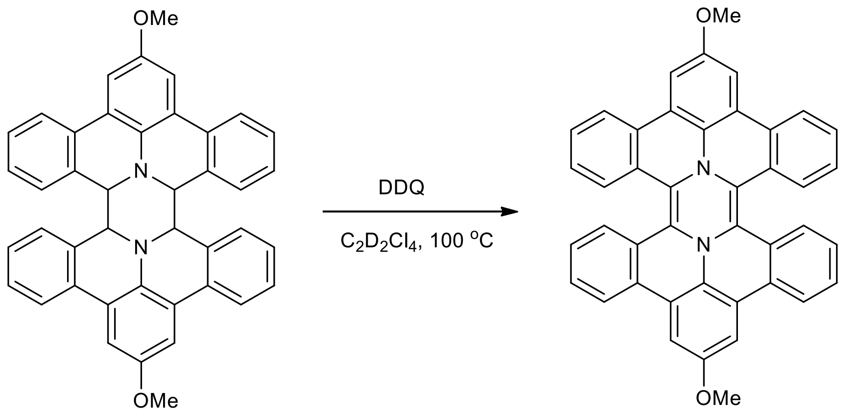

8a **6a**

Procedure for the *in situ* generation of 6a. Inside an argon-filled glovebox, precursor **8a** (2.0 mg, 1.0 eq.), 2,3-dichloro-5,6-dicyano-1,4-benzoquinone (15 mg, 20 eq.) and $C_2D_2Cl_4$ (0.6 mL) were added sequentially into a sealable NMR tube. The mixture was heated at 100 °C for 1 min and cooled down to room temperature, and then analyzed by ^{1}H NMR to observe the clean generation of **6a**. ^{1}H NMR (500 MHz, $C_2D_2Cl_4$, 298 K, ppm) δ 8.39 (d, J = 6.5 Hz, 4H), 8.19 (d, J = 7.6 Hz, 4H), 7.87 (t, J = 7.1 Hz, 4H), 7.81 (s, 4H), 7.68 (t, J = 7.1 Hz, 4H), 4.04 (s, 6H). ^{13}C NMR (150 MHz, $C_2D_2Cl_4$, 373 K, projected from HSQC and HMBC measurements, ppm) δ 155.5, 132.9, 132.6, 128.9, 128.1, 126.4, 125.9, 121.2, 119.9, 109.4, 55.2. HRMS (MALDI) *m/z*: Calcd for $C_{42}H_{26}N_2O_2$: 590.1994; found 590.1980 $[M]^{+}$. UV-vis absorption ($C_2H_2Cl_4$): λ_{max} (nm) 382, 544, 587.

X-Ray Crystallography. Single crystals of compound **8c** (CCDC number 1529624) were grown by slow evaporation of its solution in tetrachloroethane. A suitable crystal was selected and mounted on a SuperNova, Dual, Cu at zero, AtlasS2 diffractometer. The crystal was kept at 100.0(2) K during data collection. Using Olex2,[2] the structure was solved with the ShelXT[3] structure solution program using Direct Methods and refined with the ShelXL[4] refinement package using Least Squares minimization.

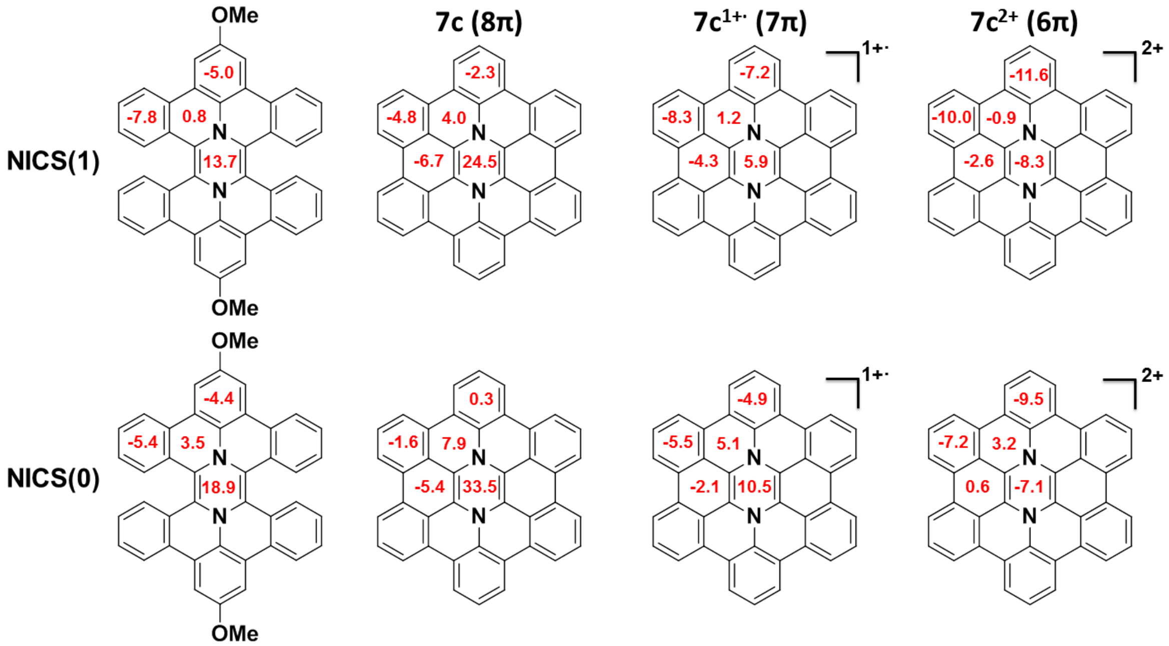

Supplementary Fig. 1 | DFT-calculated NICS values at the center, NICS(0), and 1 Å above the center, NICS(1), on the corresponding rings of compounds **6a** and **7c.** Negative NICS values are indicative of induced diatropic ring currents (aromatic), while positive values imply paratropic ring currents (antiaromatic). Notably, rings adjacent to paratropic currents will experience different NICS values (see Ref. 5). Thus, in **7c** (8π), the central pyrazine ring is highly antiaromatic, which reduces the NICS values of the adjacent benzene rings. In contrast, in **7c**$^{2+}$ (6π), the outer benzenes exhibit NICS values commonly found for aromatic benzenes, due to the oxidized aromatic pyrazine ring. These features indicate that the antiaromatic dopant exerts a significant influence on the whole nanographene molecule. Unit: ppm.

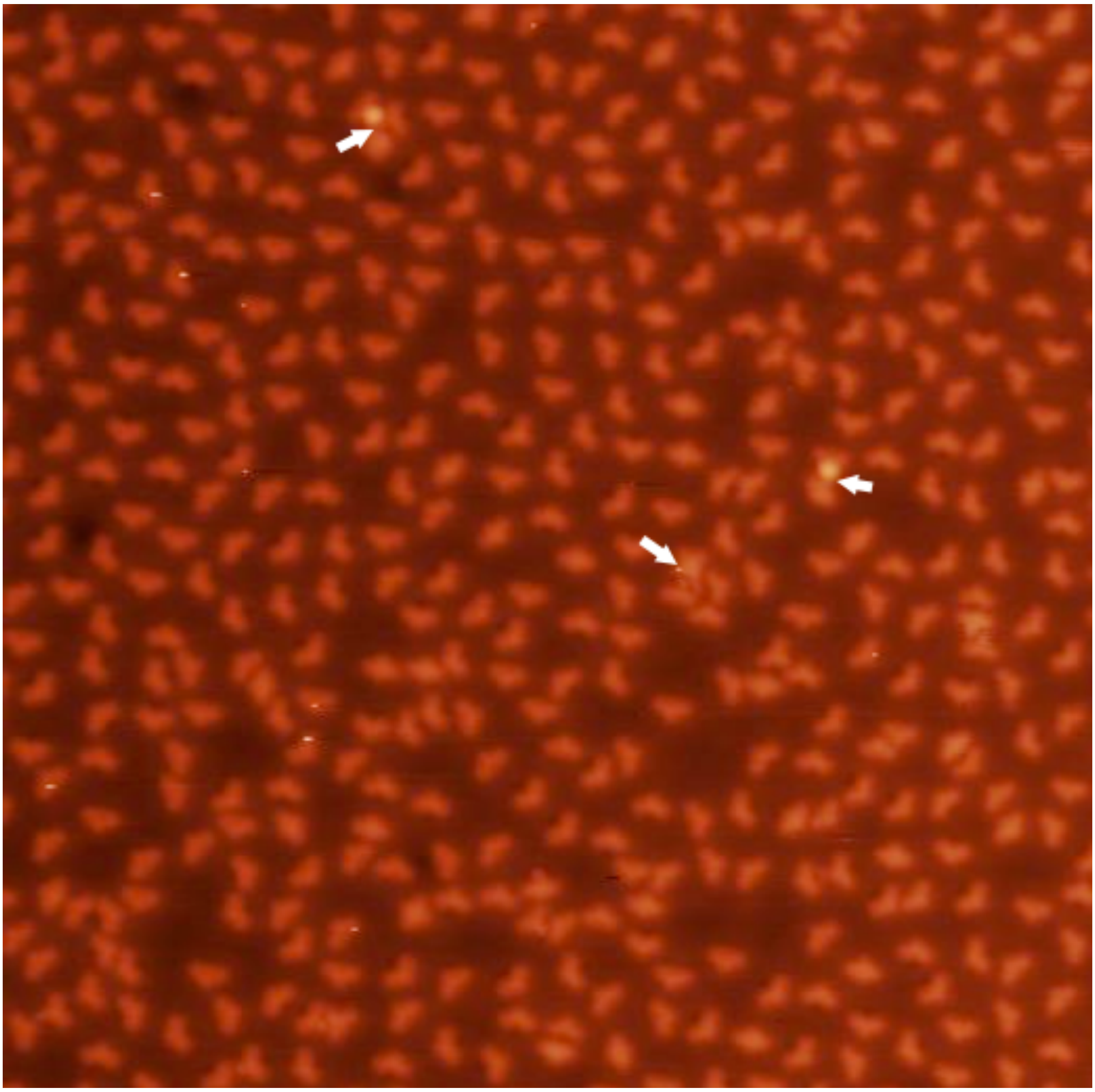

Supplementary Fig. 2 | STM survey (40 x 40 nm^2) data of molecule **5c** on Ag(111). Less than 5% impurities (e.g. white arrows) are encountered (V_s=0.5 V, I_t=10 pA).

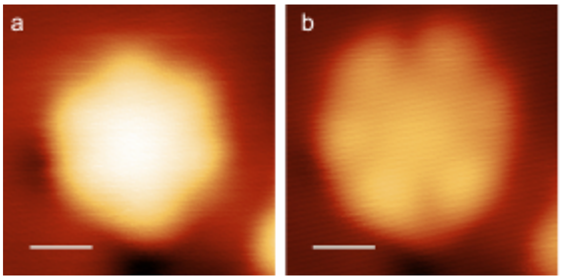

Supplementary Fig. 3 | STM data of *diaza*-HBC **7c** on *h*-BN/Cu(111). **a** I_t=50 pA**,** V_s=2.2 V, **b** I_t=50 pA**,** V_s=2.6 V. Scale bar is 5 Å.

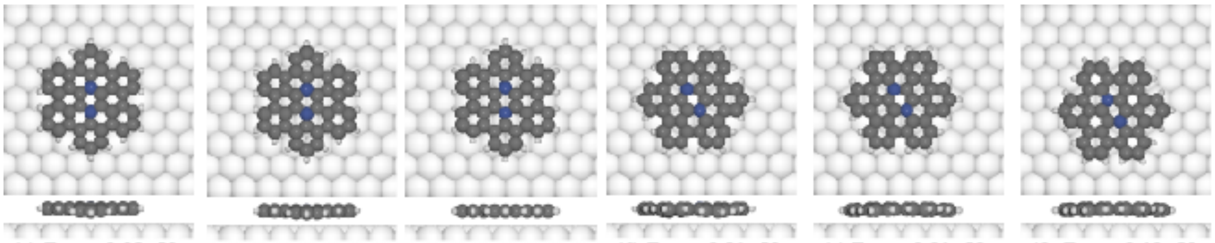

Supplementary Fig. 4 | DFT calculations identify the preferred adsorption site b and state c employed for the STM and AFM simulations (see Methods).

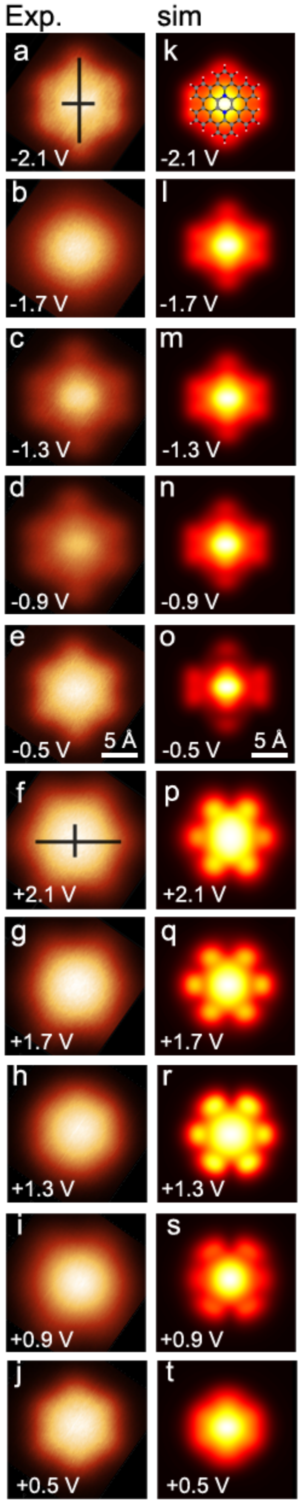

Supplementary Fig. 5 | a-j Experimental constant height STM data for **7c** on Ag(111) and **k-t** simulated data. Data simulated at 9 Å height employing a 0.3 eV broadening.

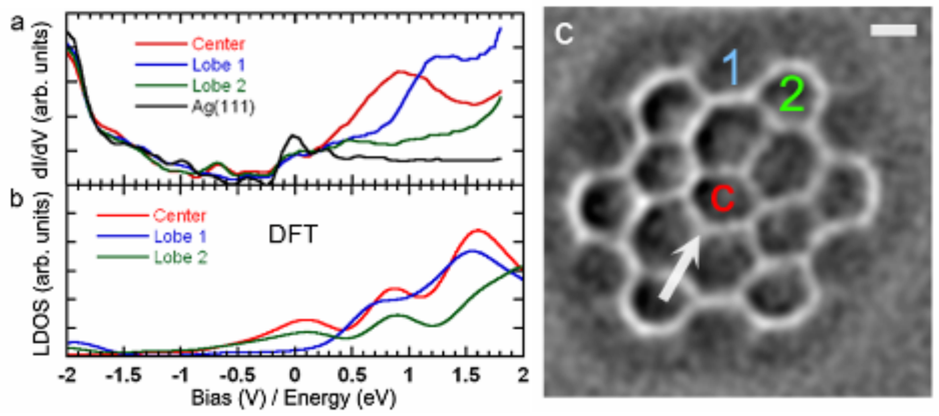

Supplementary Fig. 6 | a Scanning tunneling spectroscopy of **7c** on Ag(111) and **b** simulated local density of states (LDOS) of **7c** on Ag(111) employing a broadening of 0.3 eV. The colors indicate the LDOS measured at different rings as shown in (**c**). Note that unlike the calculated PDOS containing only the p_z projections in main text **Fig. 6**, the LDOS contains all electrons in the molecule, showing a larger contribution of the unoccupied states. Scale bar is 2 Å.

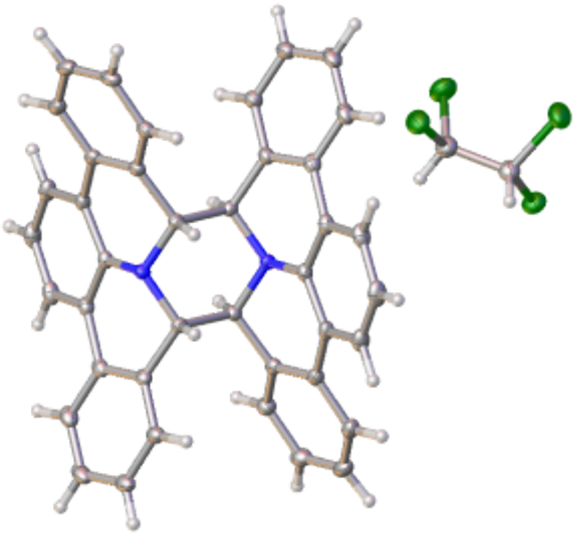

Supplementary Fig. 7 | Single crystal structure of **8c**, together with a solvent molecule in the unit cell.

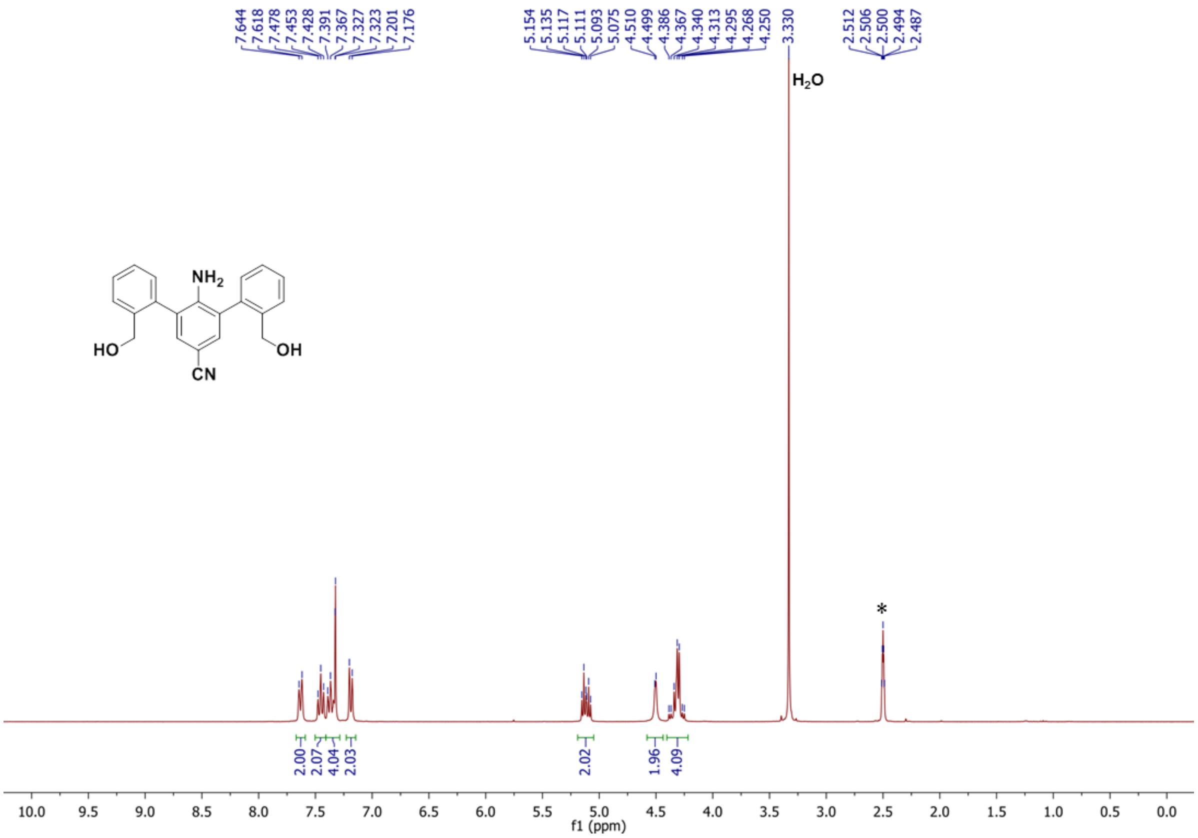

Supplementary Fig. 8 | ^{1}H NMR spectrum of compound **11b** (300 MHz, DMSO-d_6, 298 K). *Deuterated solvent DMSO-d_5.

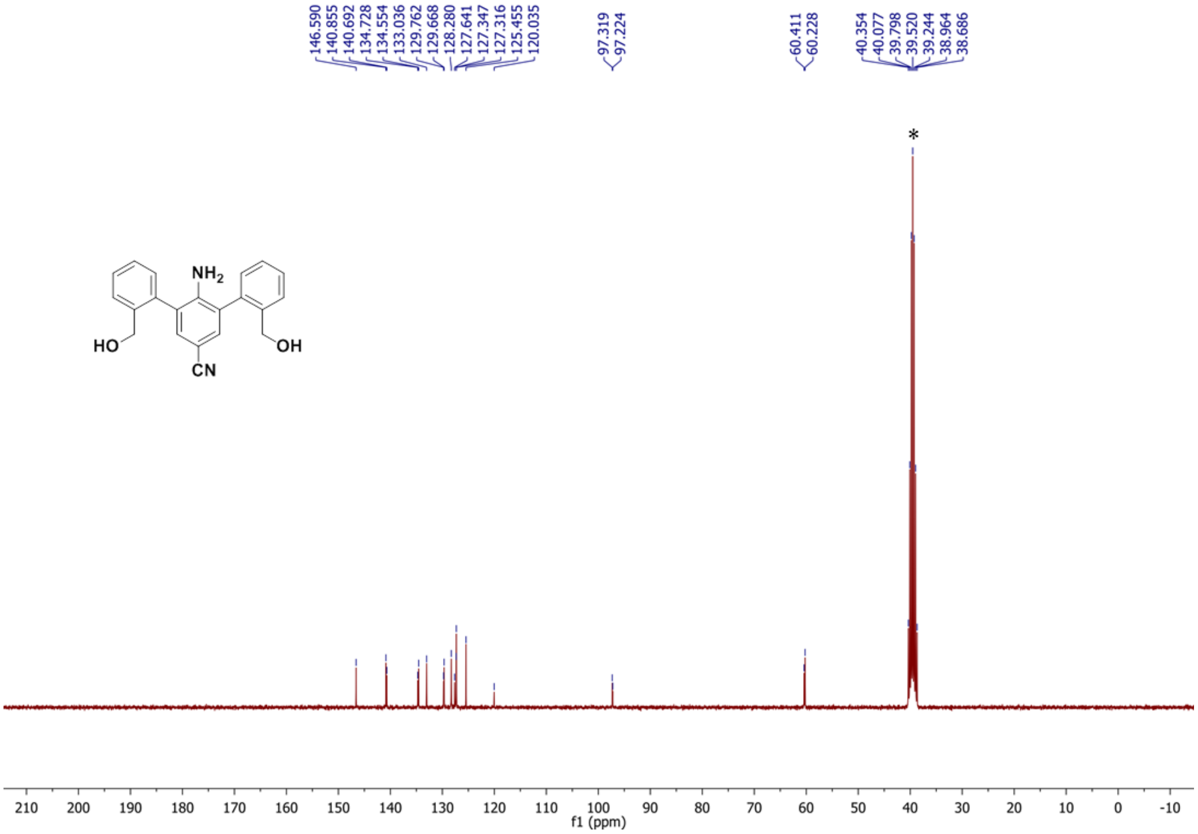

Supplementary Fig. 9 | ^{13}C NMR spectrum of compound **11b** (75 MHz, DMSO-d_6, 298 K). *Deuterated solvent DMSO-d_6.

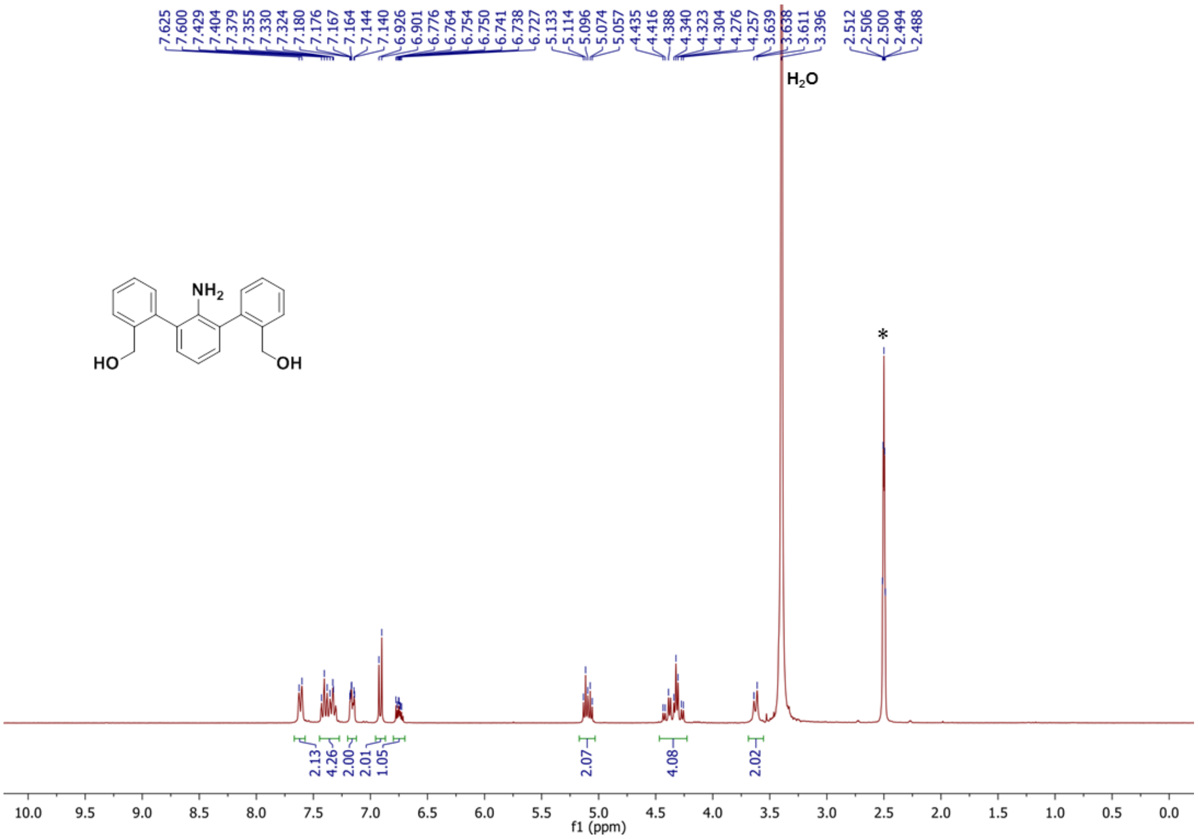

Supplementary Fig. 10 | ^{1}H NMR spectrum of compound **11c** (300 MHz, DMSO-d_6, 298 K).

*Deuterated solvent DMSO-d_5.

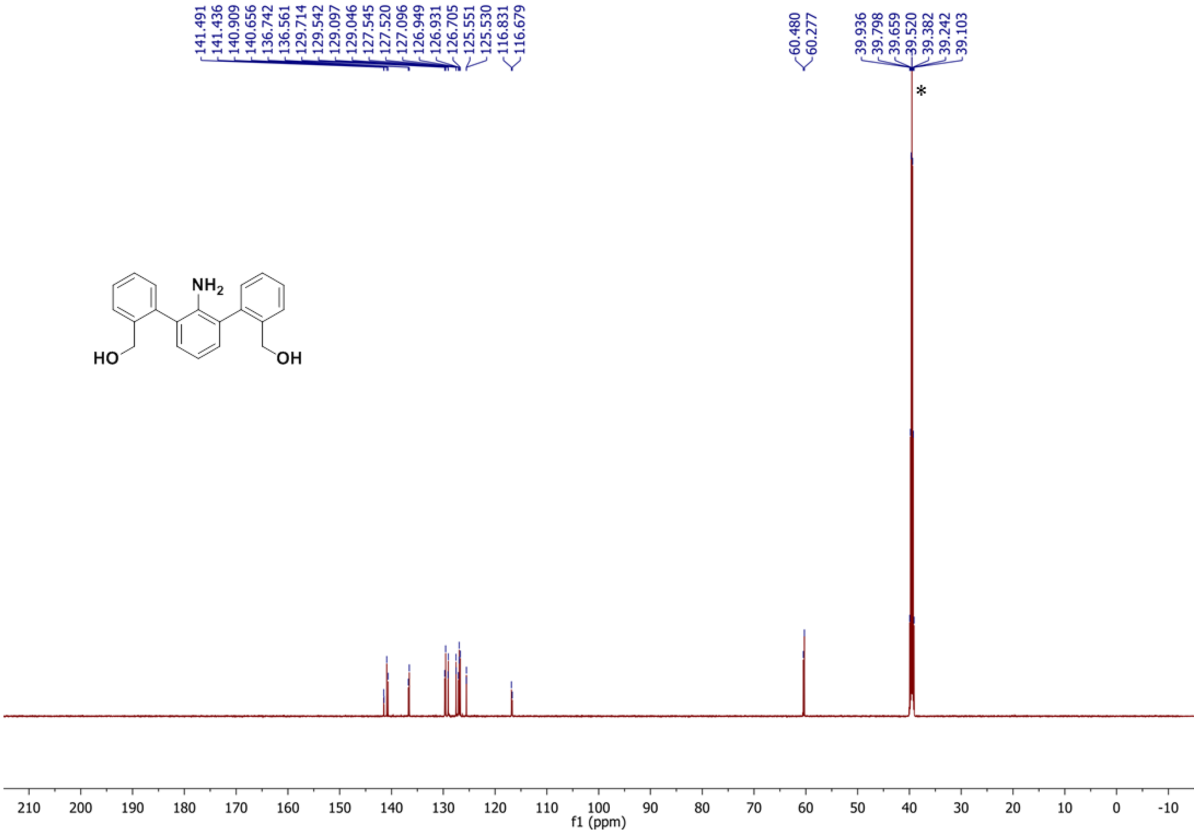

Supplementary Fig. 11 | ^{13}C NMR spectrum of compound **11c** (150 MHz, DMSO-d_6, 298 K).

*Deuterated solvent DMSO-d_6.

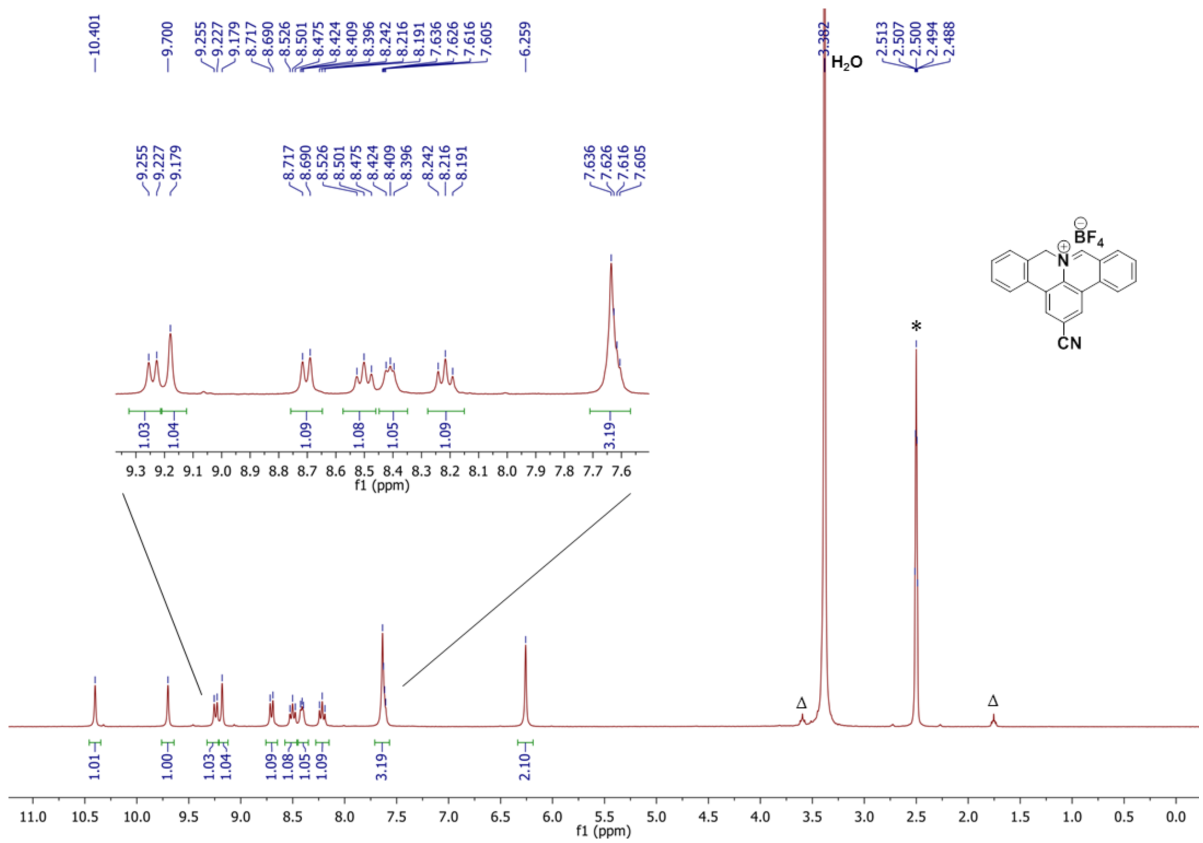

Supplementary Fig. 12| ^{1}H NMR spectrum of compound **5b** (300 MHz, DMSO-d_6, 298 K). *Deuterated solvent DMSO-d_5. $^{\Delta}$Residual solvent THF from the crystallization process.

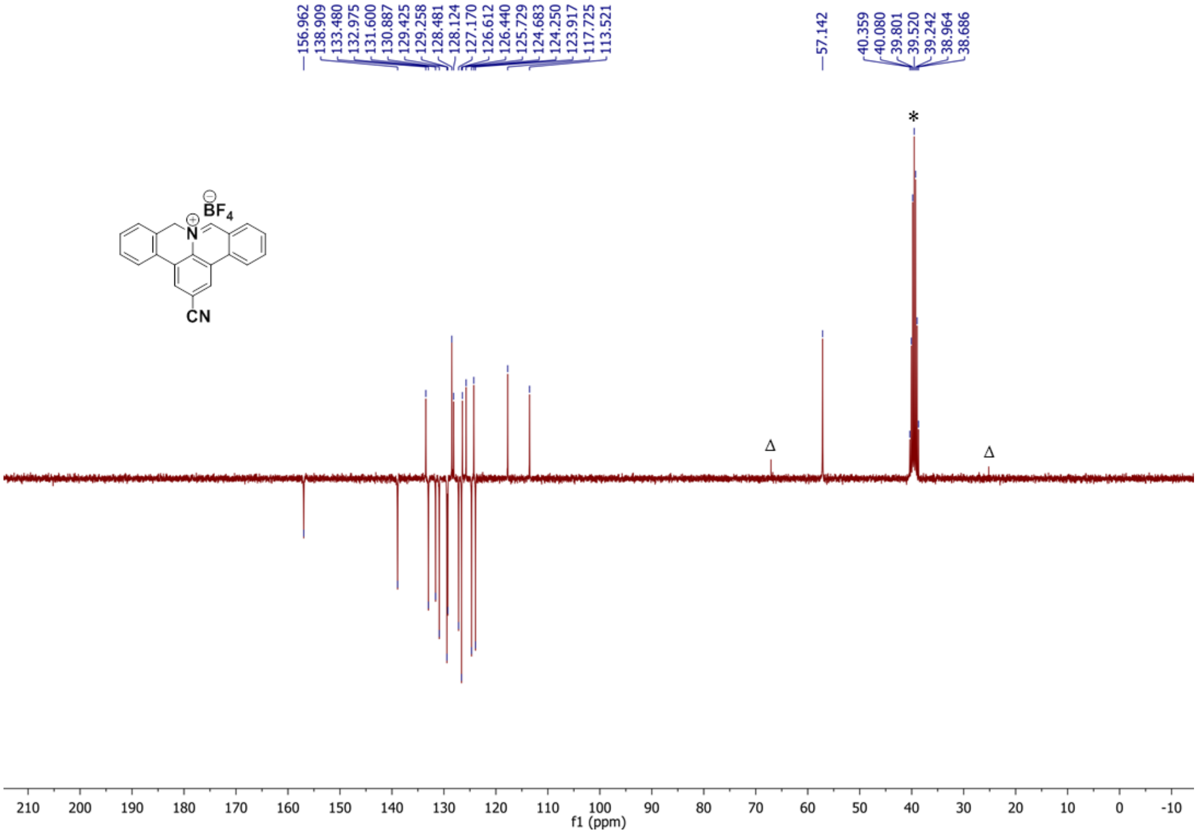

Supplementary Fig. 13 | ^{13}C NMR (APT) spectrum of compound **5b** (75 MHz, DMSO-d_6, 298 K).

*Deuterated solvent DMSO-d_6. $^{\Delta}$Residual solvent THF from the crystallization process.

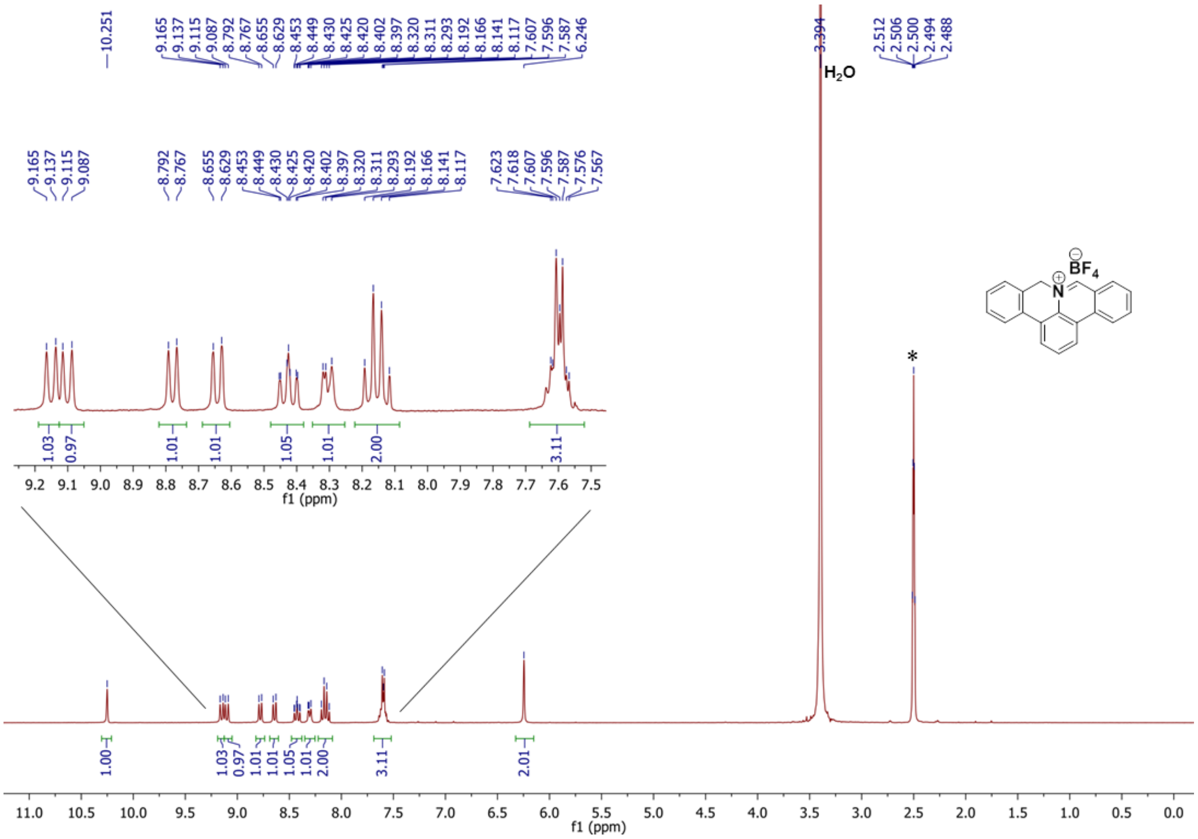

Supplementary Fig. 14 | ^{1}H NMR spectrum of compound **5c** (300 MHz, DMSO-d_6, 298 K).

*Deuterated solvent DMSO-d_5.

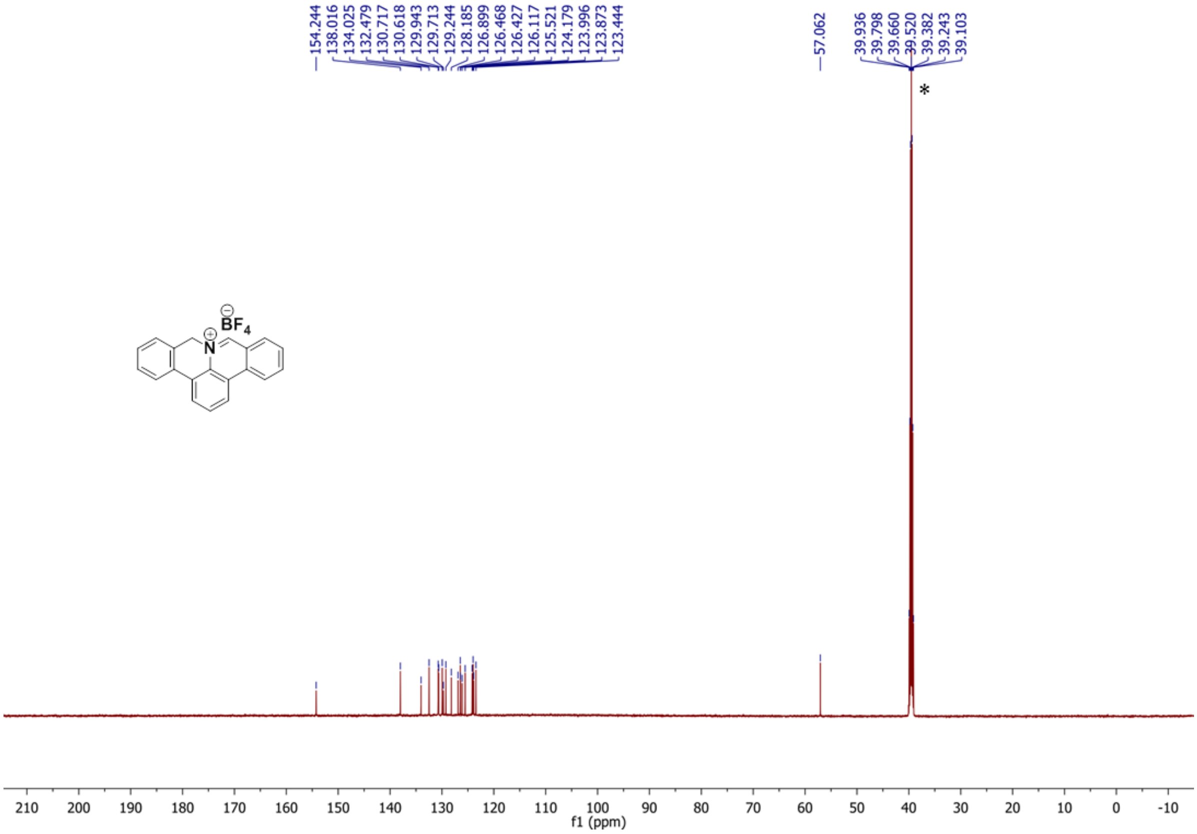

Supplementary Fig. 15 | ^{13}C NMR spectrum of compound **5c** (150 MHz, DMSO-d_6, 298 K).

*Deuterated solvent DMSO-d_6.

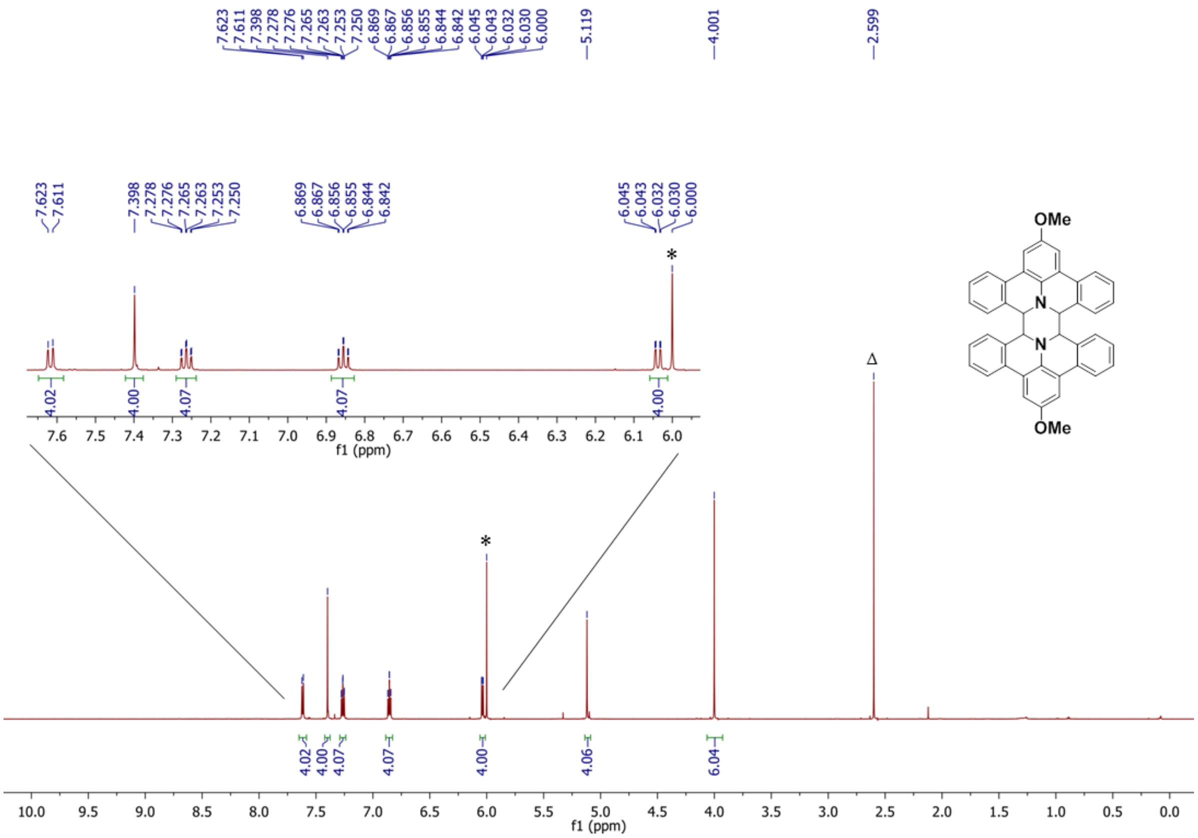

Supplementary Fig. 16 | ^{1}H NMR spectrum of compound **8a** (600 MHz, $C_2D_2Cl_4$, 298 K). *Deuterated solvent C_2HDCl_4. [Δ]Residual solvent DMSO from the reaction.

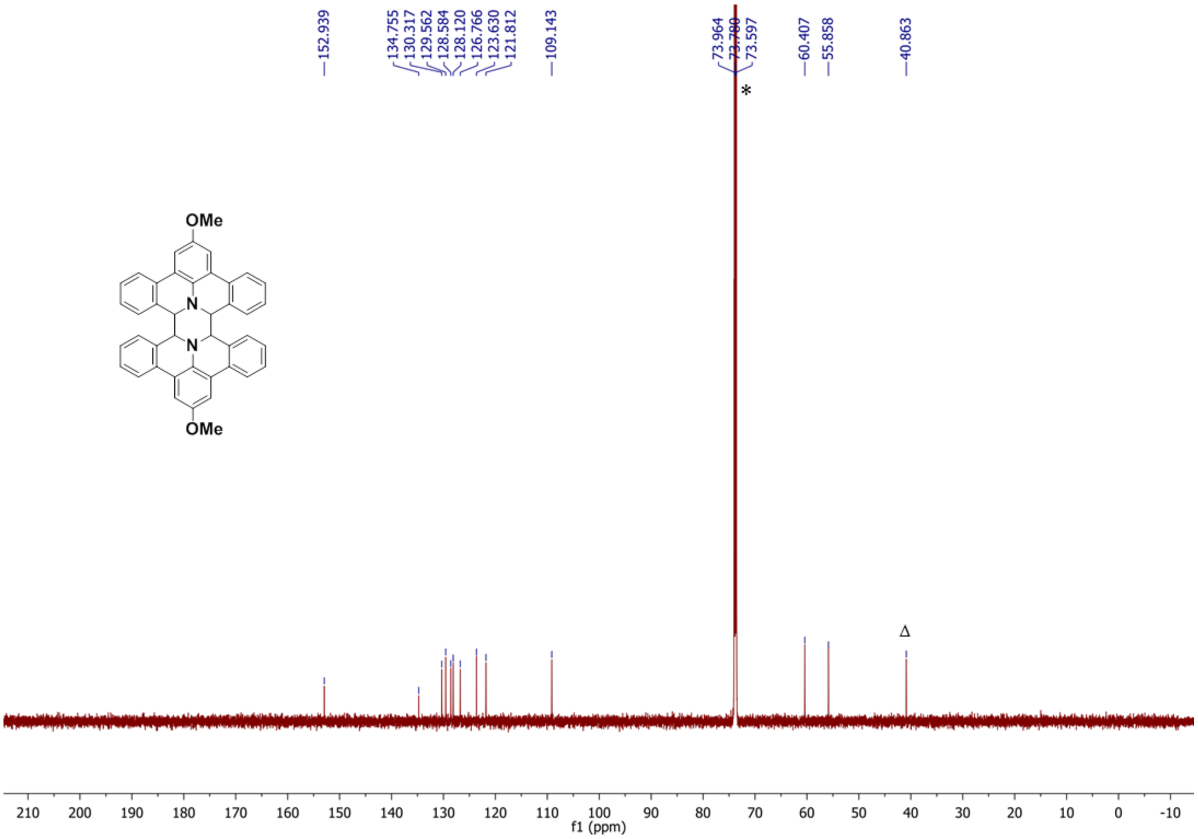

Supplementary Fig. 17 | ^{13}C NMR spectrum of compound **8a** (150 MHz, $C_2D_2Cl_4$, 298 K). *Deuterated solvent $C_2D_2Cl_4$. $^{\Delta}$Residual solvent DMSO from the reaction.

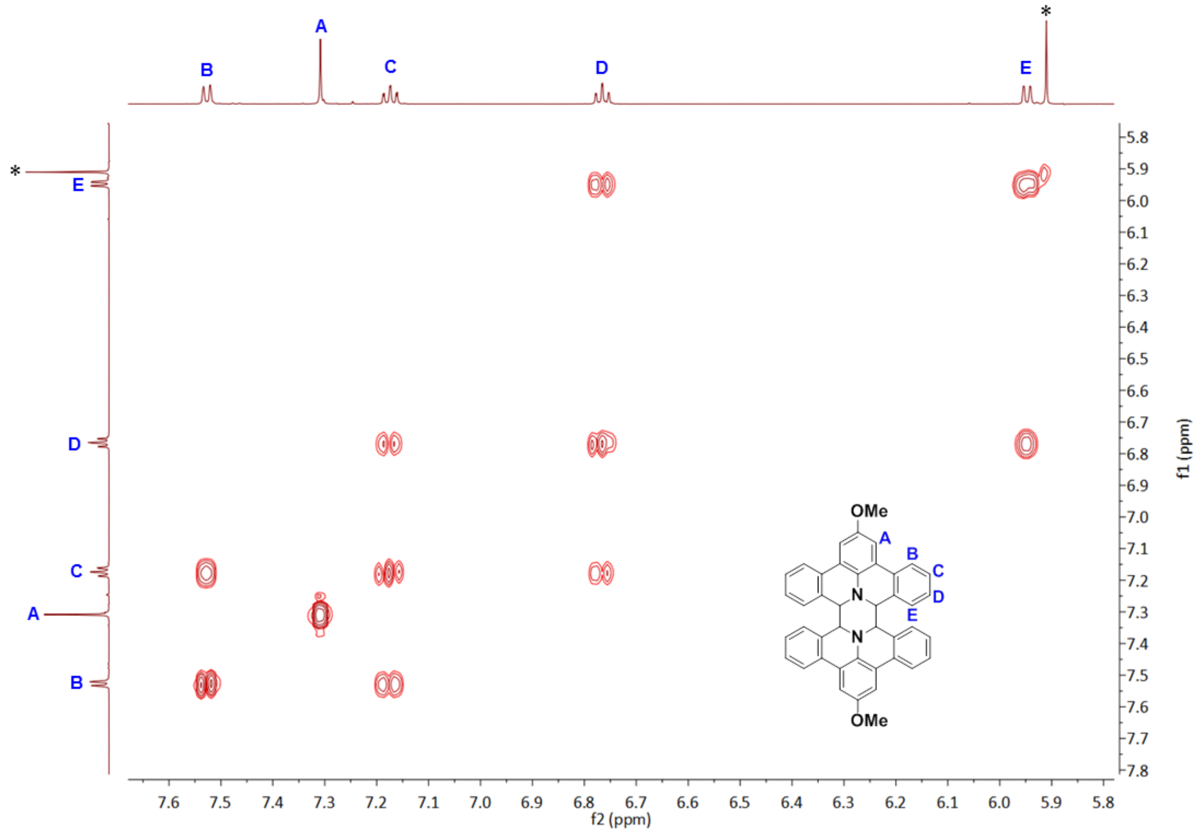

Supplementary Fig. 18 | ^{1}H-^{1}H COSY spectrum of compound **8a** (600 MHz, $C_2D_2Cl_4$, 298 K). *Deuterated solvent C_2HDCl_4.

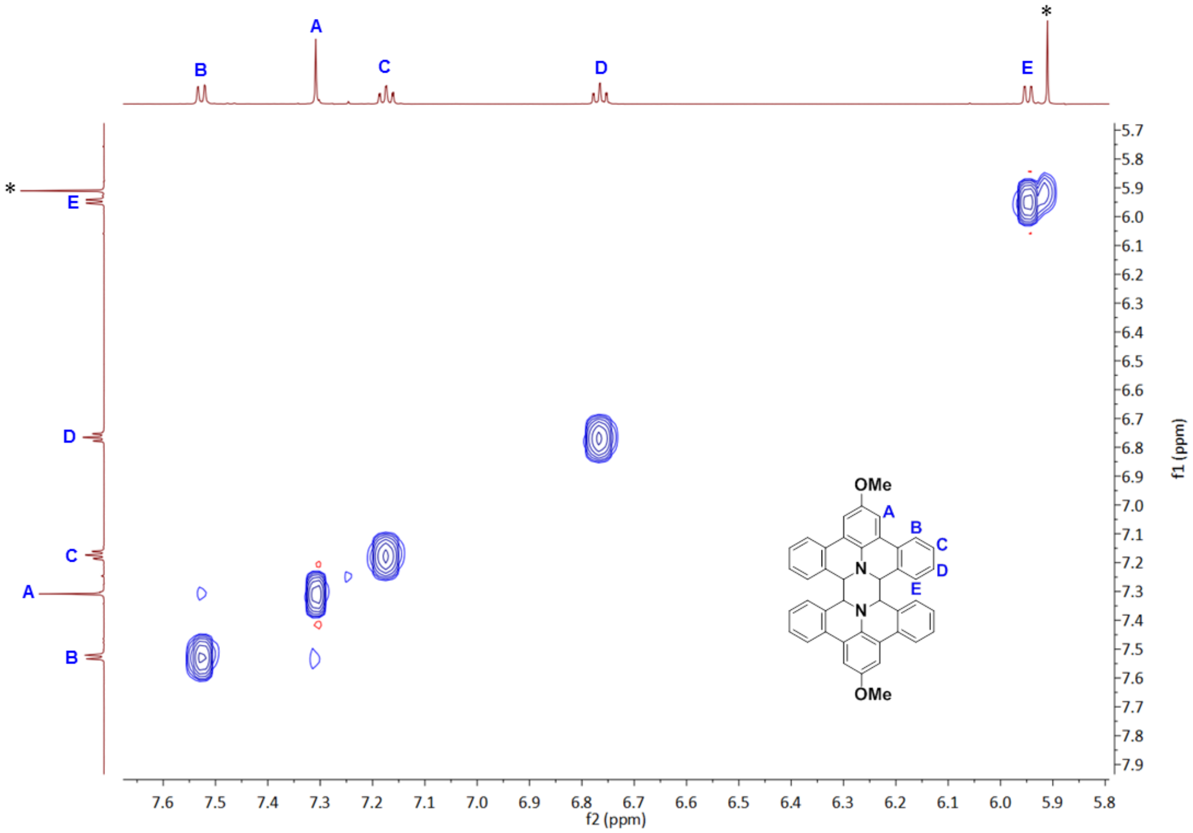

Supplementary Fig. 19 | ^1H-^1H NOESY spectrum of compound **8a** (600 MHz, $C_2D_2Cl_4$, 298 K). *Deuterated solvent C_2HDCl_4.

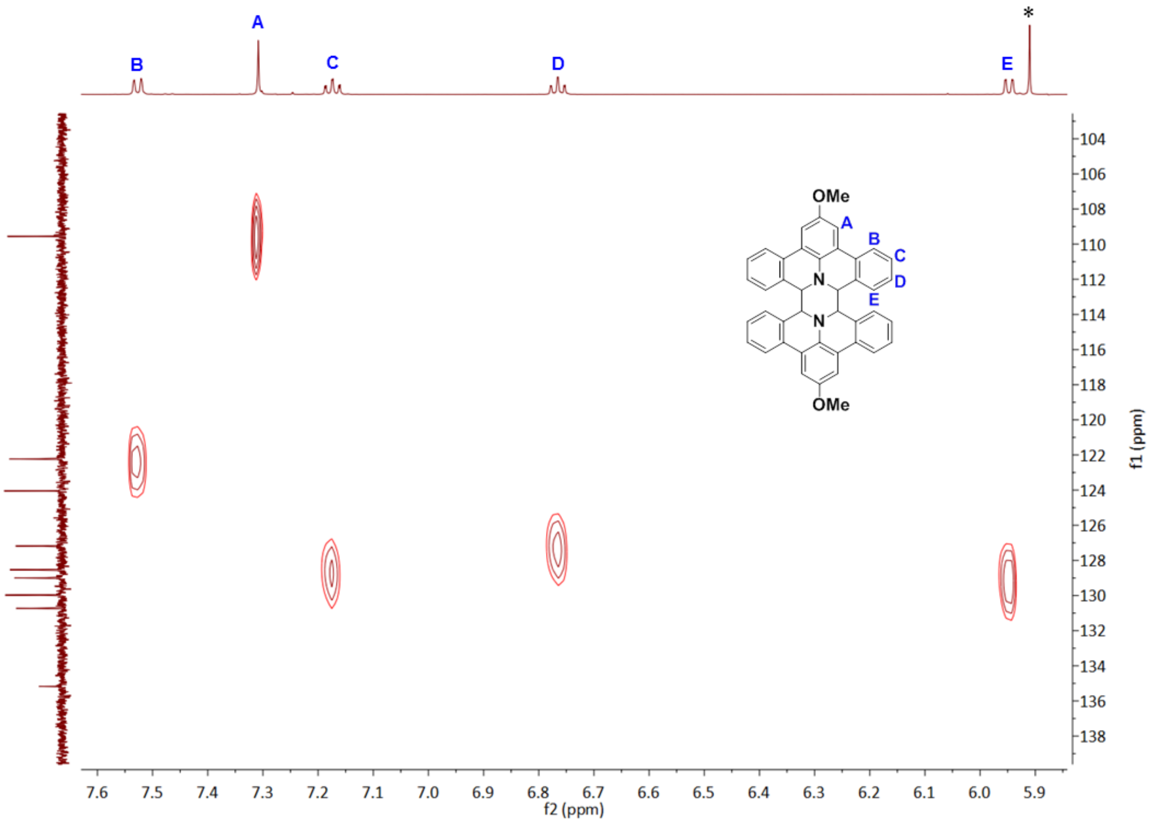

Supplementary Fig. 20 | ^{1}H-^{13}C HSQC spectrum of compound **8a** (600 MHz, $C_2D_2Cl_4$, 298 K). *Deuterated solvent C_2HDCl_4.

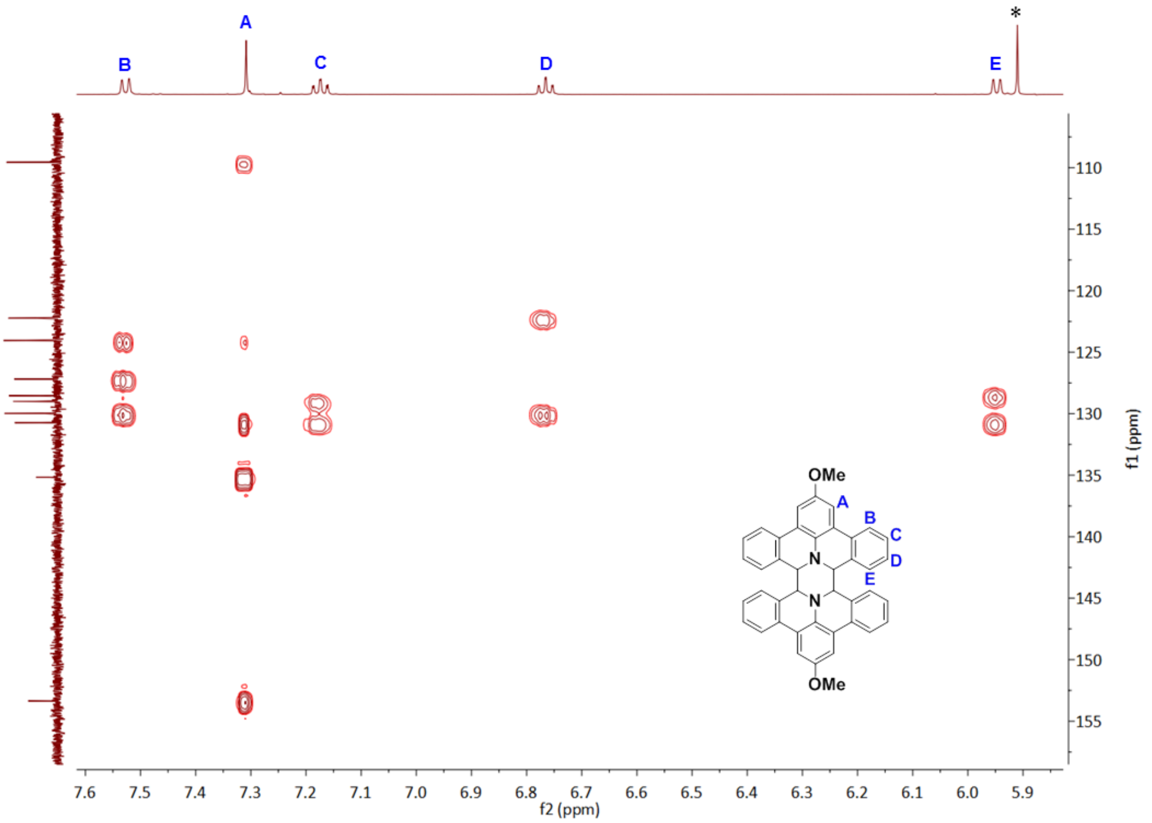

Supplementary Fig. 21 | ^1H-^{13}C HMBC spectrum of compound **8a** (600 MHz, $C_2D_2Cl_4$, 298 K). *Deuterated solvent C_2HDCl_4.

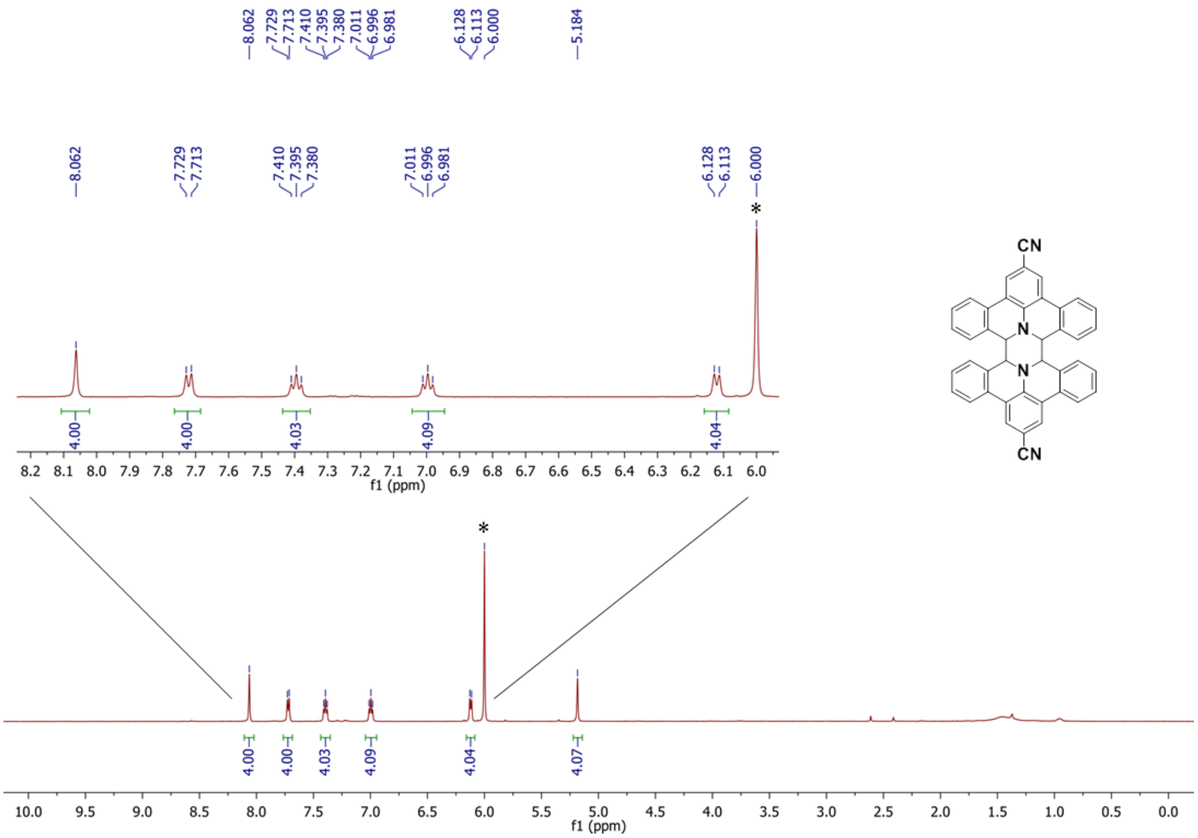

Supplementary Fig. 22 | ^{1}H NMR spectrum of compound **8b** (500 MHz, $C_2D_2Cl_4$, 413 K). *Deuterated solvent C_2HDCl_4.

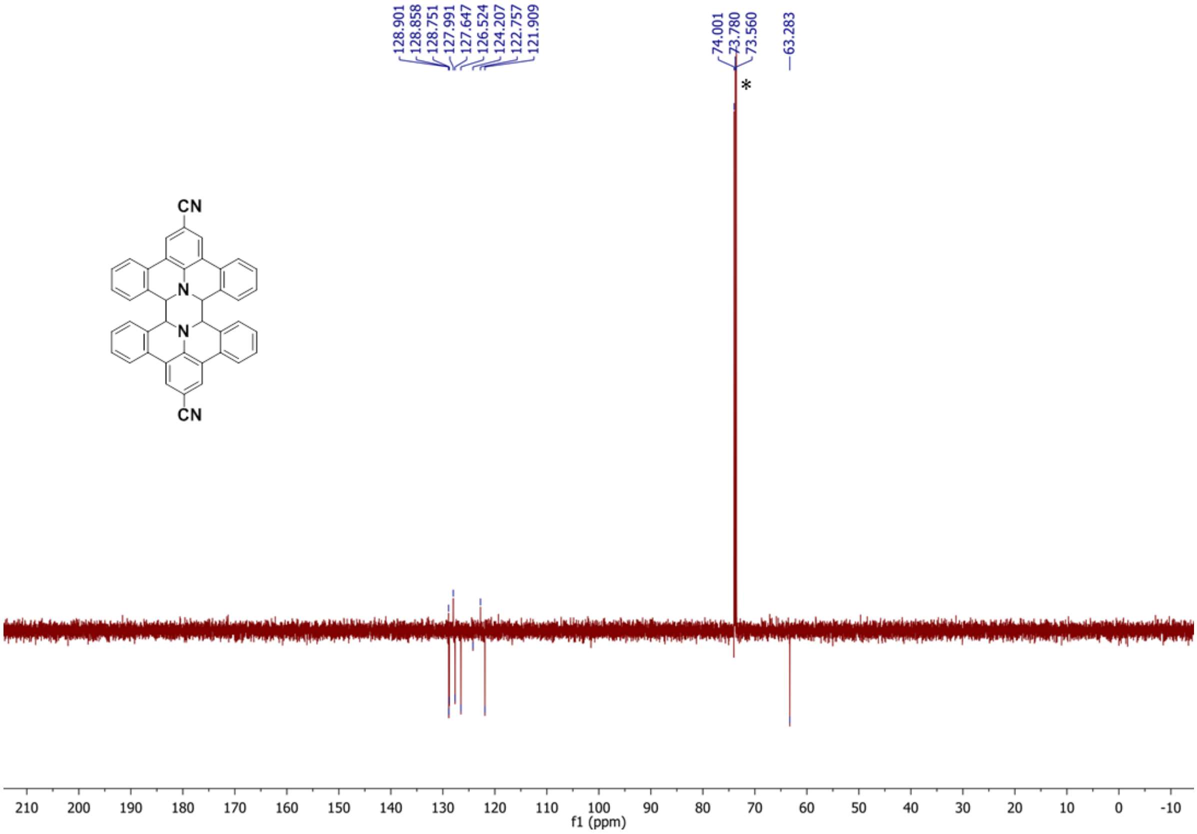

Supplementary Fig. 23 | ^{13}C NMR (APT) spectrum of compound **8b** (125 MHz, $C_2D_2Cl_4$, 413 K). *Deuterated solvent $C_2D_2Cl_4$.

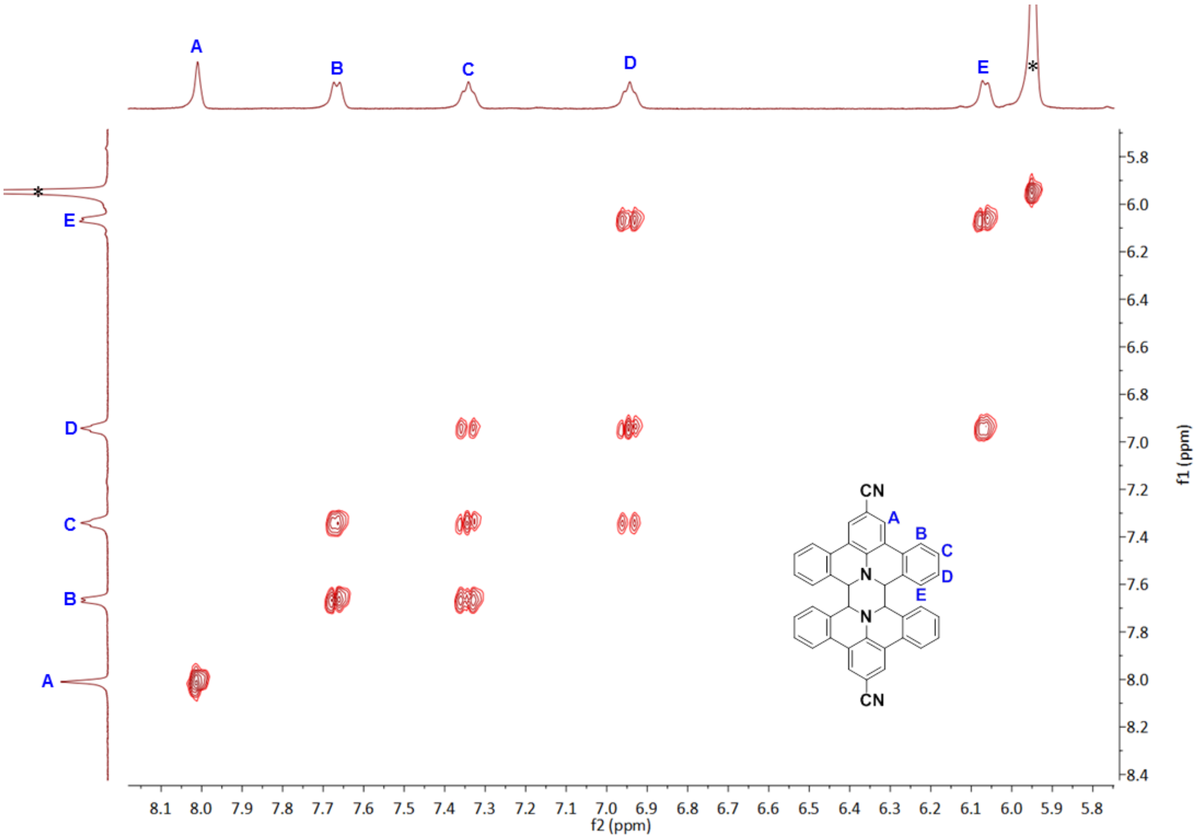

Supplementary Fig. 24 | ^{1}H-^{1}H COSY spectrum of compound **8b** (500 MHz, $C_2D_2Cl_4$, 413 K). *Deuterated solvent C_2HDCl_4.

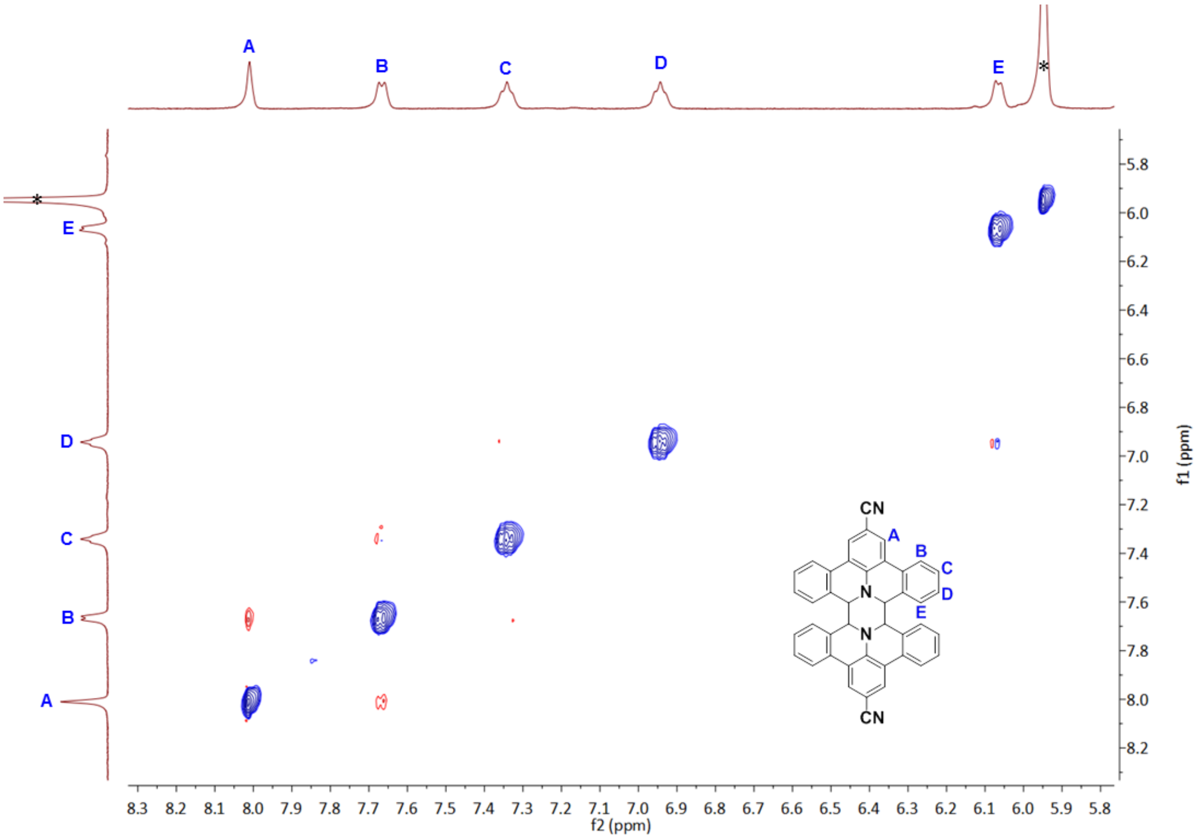

Supplementary Fig. 25 | ^{1}H-^{1}H NOESY spectrum of compound **8b** (500 MHz, $C_2D_2Cl_4$, 413 K). *Deuterated solvent C_2HDCl_4.

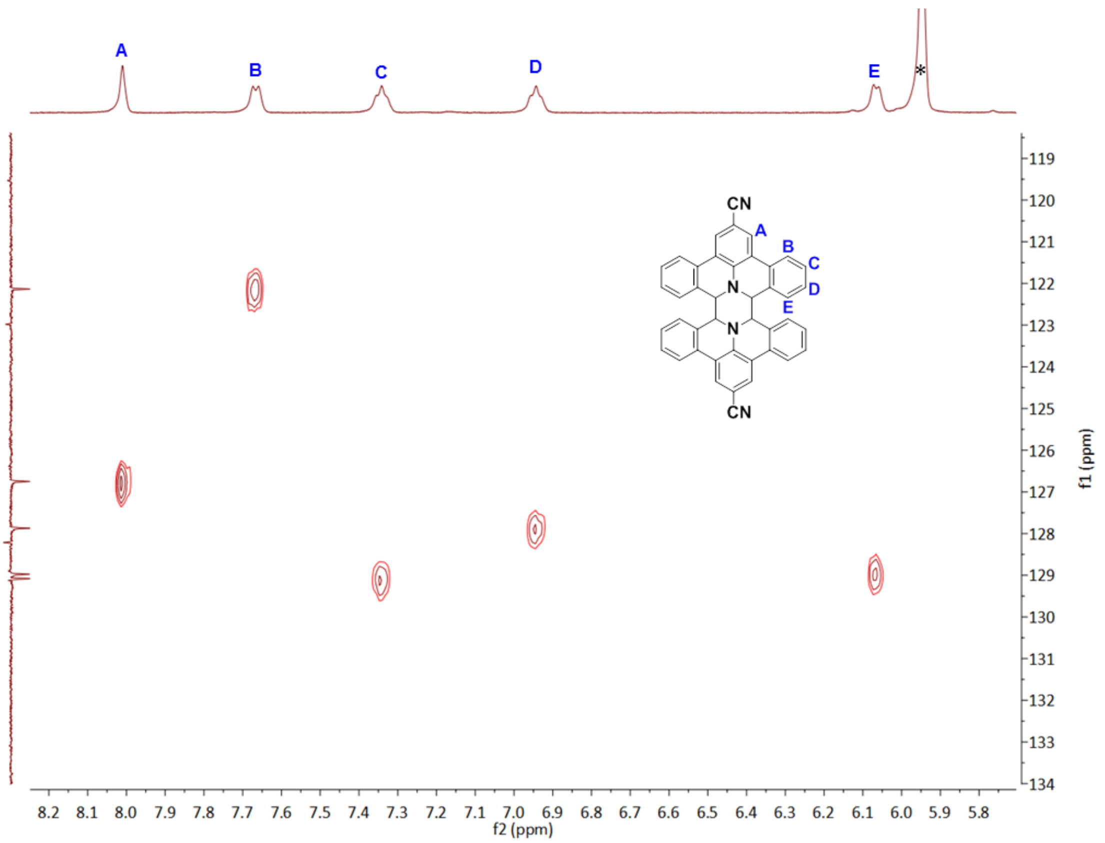

Supplementary Fig. 26 | ^{1}H-^{13}C HSQC spectrum of compound **8b** (500 MHz, $C_2D_2Cl_4$, 413 K). *Deuterated solvent C_2HDCl_4.

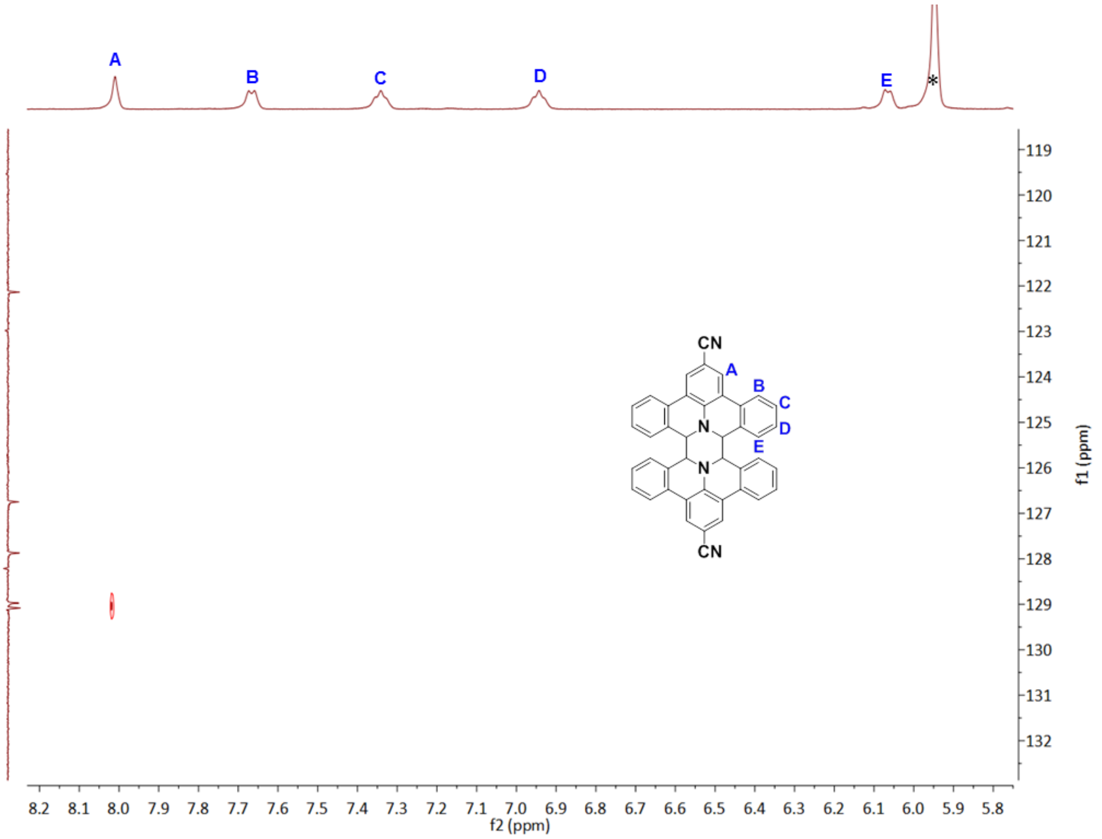

Supplementary Fig. 27 | ^{1}H-^{13}C HMBC spectrum of compound **8b** (500 MHz, $C_2D_2Cl_4$, 413 K). *Deuterated solvent C_2HDCl_4. Only a weak signal was recorded due to the low solubility of this molecule.

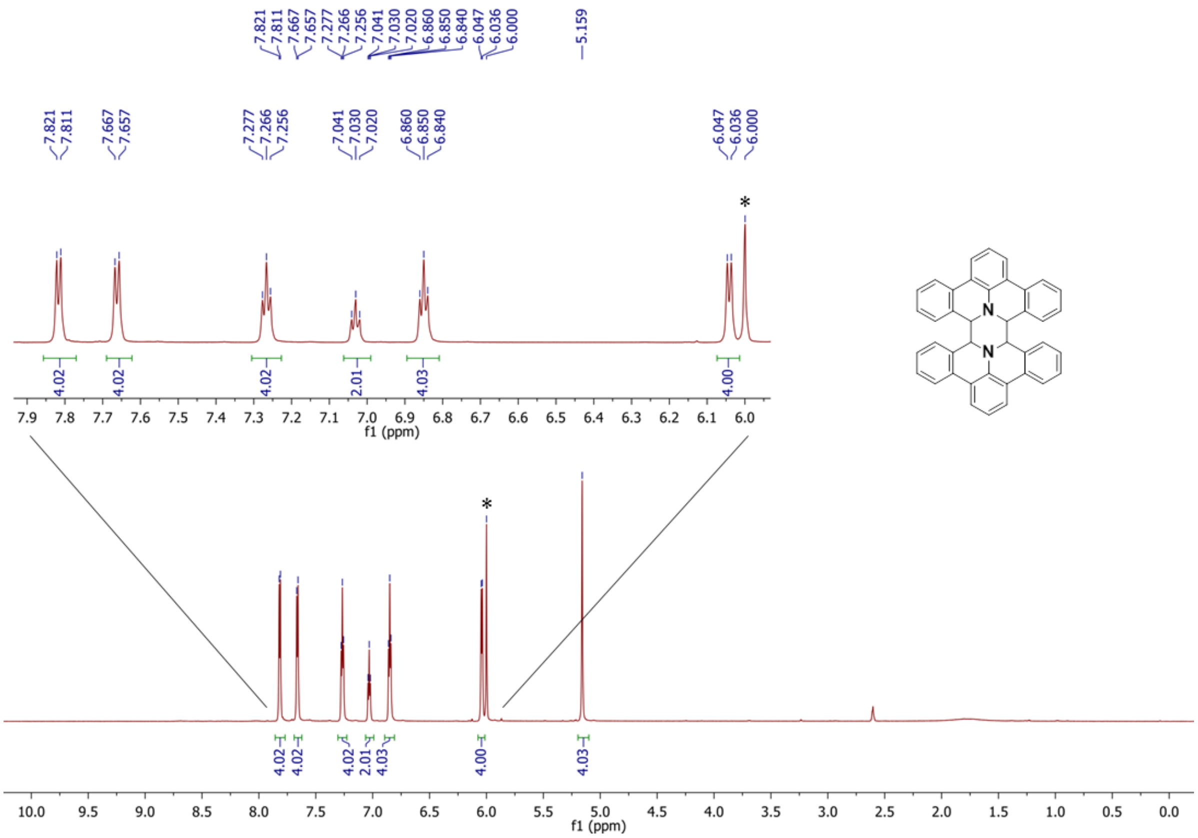

Supplementary Fig. 28 | ^{1}H NMR spectrum of compound **8c** (700 MHz, $C_2D_2Cl_4$, 323 K). *Deuterated solvent C_2HDCl_4.

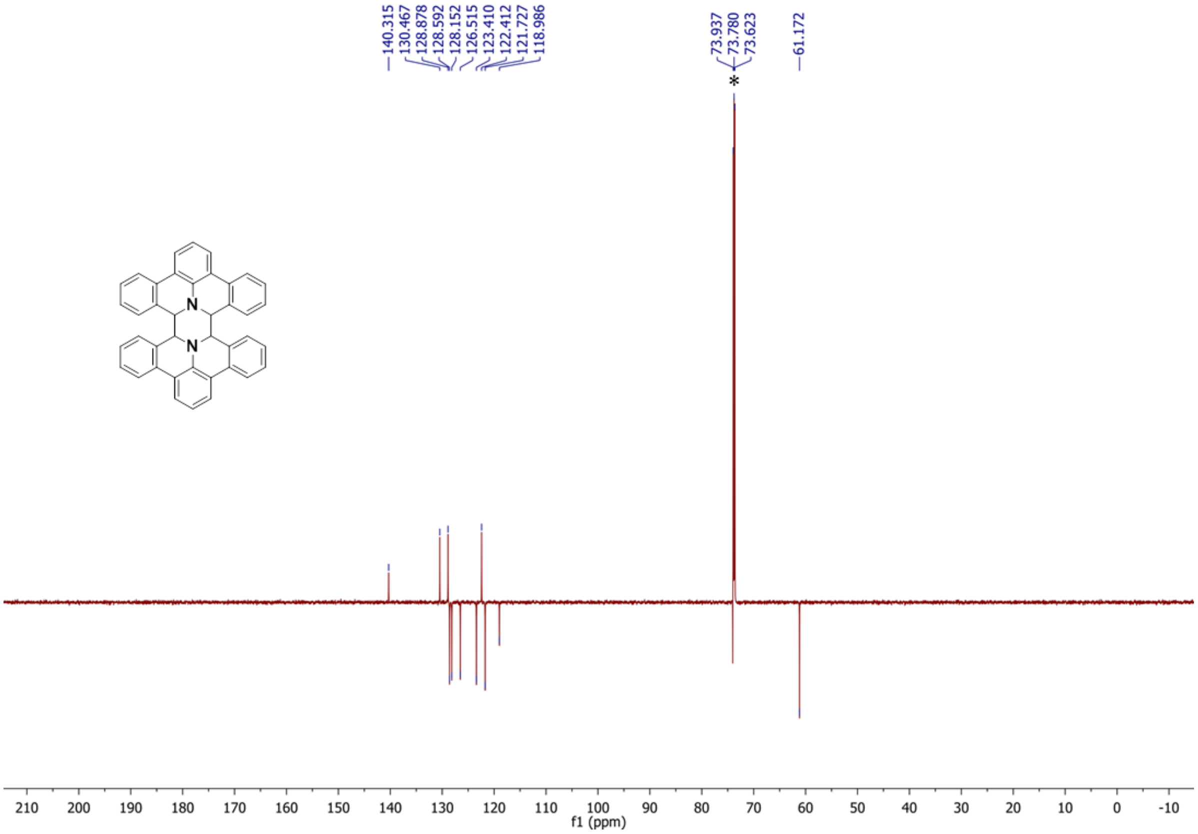

Supplementary Fig. 29 | ^{13}C NMR (APT) spectrum of compound **8c** (175 MHz, $C_2D_2Cl_4$, 323 K). *Deuterated solvent $C_2D_2Cl_4$.

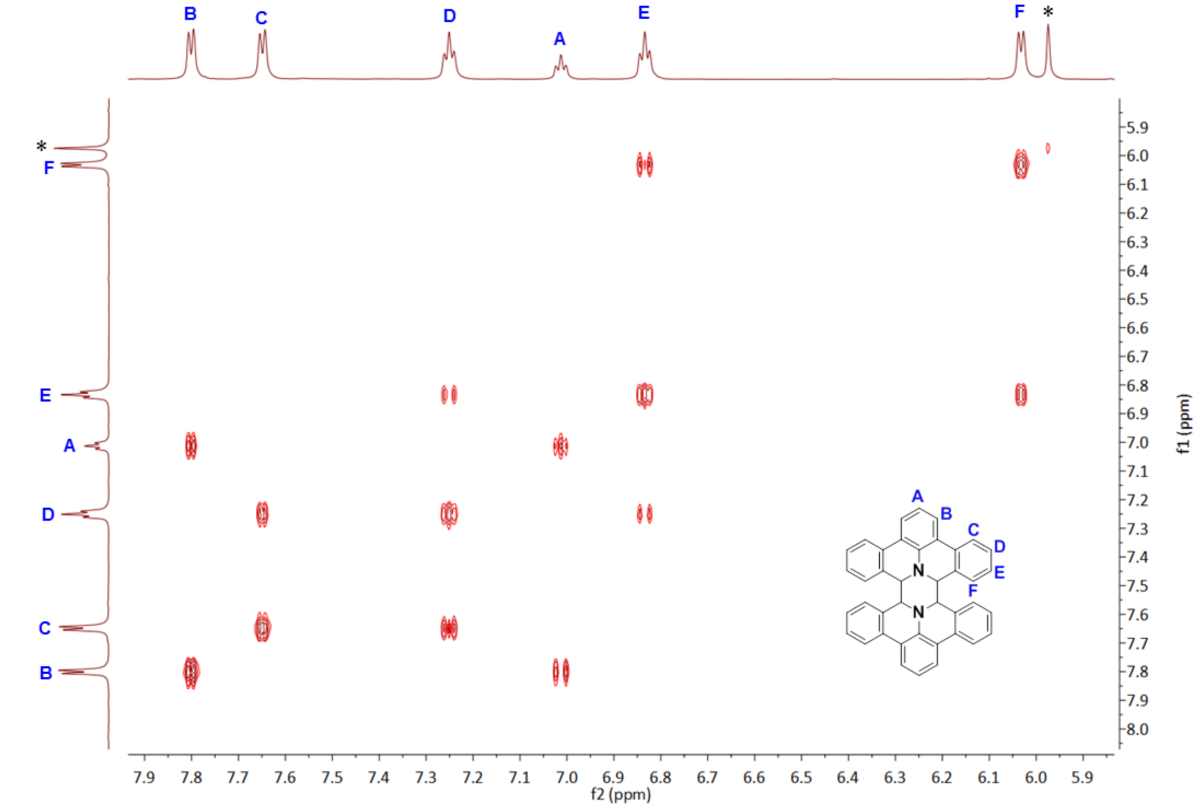

Supplementary Fig. 30 | ^1H-^1H COSY spectrum of compound **8c** (700 MHz, $C_2D_2Cl_4$, 323 K). *Deuterated solvent C_2HDCl_4.

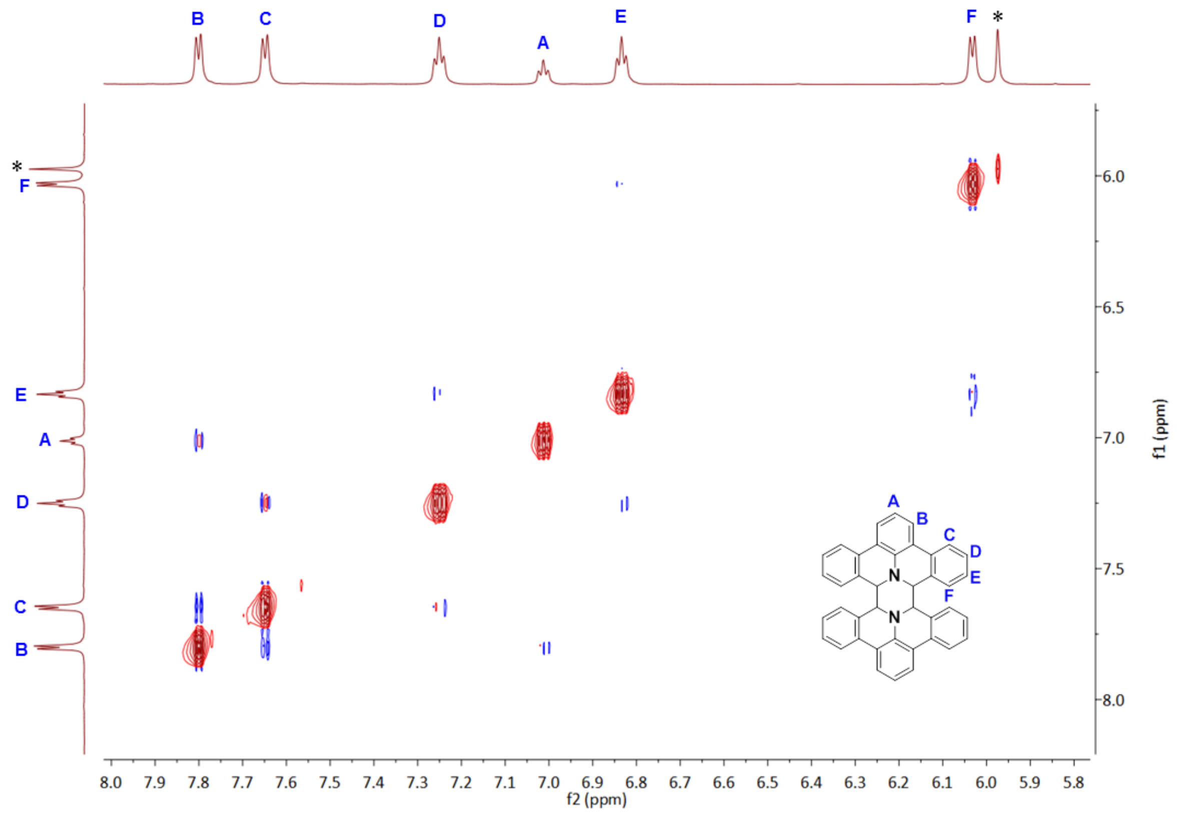

Supplementary Fig. 31 | ^{1}H-^{1}H NOESY spectrum of compound **8c** (700 MHz, $C_2D_2Cl_4$, 323 K). *Deuterated solvent C_2HDCl_4.

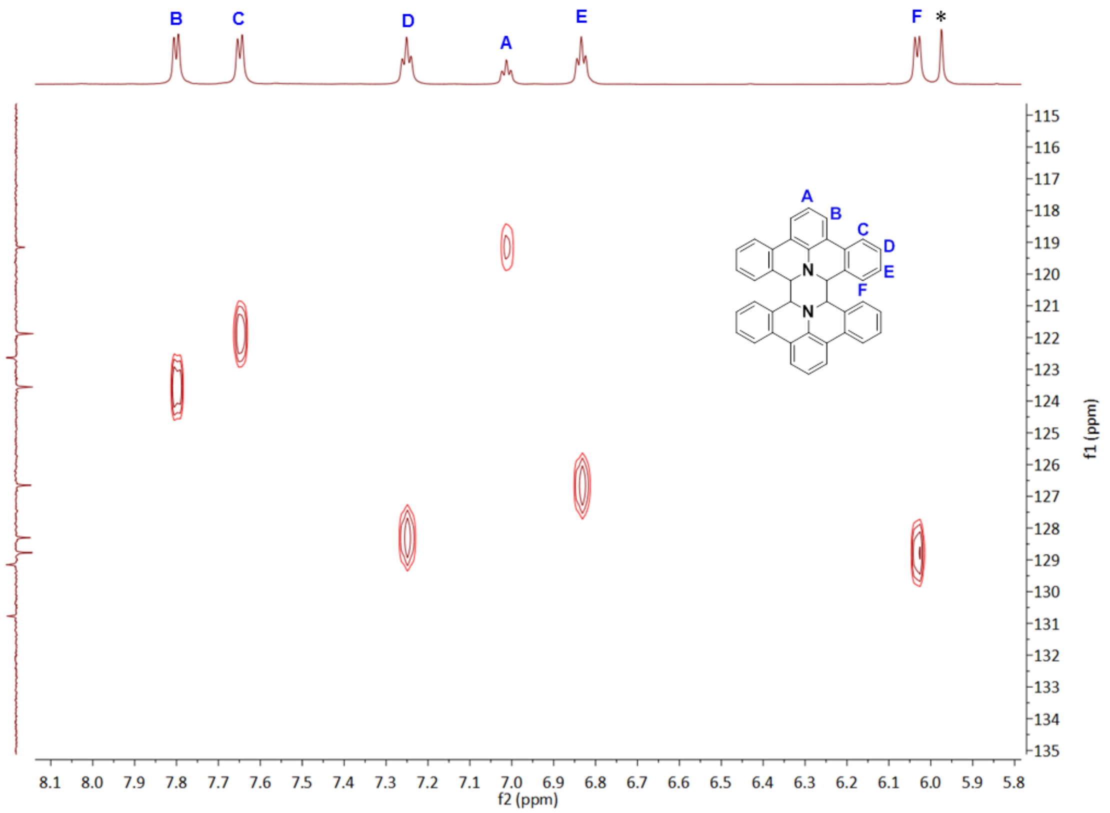

Supplementary Fig. 32 | ^{1}H-^{13}C HSQC spectrum of compound **8c** (700 MHz, $C_2D_2Cl_4$, 323 K). *Deuterated solvent C_2HDCl_4.

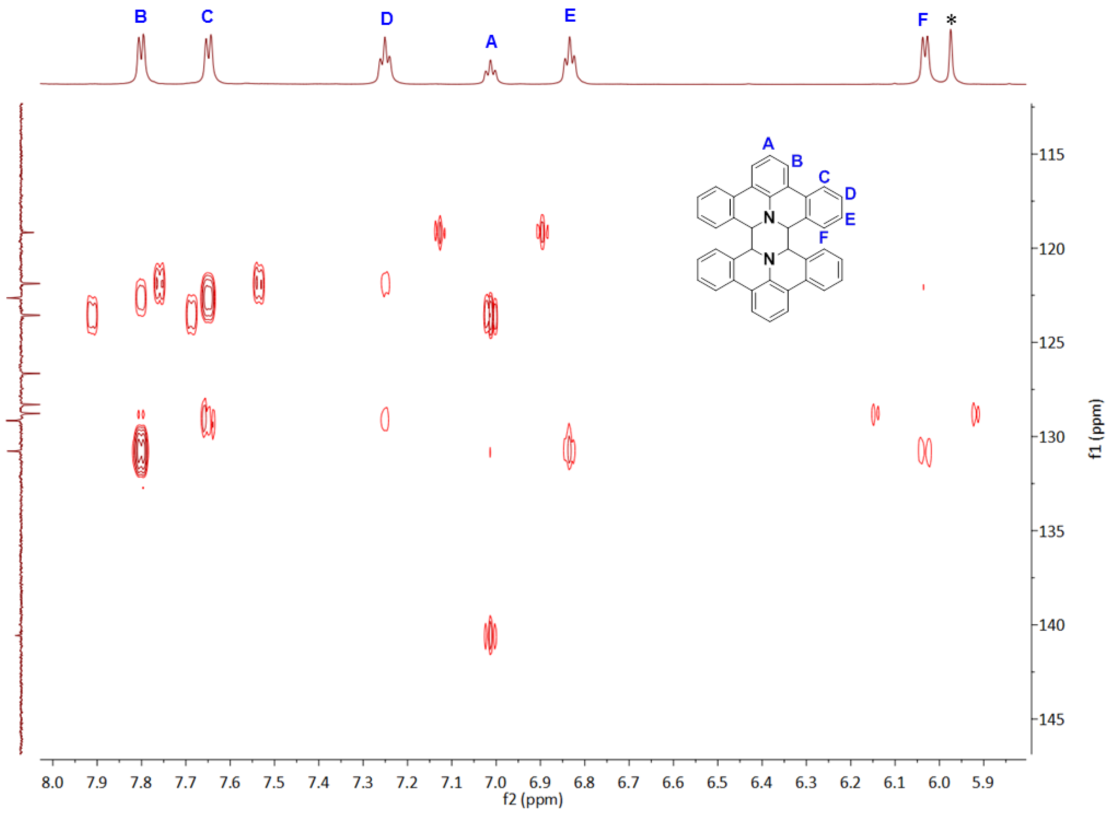

Supplementary Fig. 33 | ${}^{1}H$-${}^{13}C$ HMBC spectrum of compound **8c** (700 MHz, $C_2D_2Cl_4$, 323 K). *Deuterated solvent C_2HDCl_4.

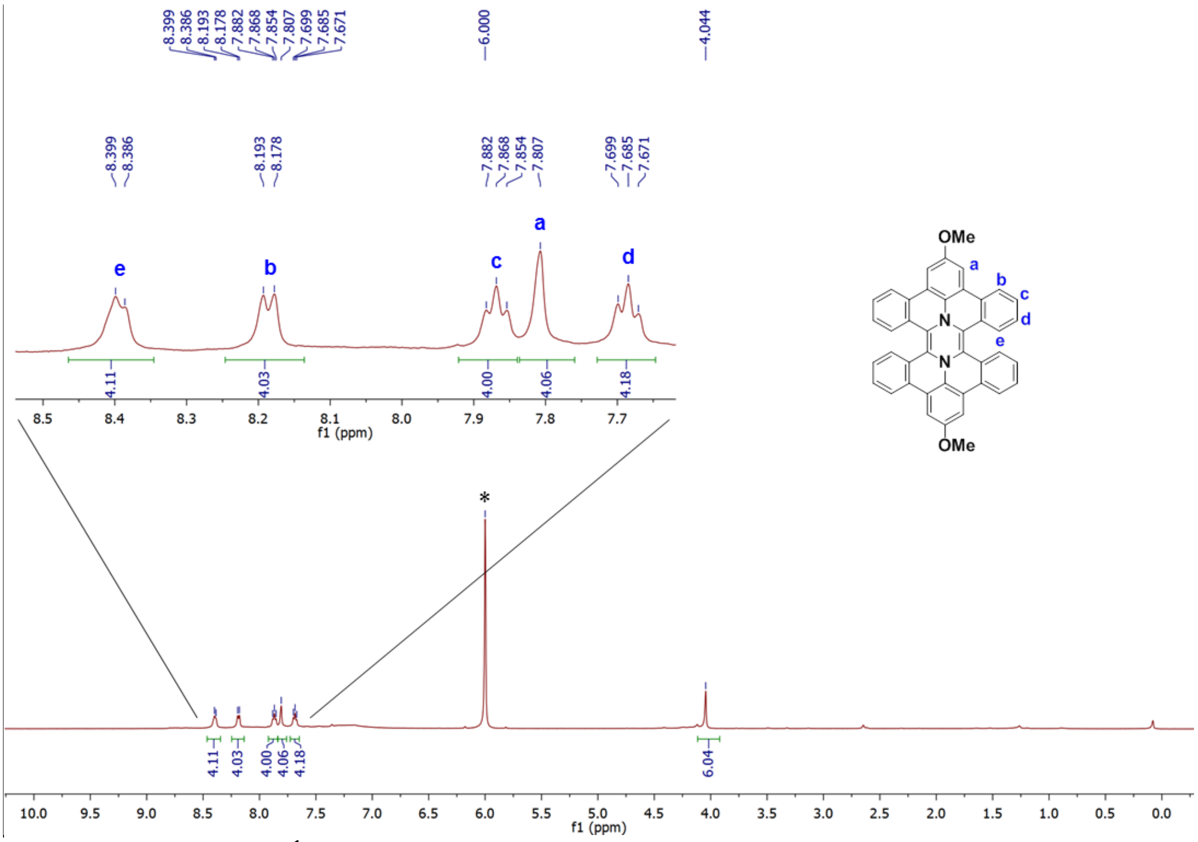

Supplementary Fig. 34 | ^{1}H NMR spectrum of the *in situ* generated **6a** (500 MHz, $C_2D_2Cl_4$, 298 K).

*Deuterated solvent C_2HDCl_4.

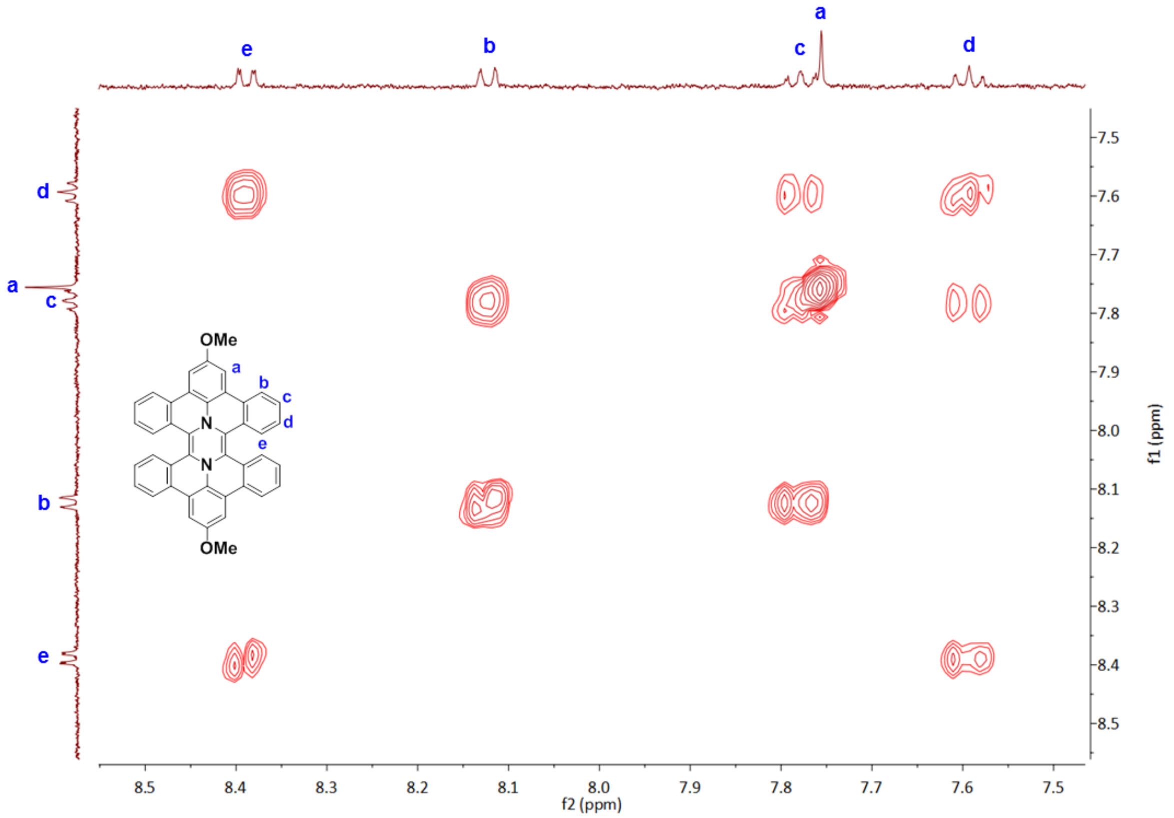

Supplementary Fig. 35 | ^{1}H-^{1}H COSY spectrum of the *in situ* generated **6a** (500 MHz, $C_2D_2Cl_4$, 298 K).

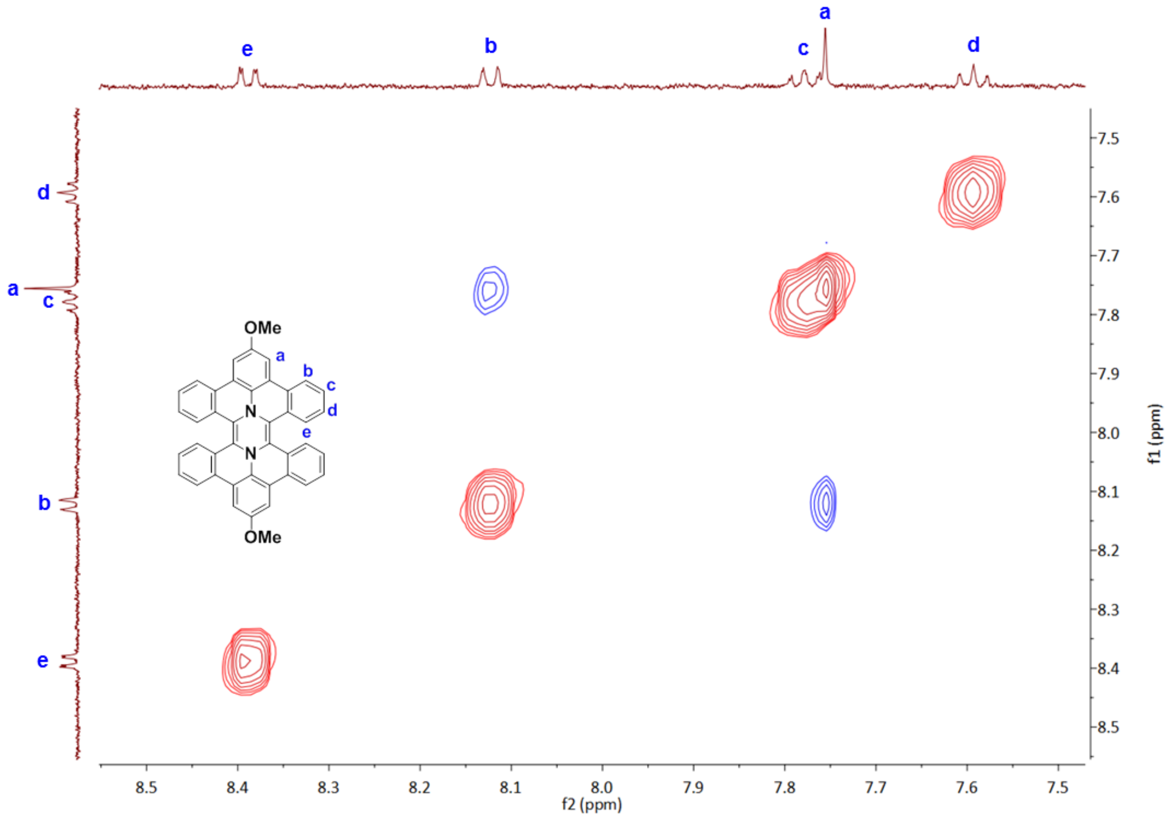

Supplementary Fig. 36 | ^{1}H-^{1}H NOESY spectrum of the *in situ* generated **6a** (500 MHz, $C_2D_2Cl_4$, 298 K).

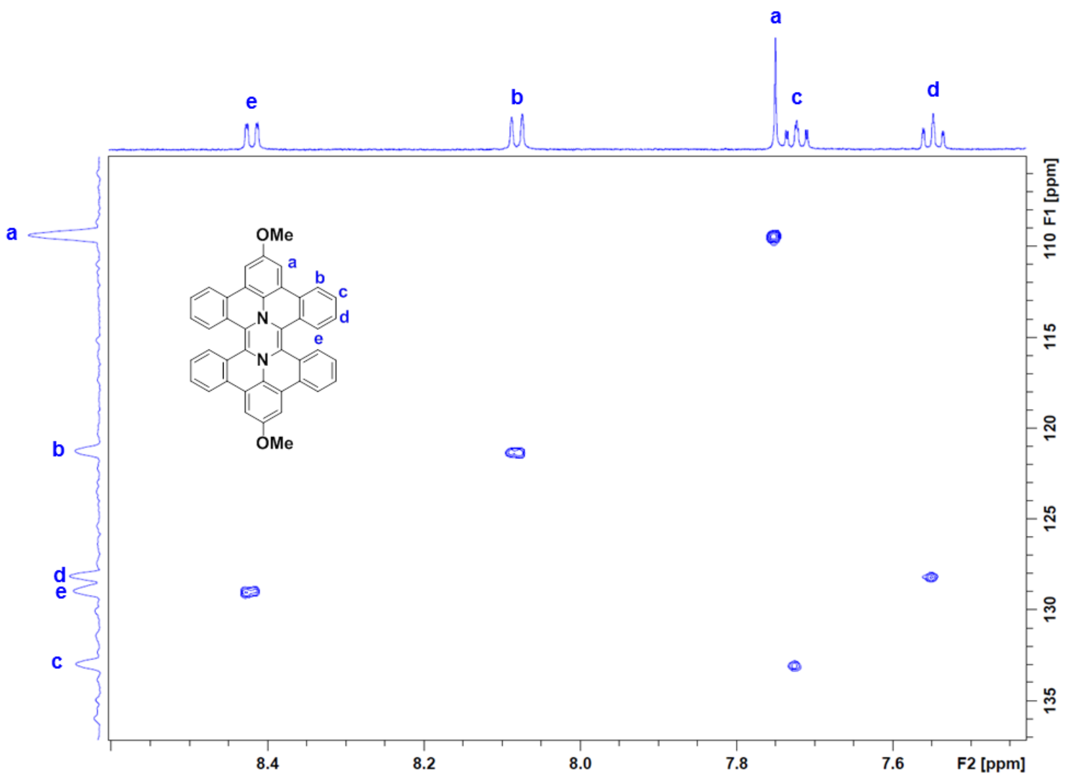

Supplementary Fig. 37 | ^{1}H-^{13}C HSQC spectrum of the *in situ* generated **6a** (600 MHz, $C_2D_2Cl_4$, 373 K).

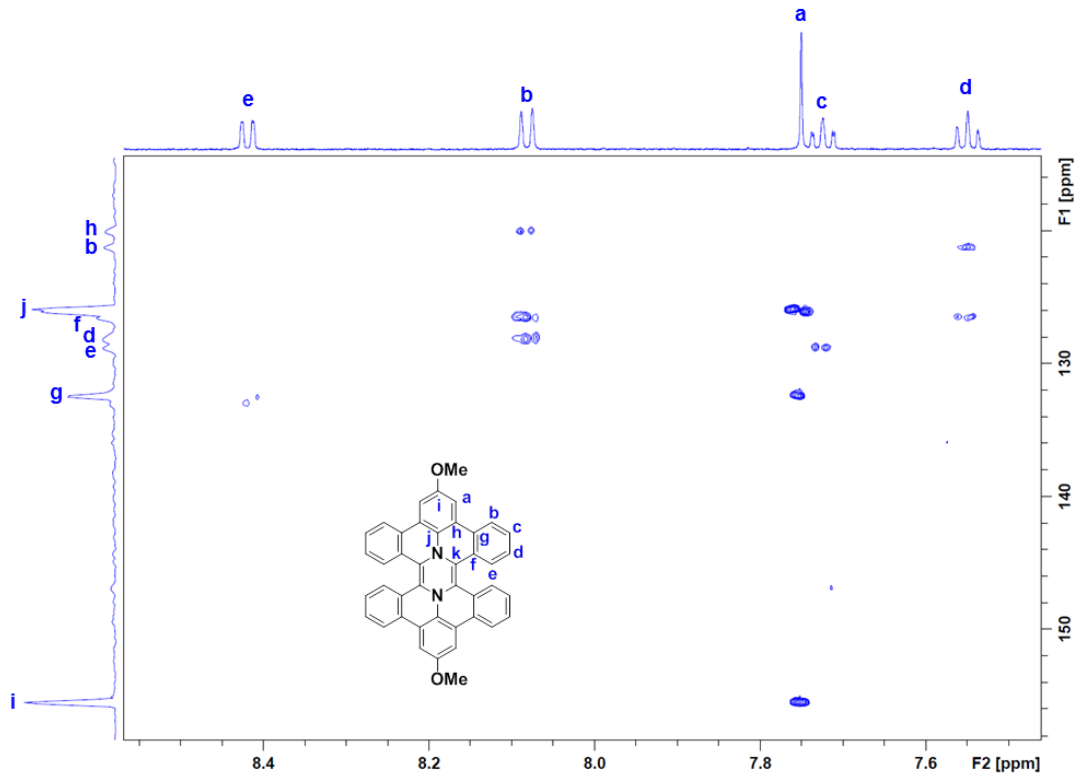

Supplementary Fig. 38 | ^{1}H-^{13}C HMBC spectrum of the *in situ* generated **6a** (600 MHz, $C_2D_2Cl_4$, 373 K).

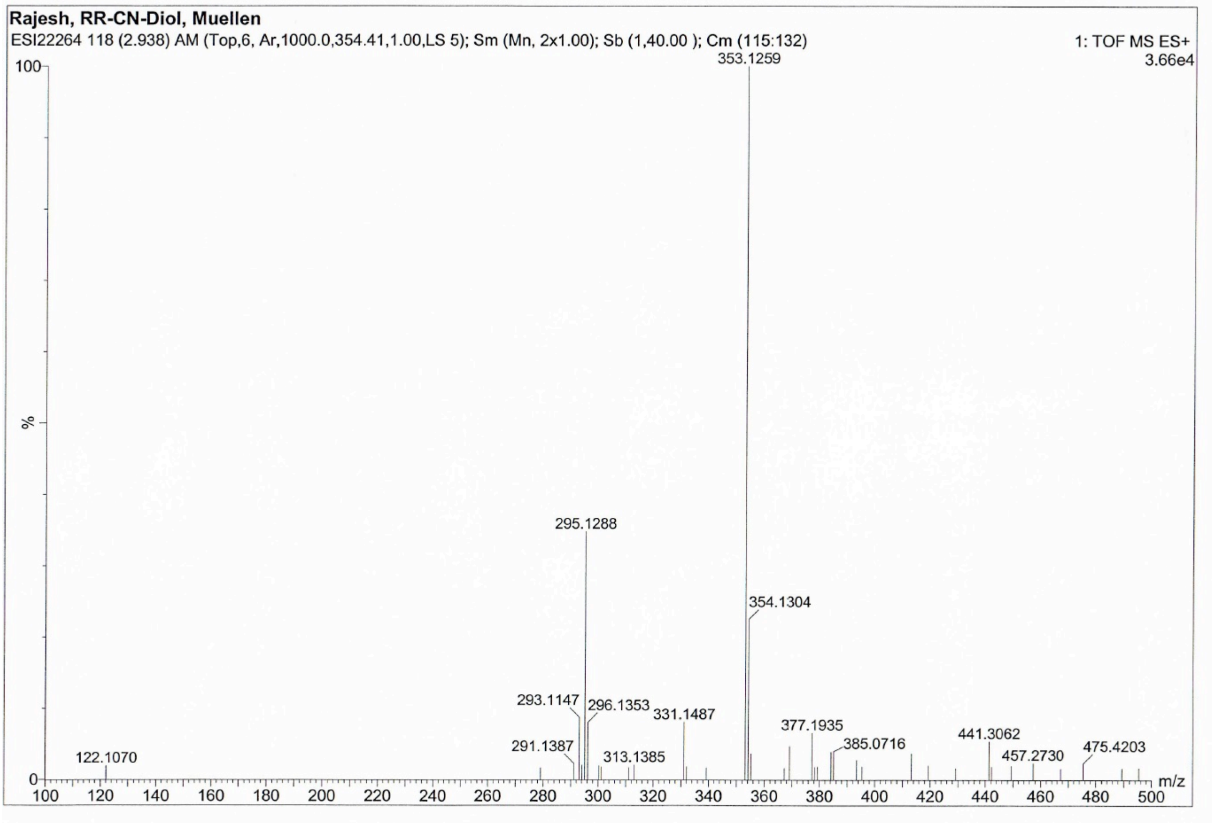

Single Mass Analysis

Tolerance = 20.0 PPM / DBE: min = -1.5, max = 200.0

Isotope cluster parameters: Separation = 1.0 Abundance = 1.0%

Monoisotopic Mass, Odd and Even Electron Ions

23 formula(e) evaluated with 1 results within limits (up to 50 closest results for each mass)

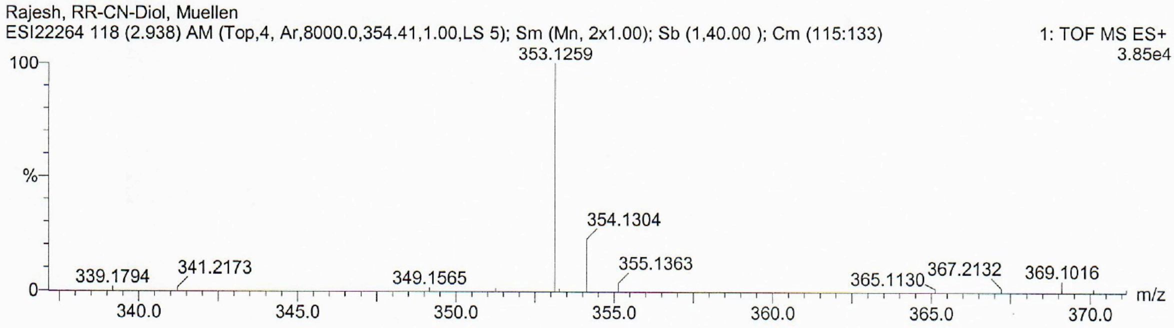

Minimum:				-1.5		
Maximum:		200.0	20.0	200.0		
Mass	Calc. Mass	mDa	PPM	DBE	Score	Formula
353.1259	353.1266	-0.7	-2.0	13.5	1	C21 H18 N2 O2 23Na

Supplementary Fig. 39 | HRMS (ESI) report of compound **11b**.

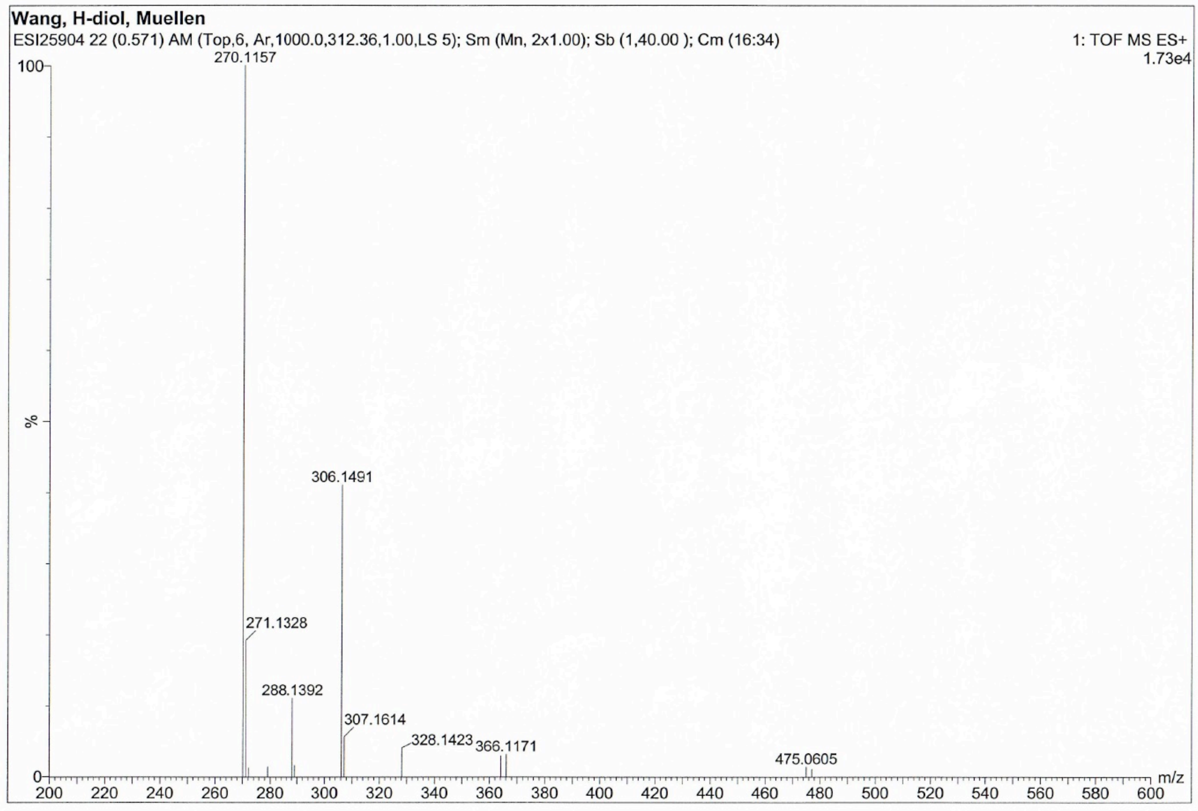

Single Mass Analysis

Tolerance = 20.0 PPM / DBE: min = -1.5, max = 200.0

Isotope cluster parameters: Separation = 1.0 Abundance = 1.0%

Monoisotopic Mass, Odd and Even Electron Ions

200 formula(e) evaluated with 1 results within limits (up to 50 closest results for each mass)

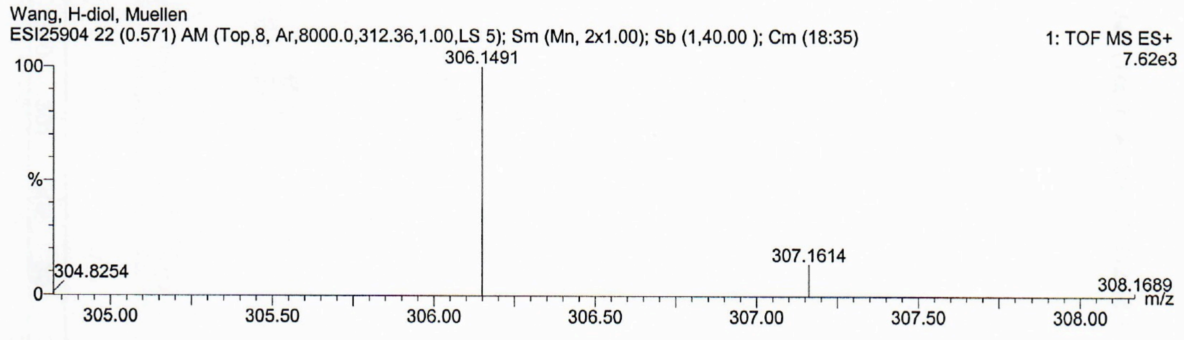

Mass	Calc. Mass	mDa	PPM	DBE	Score	Formula
Minimum:				-1.5		
Maximum:		200.0	20.0	200.0		
306.1491	306.1494	-0.3	-1.0	11.5	1	C20 H20 N O2

Supplementary Fig. 40 | HRMS (ESI) report of compound **11c**.

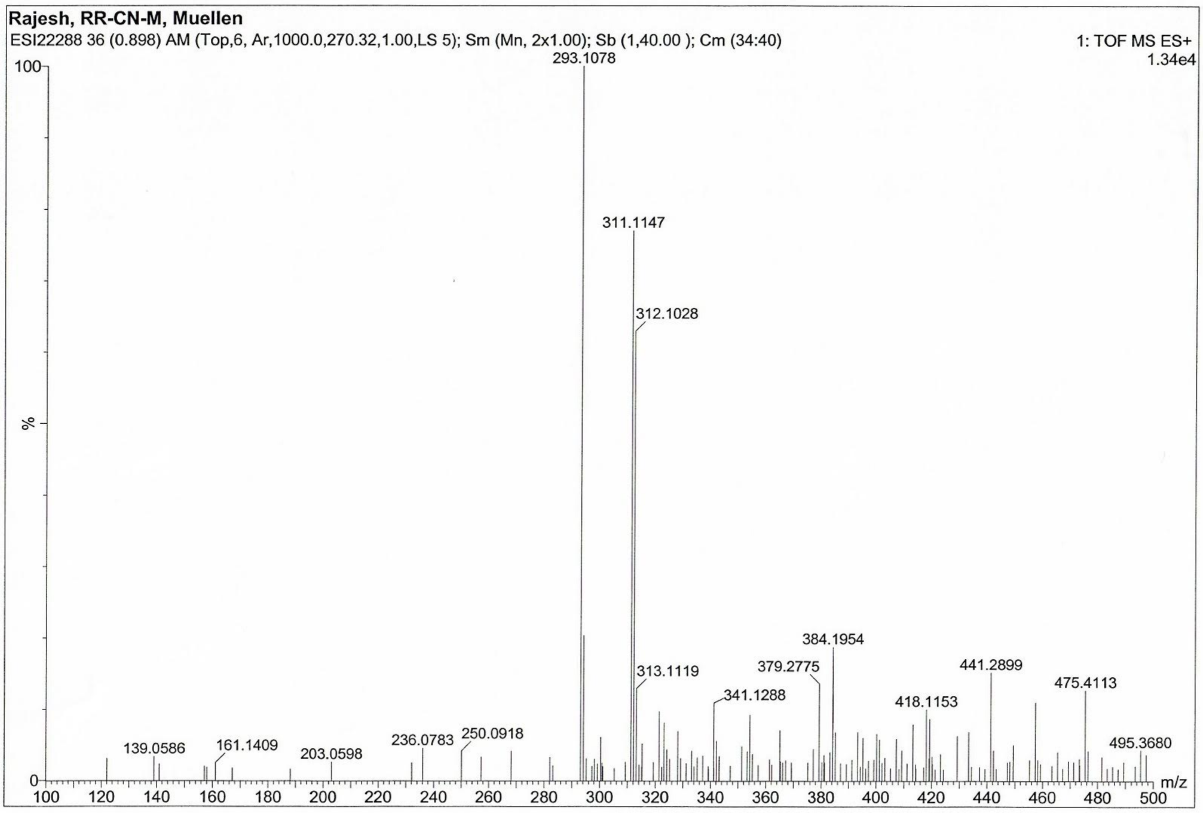

Single Mass Analysis (displaying only valid results)

Tolerance = 40.0 PPM / DBE: min = 1.0, max = 50.0

Isotope cluster parameters: Separation = 1.0 Abundance = 1.0%

Monoisotopic Mass, Odd and Even Electron Ions

2 formula(e) evaluated with 1 results within limits (up to 50 closest results for each mass)

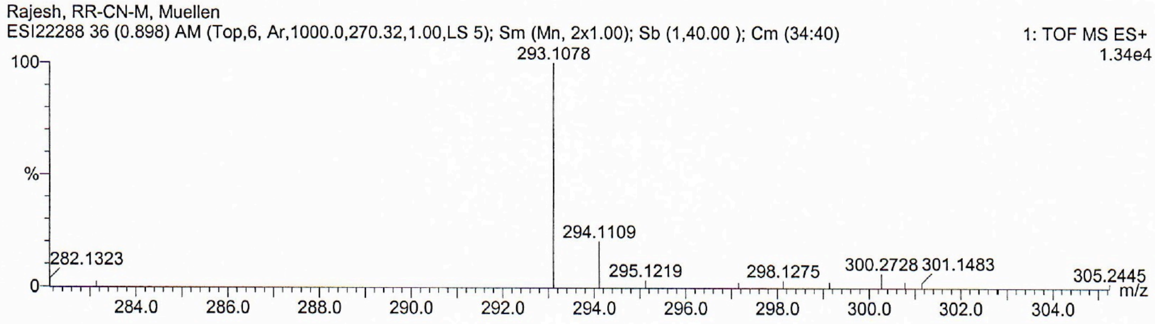

Minimum:				1.0		
Maximum:		200.0	40.0	50.0		
Mass	Calc. Mass	mDa	PPM	DBE	Score	Formula
293.1078	293.1079	-0.1	-0.3	16.5	1	C21 H13 N2

Supplementary Fig. 41 | HRMS (ESI) report of compound **5b**.

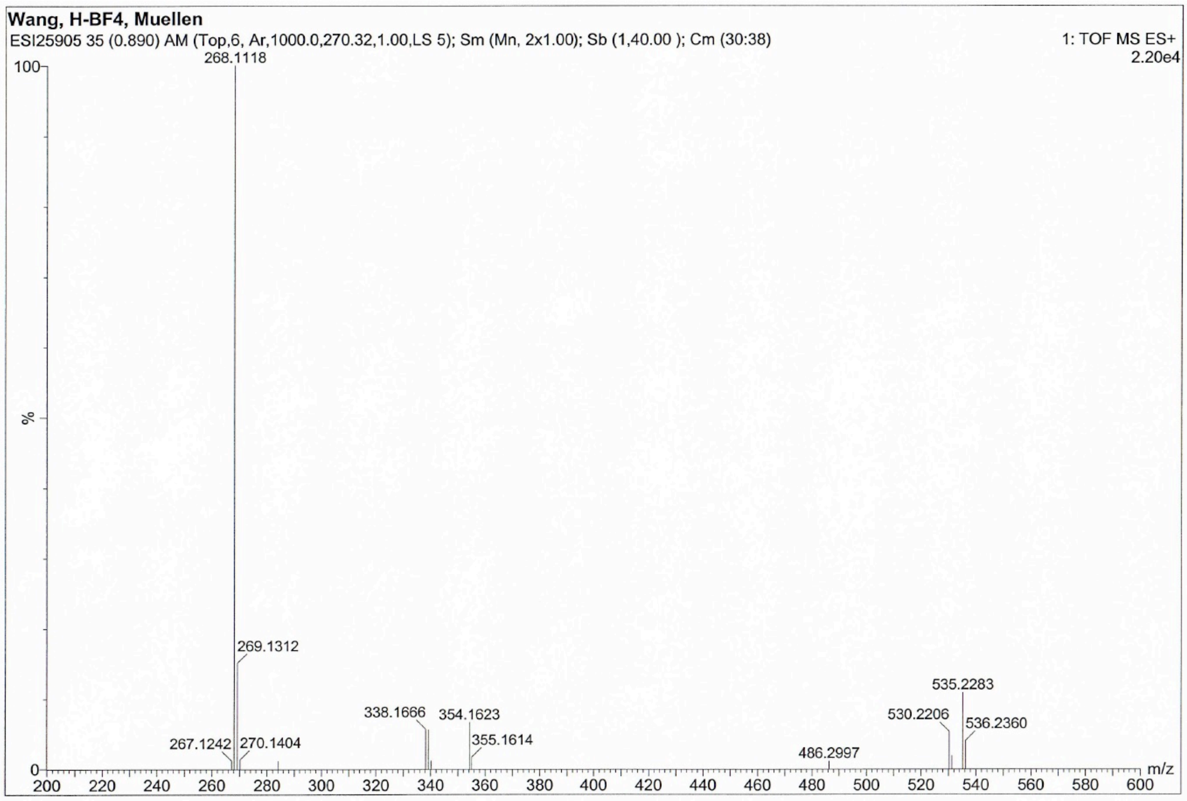

Single Mass Analysis

Tolerance = 20.0 PPM / DBE: min = -1.5, max = 200.0

Isotope cluster parameters: Separation = 1.0 Abundance = 1.0%

Monoisotopic Mass, Odd and Even Electron Ions

17 formula(e) evaluated with 1 results within limits (up to 50 closest results for each mass)

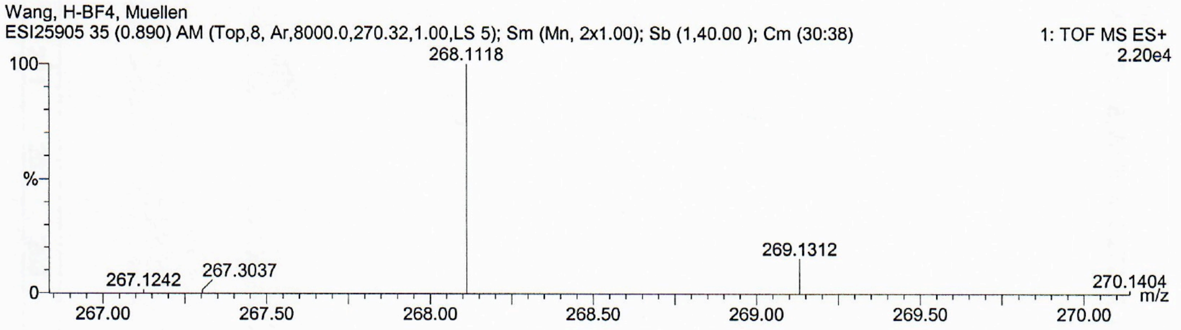

Minimum:				-1.5		
Maximum:		200.0	20.0	200.0		
Mass	Calc. Mass	mDa	PPM	DBE	Score	Formula
268.1118	268.1126	-0.8	-3.1	14.5	1	C20 H14 N

Supplementary Fig. 42 | HRMS (ESI) report of compound **5c**.

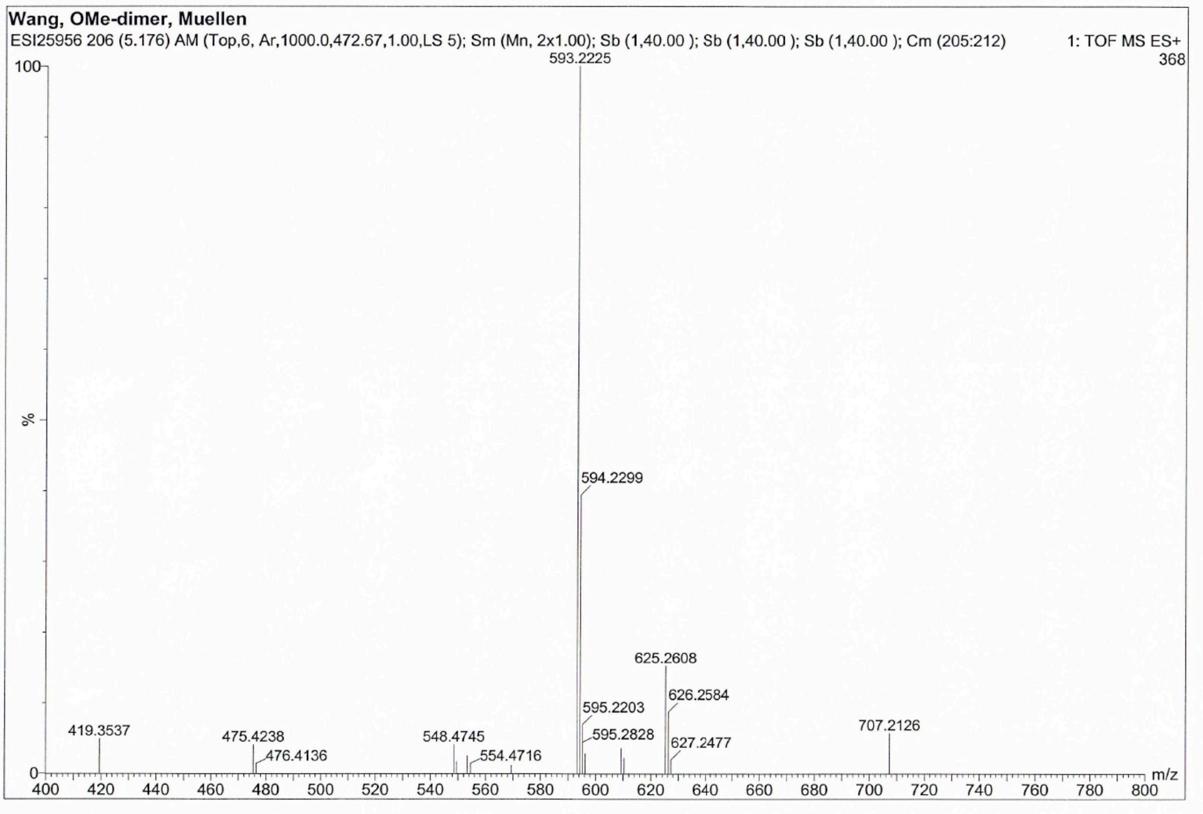

Single Mass Analysis (displaying only valid results)
Tolerance = 40.0 PPM / DBE: min = 1.0, max = 50.0
Isotope cluster parameters: Separation = 1.0 Abundance = 1.0%

Monoisotopic Mass, Odd and Even Electron Ions
3 formula(e) evaluated with 1 results within limits (up to 50 closest results for each mass)

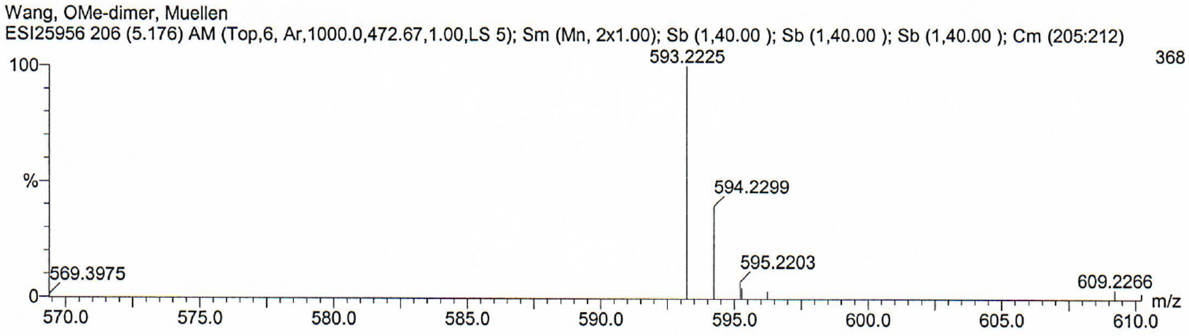

Minimum:				1.0		
Maximum:		200.0	40.0	50.0		
Mass	Calc. Mass	mDa	PPM	DBE	Score	Formula
593.2225	593.2229	-0.4	-0.7	29.5	1	C42 H29 N2 O2

Supplementary Fig. 43 | HRMS (ESI) report of compound **8a**.

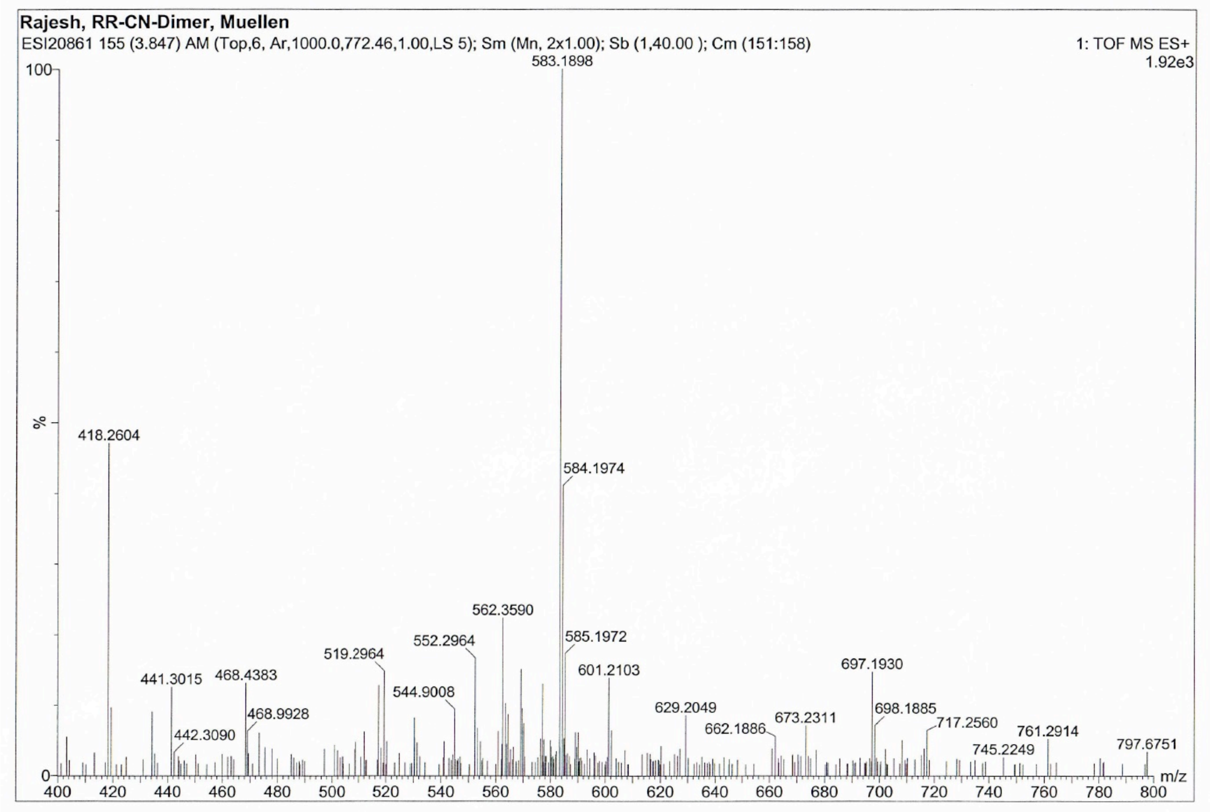

Single Mass Analysis

Tolerance = 10.0 PPM / DBE: min = -1.5, max = 200.0

Isotope cluster parameters: Separation = 1.0 Abundance = 1.0%

Monoisotopic Mass, Odd and Even Electron Ions

4 formula(e) evaluated with 1 results within limits (up to 50 closest results for each mass)

Rajesh, RR-CN-Dimer, Muellen

ESI20861 155 (3.847) AM (Top,4, Ar,8000.0,772.46,1.00,LS 5); Sm (Mn, 2x1.00); Sb (1,40.00); Sb (1,40.00); Sb (1,40.00); Cm (149:157)

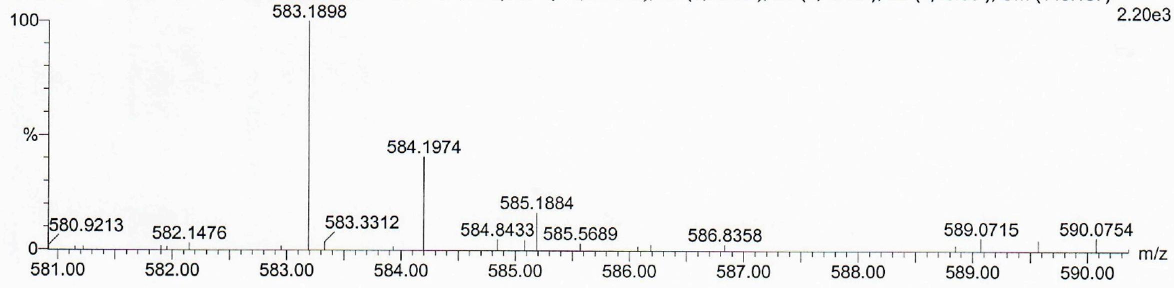

Minimum:				-1.5		
Maximum:		200.0	10.0	200.0		
Mass	Calc. Mass	mDa	PPM	DBE	Score	Formula
583.1898	583.1923	-2.5	-4.2	33.5	1	C42 H23 N4

Supplementary Fig. 44 | HRMS (ESI) report of compound **8b**.

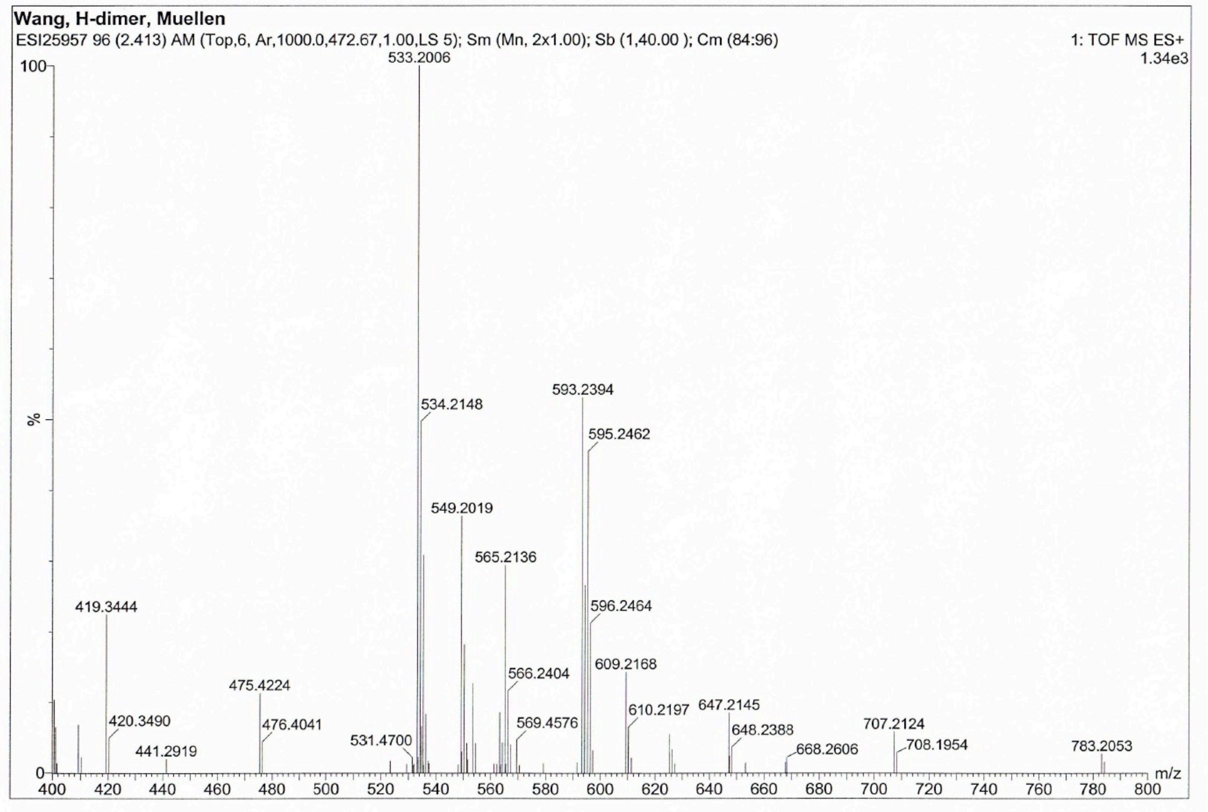

Single Mass Analysis
Tolerance = 20.0 PPM / DBE: min = -1.5, max = 200.0
Isotope cluster parameters: Separation = 1.0 Abundance = 1.0%

Monoisotopic Mass, Odd and Even Electron Ions
1 formula(e) evaluated with 1 results within limits (up to 50 closest results for each mass)

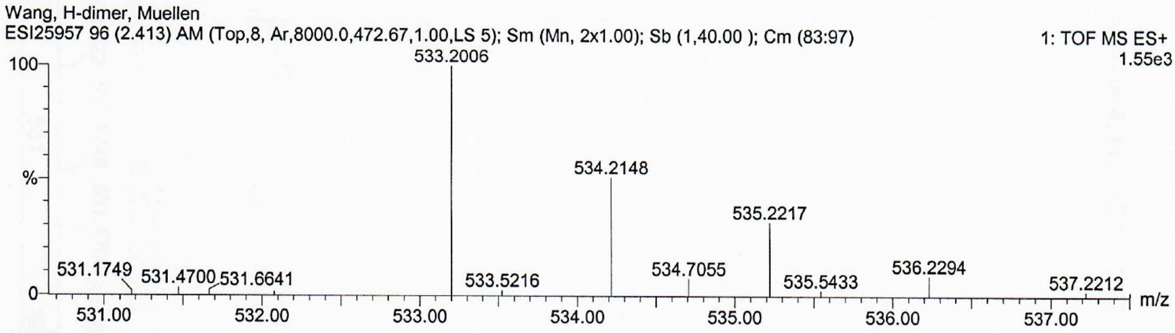

Minimum:				-1.5		
Maximum:		200.0	20.0	200.0		
Mass	Calc. Mass	mDa	PPM	DBE	Score	Formula
533.2006	533.2018	-1.2	-2.2	29.5	1	C40 H25 N2

Supplementary Fig. 45 | HRMS (ESI) report of compound **8c**.

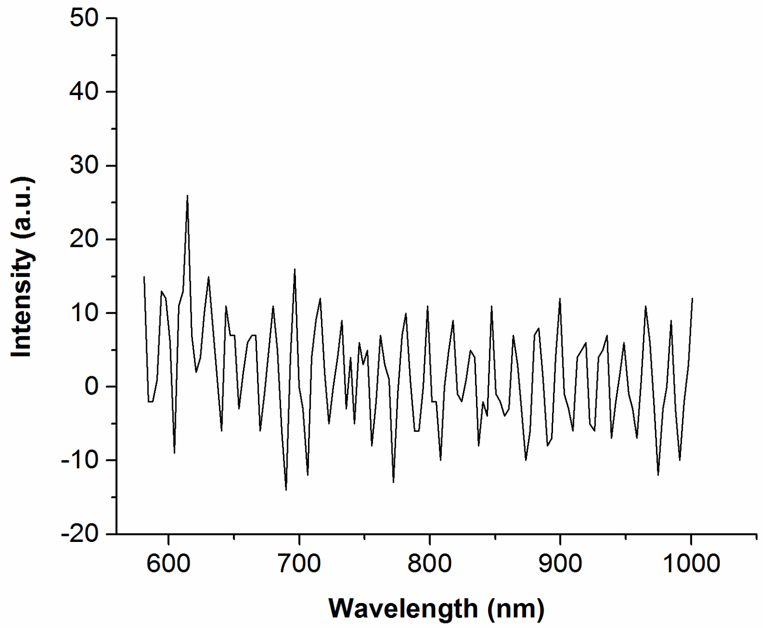

Supplementary Fig. 46 | Photoluminescence of the *in situ* generated **6a** in $C_2H_2Cl_4$ excited at 550 nm, exhibiting no detectable fluorescence.

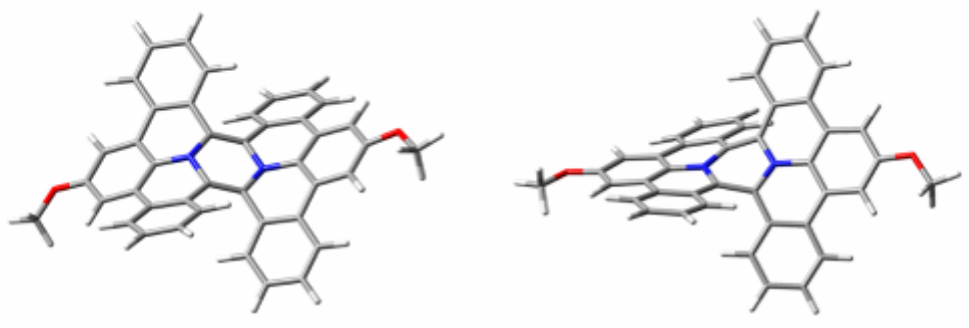

anti-folded **6a**
0.00 kcal/mol

twisted **6a**
4.81 kcal/mol

Supplementary Fig. 47 | Two conformations of compound **6a** and their relative energies calculated at the B3LYP/6-311G(d,p) level, showing that the *anti*-folded conformer is favored in energy.

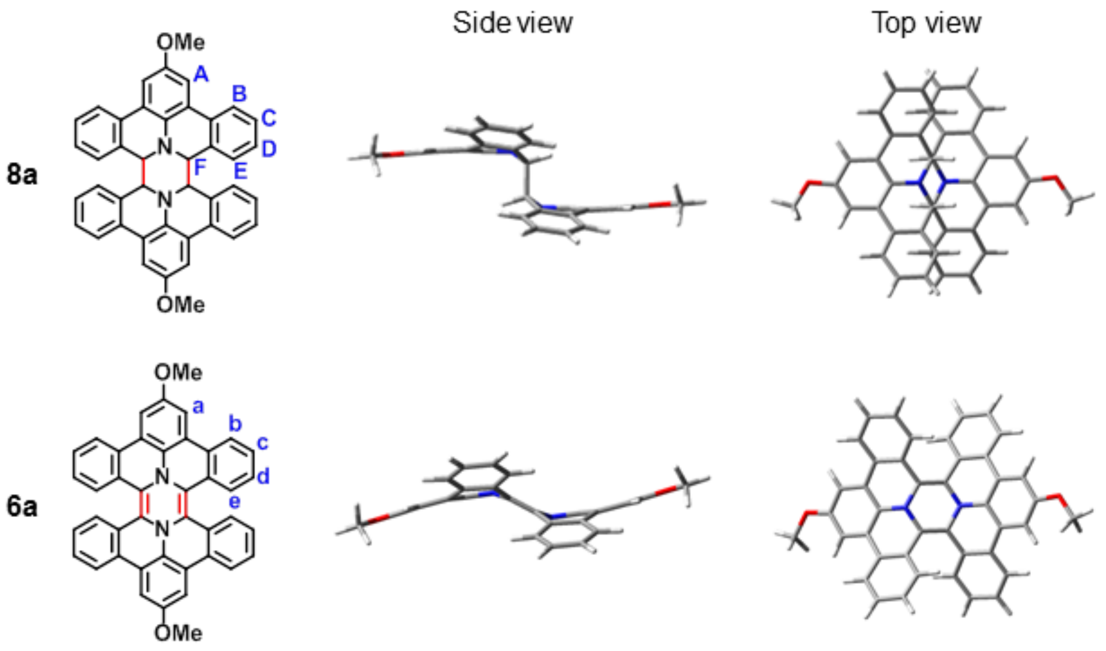

Supplementary Fig. 48 | DFT-optimized structures of **8a** and **6a** calculated at the B3LYP/6-311G(d,p) level. In particular, H_E of **8a** is above the neighboring benzene ring, and thus in its shielding region. In contrast, after dehydrogenation, H_e of **6a** is away from the neighboring benzene ring and moves into its deshielding region.

Supplementary Table 1. Major electronic transitions of **6a** calculated by TDDFT method.

excited state	energy (eV)	wavelength (nm)	oscillator strength	configuration
1	1.49	830	0.0000	HOMO→LUMO (0.70341)
2	2.04	607	0.0005	HOMO→LUMO+1 (0.70273)
3	2.15	577	0.0582	HOMO→LUMO+2 (0.70459)
4	2.42	512	0.0159	HOMO→LUMO+3 (0.68215)<br>HOMO→LUMO+4 (0.17472)
5	2.66	466	0.0770	HOMO→LUMO+3 (−0.17247)<br>HOMO→LUMO+4 (0.67769)
6	2.68	462	0.0001	HOMO→LUMO+5 (0.70392)
7	3.24	383	0.0010	HOMO→LUMO+6 (0.69105)<br>HOMO→LUMO+7 (0.11069)
8	3.28	377	0.0086	HOMO→LUMO+6 (−0.11273)<br>HOMO→LUMO+7 (0.69108)
9	3.36	369	0.1171	HOMO−1→LUMO (0.68905)<br>HOMO−1→LUMO+1 (0.10886)
10	3.46	358	0.1610	HOMO−2→LUMO (0.63874)<br>HOMO→LUMO+8 (0.27365)

Supplementary Table 2. Crystal data and structure refinement for compound **8c**.

Empirical formula	$C_{42}H_{28}Cl_4N_2$
Formula weight	702.46
Temperature/K	100.0(2)
Crystal system	monoclinic
Space group	$P2_1/n$
a/Å	15.3518(2)
b/Å	6.81740(10)
c/Å	30.6440(3)
α/°	90
β/°	90.9739(11)
γ/°	90
Volume/Å^3	3206.72(7)
Z	4
ρ_{calc}g/cm^3	1.455
μ/mm^{-1}	3.630
F(000)	1448.0
Crystal size/mm^3	0.276 × 0.054 × 0.013
Radiation	CuKα (λ = 1.54184)
2Θ range for data collection/°	5.768 to 153.242
Index ranges	$-19 \le h \le 18$, $-8 \le k \le 8$, $-38 \le l \le 33$
Reflections collected	18624
Independent reflections	6678 [R_{int} = 0.0330, R_{sigma} = 0.0336]
Data/restraints/parameters	6678/16/562
Goodness-of-fit on F^2	1.041
Final R indexes [I>=2σ (I)]	R_1 = 0.0381, wR_2 = 0.1013
Final R indexes [all data]	R_1 = 0.0431, wR_2 = 0.1063
Largest diff. peak/hole / e Å^{-3}	0.33/-0.51

Supplementary Table 3. Major electronic transitions of **8a** calculated by TDDFT method.

excited state	energy (eV)	wavelength (nm)	oscillator strength	configuration
1	2.59	479	0.1168	HOMO→LUMO (0.70388)
2	2.65	468	0.0002	HOMO→LUMO+1 (0.70161)
3	2.98	415	0.0001	HOMO→LUMO+2 (0.70300)
4	3.46	358	0.0052	HOMO−1→LUMO (0.50325)<br>HOMO→LUMO+3 (−0.23023)<br>HOMO→LUMO+4 (0.42734)
5	3.50	354	0.0275	HOMO−1→LUMO (0.13883)<br>HOMO→LUMO+3 (0.66215)<br>HOMO→LUMO+4 (0.18903)
6	3.51	353	0.0005	HOMO−1→LUMO (−0.46456)<br>HOMO→LUMO+4 (0.52200)
7	3.60	344	0.0220	HOMO→LUMO+5 (0.69661)
8	3.61	344	0.1336	HOMO−1→LUMO+1 (0.67871)<br>HOMO→LUMO+7 (−0.13214)
9	3.67	337	0.0092	HOMO−1→LUMO+1 (−0.10318)<br>HOMO→LUMO+6 (0.68670)
10	3.74	332	0.0302	HOMO−1→LUMO+1 (0.11822)<br>HOMO→LUMO+7 (0.68303)

Supplementary Table 4. Cartesian Coordinates for NICS Calculations

Compound **6a**

Tag	Symbol	X	Y	Z
1	C	0.620093	1.282393	-0.274633
2	C	-0.620098	1.282392	0.274704
3	N	-1.339500	0.062195	0.465722
4	C	-0.620078	-1.157671	0.274489
5	C	0.620078	-1.157667	-0.274414
6	N	1.339492	0.062197	-0.465652
7	C	2.712285	0.060363	-0.186055
8	C	1.357994	-2.350919	-0.727916
9	C	-1.357997	-2.350918	0.727996
10	C	-2.712285	0.060364	0.186095
11	C	-1.356635	2.476678	0.727735
12	C	1.356617	2.476684	-0.727668
13	C	-2.756673	-2.373362	0.557074
14	C	-3.472170	-3.508664	0.968896
15	C	-2.824000	-4.588863	1.550803
16	C	-1.446530	-4.538454	1.771461
17	C	-0.725393	-3.421979	1.373120
18	C	0.725378	-3.421984	-1.373021
19	C	1.446505	-4.538470	-1.771350
20	C	2.823976	-4.588883	-1.550701
21	C	3.472159	-3.508676	-0.968820
22	C	2.756672	-2.373364	-0.557013
23	C	3.416018	-1.154808	-0.068649
24	C	4.747259	-1.140017	0.376005
25	C	5.395028	0.062233	0.638414
26	C	4.738155	1.263656	0.369984
27	C	3.417599	1.283137	-0.071272
28	C	2.754782	2.500871	-0.559615
29	C	3.470786	3.635439	-0.971117
30	C	2.821614	4.715179	-1.552054
31	C	1.443589	4.663792	-1.771245
32	C	0.722480	3.548182	-1.371700
33	C	-0.722518	3.548171	1.371790
34	C	-1.443641	4.663778	1.771323
35	C	-2.821660	4.715162	1.552090
36	C	-3.470813	3.635425	0.971125
37	C	-2.754796	2.500861	0.559636
38	C	-3.417593	1.283126	0.071277
39	C	-4.738149	1.263642	-0.370011
40	C	-5.395027	0.062223	-0.638420
41	C	-4.747235	-1.140031	-0.376015
42	C	-3.416020	-1.154816	0.068680
43	H	-4.550243	-3.530149	0.868203
44	H	-3.395315	-5.455223	1.864778
45	H	-0.940825	-5.360186	2.265168

46	H	0.339740	-3.374836	1.557062
47	H	-0.339755	-3.374835	-1.556961
48	H	0.940793	-5.360206	-2.265042
49	H	3.395285	-5.455252	-1.864663
50	H	4.550233	-3.530168	-0.868142
51	H	5.256732	-2.081613	0.520377
52	H	5.286668	2.184490	0.518887
53	H	4.548461	3.656360	-0.867137
54	H	3.391968	5.582045	-1.866165
55	H	0.937232	5.485092	-2.265052
56	H	-0.342756	3.501348	-1.554809
57	H	0.342715	3.501339	1.554922
58	H	-0.937302	5.485077	2.265149
59	H	-3.392026	5.582024	1.866189
60	H	-4.548485	3.656347	0.867109
61	H	-5.286637	2.184482	-0.518952
62	H	-5.256696	-2.081626	-0.520432
63	O	-6.671340	0.176764	-1.120766
64	O	6.671389	0.176798	1.120562
65	C	-7.396529	-1.012026	-1.387958
66	H	-6.896817	-1.628895	-2.144192
67	H	-7.547991	-1.606651	-0.479064
68	H	-8.364720	-0.691856	-1.770026
69	C	7.396669	-1.012008	1.387392
70	H	7.547831	-1.606549	0.478390
71	H	8.364984	-0.691867	1.769172
72	H	6.897201	-1.628951	2.143732
73	Bq	0.000000	0.062000	0.000000
74	Bq	-0.405454	0.062375	-0.914115
75	Bq	0.405454	0.061625	0.914115
76	Bq	-2.033500	1.277500	0.381000
77	Bq	-2.232019	1.170329	1.355220
78	Bq	-1.834981	1.384671	-0.593220
79	Bq	-2.095333	3.590000	1.159000
80	Bq	-1.938136	4.020198	0.270058
81	Bq	-2.252531	3.159802	2.047942
82	Bq	-4.071000	0.062333	-0.176333
83	Bq	-3.754139	0.063371	-1.124805
84	Bq	-4.387861	0.061295	0.772138

8π-*diaza*-HBC **7c**

Tag	Symbol	X	Y	Z
1	C	-1.219937	-0.689223	-0.015620
2	C	-1.220069	0.688995	-0.015531
3	N	-0.000137	1.421381	0.047740
4	C	1.219941	0.689217	-0.015574
5	C	1.220064	-0.689000	-0.015586
6	N	0.000136	-1.421390	0.047589
7	C	0.000258	-2.802262	0.243892
8	C	2.457366	-1.412562	-0.116340

9	C	2.457087	1.413008	-0.116515
10	C	-0.000256	2.802267	0.243953
11	C	-2.457374	1.412553	-0.116274
12	C	-2.457080	-1.413015	-0.116571
13	C	-1.219000	3.511324	0.336825
14	C	-1.194196	4.856801	0.717814
15	C	-0.000449	5.525842	0.939495
16	C	1.193383	4.857033	0.717568
17	C	1.218366	3.511565	0.336570
18	C	2.469923	2.830015	0.002486
19	C	3.653675	3.525087	-0.205886
20	C	4.845910	2.846855	-0.475141
21	C	4.861496	1.472434	-0.523049
22	C	3.679034	0.721478	-0.338882
23	C	3.679202	-0.720818	-0.338624
24	C	4.861899	-1.471553	-0.522188
25	C	4.846584	-2.845970	-0.474118
26	C	3.654391	-3.524420	-0.205224
27	C	2.470465	-2.829552	0.002846
28	C	1.218996	-3.511313	0.336807
29	C	1.194192	-4.856736	0.717994
30	C	0.000439	-5.525739	0.939778
31	C	-1.193394	-4.856964	0.717741
32	C	-1.218360	-3.511552	0.336538
33	C	-2.469890	-2.830029	0.002335
34	C	-3.653598	-3.525134	-0.206176
35	C	-4.845882	-2.846896	-0.475210
36	C	-4.861517	-1.472468	-0.522891
37	C	-3.679043	-0.721495	-0.338840
38	C	-3.679203	0.720802	-0.338630
39	C	-4.861883	1.471531	-0.522305
40	C	-4.846601	2.845944	-0.474065
41	C	-3.654448	3.524386	-0.204993
42	C	-2.470490	2.829539	0.002969
43	H	-2.128820	5.381899	0.861046
44	H	-0.000516	6.558732	1.265322
45	H	2.127938	5.382305	0.860607
46	H	3.661302	4.605891	-0.172604
47	H	5.759854	3.407017	-0.635977
48	H	5.798859	0.967061	-0.705827
49	H	5.799265	-0.965995	-0.704431
50	H	5.760715	-3.405956	-0.634507
51	H	3.662182	-4.605222	-0.171894
52	H	2.128827	-5.381791	0.861320
53	H	0.000505	-6.558568	1.265799
54	H	-2.127971	-5.382177	0.860873
55	H	-3.661128	-4.605947	-0.173102
56	H	-5.759834	-3.407055	-0.636015
57	H	-5.798949	-0.967092	-0.705301

58	H	-5.799193	0.965983	-0.704866
59	H	-5.760723	3.405934	-0.634494
60	H	-3.662307	4.605181	-0.171506
61	Bq	0.000000	0.000000	0.005333
62	Bq	0.000000	0.000000	1.005333
63	Bq	0.000000	0.000000	-0.994667
64	Bq	-2.452000	0.000000	-0.157167
65	Bq	-2.321765	0.000000	-1.148650
66	Bq	-2.582235	0.000000	0.834316
67	Bq	-1.227667	2.111000	0.083333
68	Bq	-1.162179	2.251216	-0.904619
69	Bq	-1.293154	1.970784	1.071286
70	Bq	-3.661500	2.134333	-0.275500
71	Bq	-3.828997	2.080861	0.708921
72	Bq	-3.494003	2.187806	-1.259921
73	Bq	-0.000333	4.177500	0.548833
74	Bq	0.042141	3.976444	1.527492
75	Bq	-0.042808	4.378556	-0.429825

7π-*diaza*-HBC **7c**

**Tag**	**Symbol**	**X**	**Y**	**Z**
1	C	-1.210238	-0.697998	-0.016832
2	C	-1.211225	0.696300	-0.016771
3	N	-0.000998	1.412584	0.049404
4	C	1.210238	0.698000	-0.016834
5	C	1.211225	-0.696298	-0.016767
6	N	0.000998	-1.412581	0.049412
7	C	0.001974	-2.812774	0.234530
8	C	2.451425	-1.417447	-0.110913
9	C	2.449397	1.420893	-0.111267
10	C	-0.001974	2.812780	0.234510
11	C	-2.451428	1.417446	-0.110912
12	C	-2.449395	-1.420894	-0.111269
13	C	-1.224012	3.514510	0.327242
14	C	-1.199527	4.864850	0.693317
15	C	-0.003793	5.531965	0.904133
16	C	1.192820	4.866555	0.692910
17	C	1.219091	3.516255	0.326825
18	C	2.465682	2.833555	0.012047
19	C	3.656488	3.529289	-0.183523
20	C	4.838740	2.849945	-0.459057
21	C	4.846572	1.469764	-0.519827
22	C	3.667900	0.726693	-0.338413
23	C	3.668961	-0.721551	-0.338073
24	C	4.848782	-1.462955	-0.518825
25	C	4.842965	-2.843121	-0.457519
26	C	3.661617	-3.524100	-0.182159
27	C	2.469726	-2.830053	0.012806
28	C	1.224011	-3.514504	0.327268
29	C	1.199527	-4.864836	0.693371

30	C	0.003793	-5.531946	0.904204
31	C	-1.192820	-4.866542	0.692962
32	C	-1.219090	-3.516249	0.326850
33	C	-2.465677	-2.833556	0.012044
34	C	-3.656474	-3.529296	-0.183558
35	C	-4.838721	-2.849956	-0.459126
36	C	-4.846559	-1.469775	-0.519886
37	C	-3.667896	-0.726698	-0.338429
38	C	-3.668964	0.721546	-0.338060
39	C	-4.848795	1.462944	-0.518769
40	C	-4.842984	2.843110	-0.457452
41	C	-3.661631	3.524094	-0.182125
42	C	-2.469731	2.830052	0.012809
43	H	-2.130035	5.396633	0.831166
44	H	-0.004480	6.569138	1.215291
45	H	2.122628	5.399641	0.830461
46	H	3.671728	4.608914	-0.139209
47	H	5.755939	3.404169	-0.617312
48	H	5.783049	0.966519	-0.709605
49	H	5.784588	-0.958399	-0.708430
50	H	5.761035	-3.396054	-0.615231
51	H	3.678437	-4.603685	-0.137481
52	H	2.130036	-5.396615	0.831230
53	H	0.004480	-6.569112	1.215387
54	H	-2.122630	-5.399623	0.830522
55	H	-3.671708	-4.608921	-0.139247
56	H	-5.755911	-3.404183	-0.617416
57	H	-5.783031	-0.966533	-0.709696
58	H	-5.784605	0.958384	-0.708342
59	H	-5.761062	3.396040	-0.615129
60	H	-3.678458	4.603678	-0.137443
61	Bq	0.000000	0.000000	0.005000
62	Bq	0.000000	0.000000	1.005000
63	Bq	0.000000	0.000000	-0.995000
64	Bq	-2.443000	-0.001833	-0.155333
65	Bq	-2.313507	-0.001740	-1.146914
66	Bq	-2.572493	-0.001926	0.836247
67	Bq	-1.226500	2.114000	0.082667
68	Bq	-1.161685	2.245468	-0.906533
69	Bq	-1.291315	1.982532	1.071866
70	Bq	-3.657333	2.133167	-0.265667
71	Bq	-3.825912	2.069421	0.717958
72	Bq	-3.488755	2.196912	-1.249292
73	Bq	-0.003000	4.184667	0.529833
74	Bq	-0.002887	3.909568	1.491249
75	Bq	-0.003113	4.459766	-0.431583

6π-*diaza*-HBC **7c**

Tag	Symbol	X	Y	Z
1	C	0.708417	-1.204272	-0.024783

2	C	-0.709415	-1.203696	-0.024689
3	N	-1.405927	0.000577	0.050173
4	C	-0.708418	1.204272	-0.024785
5	C	0.709414	1.203696	-0.024687
6	N	1.405926	-0.000577	0.050178
7	C	2.809878	-0.001170	0.228401
8	C	1.424107	2.441537	-0.110108
9	C	-1.422088	2.442674	-0.110572
10	C	-2.809880	0.001170	0.228390
11	C	-1.424107	-2.441538	-0.110110
12	C	1.422088	-2.442673	-0.110571
13	C	-3.515263	-1.224992	0.326558
14	C	-4.863474	-1.196194	0.696524
15	C	-5.524702	0.002413	0.909379
16	C	-4.862530	1.200395	0.695935
17	C	-3.514288	1.227935	0.325993
18	C	-2.835184	2.465493	0.014753
19	C	-3.530218	3.661761	-0.175859
20	C	-2.847025	4.834177	-0.458262
21	C	-1.461178	4.835107	-0.525146
22	C	-0.723537	3.660989	-0.341735
23	C	0.726595	3.660446	-0.341286
24	C	1.465202	4.834089	-0.523846
25	C	2.851013	4.832099	-0.456276
26	C	3.533195	3.659043	-0.174099
27	C	2.837180	2.463219	0.015742
28	C	3.515260	1.224992	0.326571
29	C	4.863469	1.196194	0.696548
30	C	5.524694	-0.002413	0.909410
31	C	4.862525	-1.200395	0.695958
32	C	3.514286	-1.227934	0.326006
33	C	2.835184	-2.465491	0.014755
34	C	3.530221	-3.661756	-0.175867
35	C	2.847029	-4.834170	-0.458284
36	C	1.461182	-4.835102	-0.525167
37	C	0.723538	-3.660988	-0.341741
38	C	-0.726593	-3.660448	-0.341282
39	C	-1.465198	-4.834093	-0.523827
40	C	-2.851008	-4.832106	-0.456256
41	C	-3.533192	-3.659048	-0.174092
42	C	-2.837180	-2.463221	0.015739
43	H	-5.399569	-2.123217	0.837769
44	H	-6.560971	0.002896	1.223961
45	H	-5.397879	2.127917	0.836741
46	H	-4.609179	3.682973	-0.127146
47	H	-3.394952	5.754584	-0.619040
48	H	-0.959712	5.770899	-0.721879
49	H	0.964526	5.770347	-0.720369
50	H	3.399703	5.752169	-0.616372

51	H	4.612149	3.679430	-0.124932
52	H	5.399562	2.123217	0.837797
53	H	6.560961	-0.002896	1.224001
54	H	5.397872	-2.127918	0.836768
55	H	4.609182	-3.682966	-0.127154
56	H	3.394958	-5.754574	-0.619075
57	H	0.959718	-5.770892	-0.721913
58	H	-0.964520	-5.770354	-0.720338
59	H	-3.399697	-5.752179	-0.616340
60	H	-4.612146	-3.679437	-0.124925
61	Bq	0.000000	0.000000	0.000000
62	Bq	0.000000	0.000000	1.000000
63	Bq	0.000000	0.000000	-1.000000
64	Bq	-0.001000	-2.435667	-0.159000
65	Bq	-0.001000	-2.307708	-1.150780
66	Bq	-0.001000	-2.563625	0.832780
67	Bq	-2.116833	-1.222000	0.081000
68	Bq	-2.242335	-1.156243	-0.908912
69	Bq	-1.991332	-1.287757	1.070912
70	Bq	-2.139500	-3.648333	-0.264833
71	Bq	-2.070485	-3.819617	0.717968
72	Bq	-2.208515	-3.477050	-1.247635
73	Bq	-4.181667	0.001667	0.530500
74	Bq	-3.903382	0.001945	1.490999
75	Bq	-4.459951	0.001389	-0.429999

Supplementary References

1. Berger, R., Wagner M., Feng X. L., Müllen K. Polycyclic aromatic azomethine ylides: a unique entry to extended polycyclic heteroaromatics. *Chem. Sci.* **6,** 436-441 (2015).
2. Dolomanov O. V., Bourhis, L. J., Gildea, R. J., Howard, J. A. K., Puschmann, H. Olex2: A Complete Structure Solution, Refinement and Analysis Program. *J. Appl. Cryst.,* **42**, 339-341 (2009).
3. Sheldrick, G.M. SHELXT–Integrated space-group and crystal-structure determination. *Acta Cryst. A*, 71, 3-8 (2015).
4. Sheldrick, G.M. Crystal structure refinement with SHELXL. *Acta Cryst. C*, 71, 3-8 (2015).
5. Cao, J. *et al*. The Impact of Antiaromatic Subunits in [4*n*+2] π-Systems: Bispentalenes with [4*n*+2] π-Electron Perimeters and Antiaromatic Character. *J. Am. Chem. Soc.*, 137, 7178–7188 (2015).
